# Copper-Catalyzed α-Alkylation of Aryl Acetonitriles with Benzyl Alcohols

**DOI:** 10.1021/acs.joc.4c01662

**Published:** 2024-09-18

**Authors:** Marianna Danopoulou, Leandros P. Zorba, Athanasia P. Karantoni, Demeter Tzeli, Georgios C. Vougioukalakis

**Affiliations:** † Laboratory of Organic Chemistry, National and Kapodistrian University of Athens, Panepistimiopolis, 15771 Athens, Greece; ‡ Laboratory of Physical Chemistry, National and Kapodistrian University of Athens, Panepistimiopolis, 15771 Athens, Greece; § Theoretical and Physical Chemistry Institute, National Hellenic Research Foundation, Vas. Constantinou, 48, 11635 Athens, Greece

## Abstract

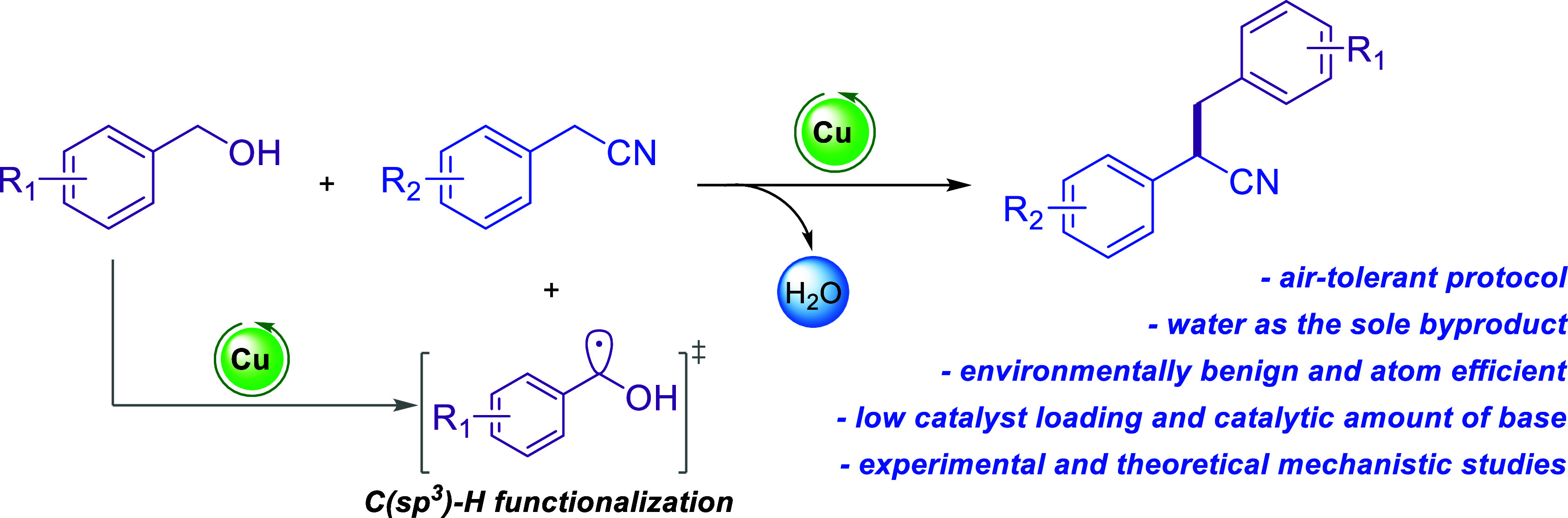

A highly efficient, *in situ* formed CuCl_2_/TMEDA catalytic system (TMEDA = *N*,*N*,*N*′,*N*′-tetramethylethylene-diamine) for the cross-coupling reaction of aryl acetonitriles with benzyl alcohols is reported. This user-friendly protocol, employing a low catalyst loading and a catalytic amount of base, leads to the synthesis of α-alkylated nitriles in up to 99% yield. Experimental mechanistic investigations reveal that the key step of this transformation is the C(sp^3^)–H functionalization of the alcohol, taking place *via* a hydrogen atom abstraction, with the simultaneous formation of copper-hydride species. Detailed density functional theory studies shed light on all reaction steps, confirming the catalytic pathway proposed on the basis of the experimental findings.

## Introduction

In recent years, considerable efforts have been made toward achieving step and atom economies in organic synthesis, primarily by focusing on environmentally benign processes through the use of abundant, low-cost transition metals, as well as *via* the reduction of undesired or toxic byproducts.^1−3^ Despite the numerous related breakthroughs, a vast number of organic transformations still rely on the use of toxic, precious, and scarce transition metals, with the design of more environmentally benign approaches remaining a key goal in catalysis.^1−3^

**Scheme 1:**
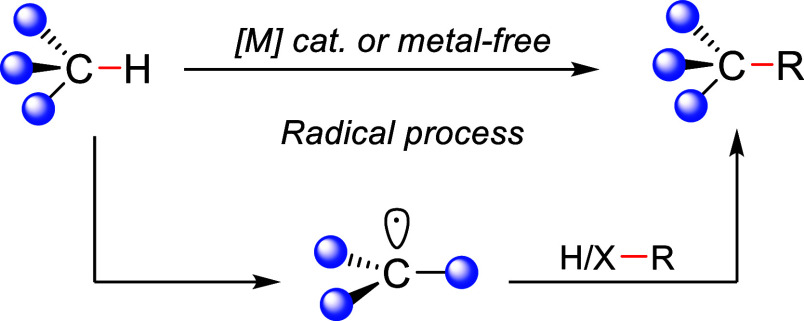
C–H Functionalization

Transition metal-catalyzed C(sp^3^)–H activation represents a highly desired yet challenging strategy toward C–C bond formation, mainly due to the limited reactivity of most C(sp^3^)–H bonds.^4,5^ One of the classical methods for the functionalization of C(sp^3^)–H bonds involves the formation of free radicals *via* a hydrogen atom abstraction step (Scheme 1).^6^ This approach has enabled the synthesis of useful organic molecules and macromolecules,^7−9^ as well as late-stage diversification in drug discovery,^10,11^ by avoiding the activation of the substrate and also limiting the generation of undesired waste.^12^

α-Alkylated nitriles are a class of nitrogen-containing molecules of particular interest, with numerous applications in the chemical and pharmaceutical industries.^13^ The cyanide moiety can be easily transformed into a number of valuable functional groups, such as amide,^14^ amine,^15^ acid,^16^ ketone,^17^ oxazoline,^18^ and thiazoline.^19^ Therefore, the development of new, efficient catalytic protocols for the synthesis of α-alkylated nitriles is attracting significant interest. Traditional methods for the synthesis of α-alkylated nitriles require the use of toxic alkyl halides as alkylating agents, often also leading to the generation of harmful byproducts.^20−22^

**Scheme 2:**
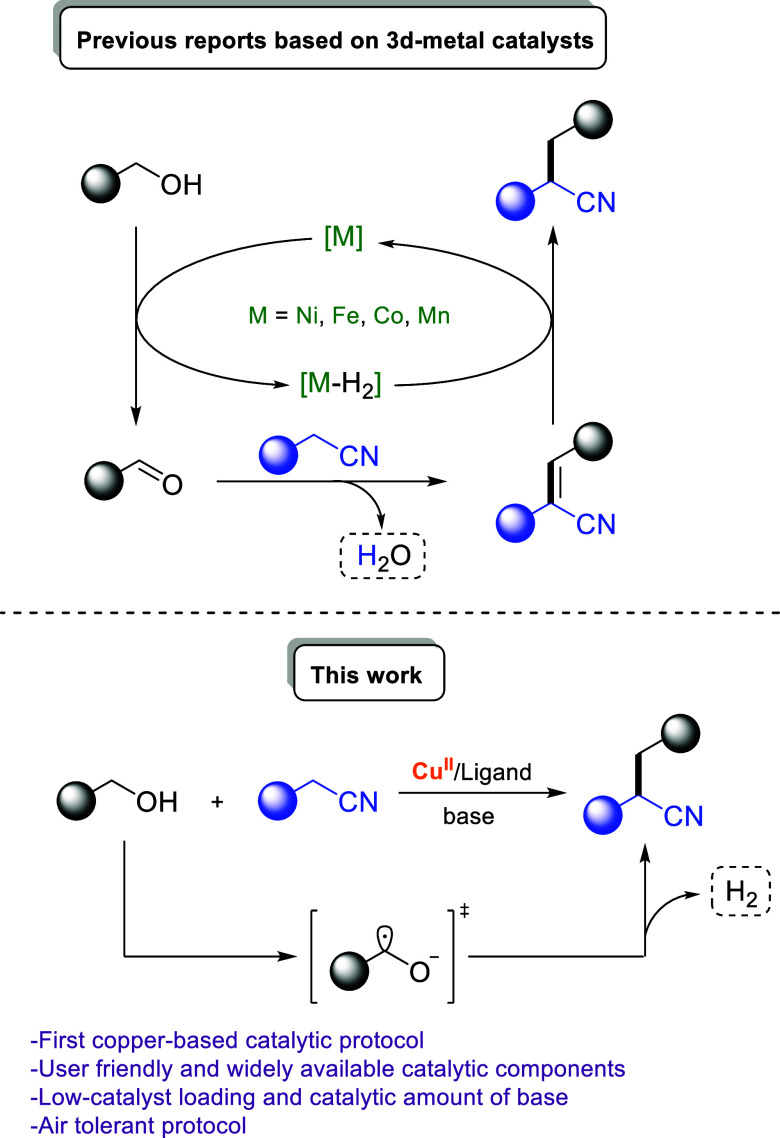
Catalytic α-Alkylation of Nitriles with Alcohols

An efficient approach to α-alkylated nitriles is *via* borrowing-hydrogen (BH) catalysis, employing alcohols as coupling partners.^23^ In principle, this method generates water as the sole byproduct.^24^ In a typical borrowing-hydrogen process, the metal catalyst dehydrogenates the alcohol to the corresponding aldehyde by “borrowing” a hydride and a proton (Scheme 2). The aldehyde undergoes nucleophilic attack by the nitrile substrate, following a typical Knoevenagel condensation, toward the formation of an α,β-unsaturated nitrile. This intermediate is subsequently hydrogenated to the α-alkylated nitrile product by the “borrowed” hydride and proton from the catalyst.^24^ Noble-metal catalysts based on Ru,^25−30^ Ir,^31−35^ Rh,^36,37^ Os,^38^ or Pd^39^ have been reported to be very efficient in borrowing-hydrogen catalysis.

Significant efforts have also been made to replace these precious metal catalysts with more sustainable and abundant ones based on Ni,^40−42^ Fe,^43,44^ Co,^45,46^ or Mn.^47^ However, most of the existing catalytic protocols require the use of pincer-type organometallic complexes, which are usually air-sensitive and costly; moreover, their synthesis is often challenging and achieved through multistep, time-consuming procedures. A base-mediated α-alkylation of nitriles using a stoichiometric amount of base has also been reported.^48^

Copper-mediated catalytic protocols for C–C bond formations through aerobic C(sp^3^)–H functionalization are omnipresent in organic synthesis, mainly owing to their environmentally benign character.^49^ Nevertheless, the cross-coupling reaction between nitriles and alcohols has not been achieved by employing copper catalysis thus far. Our continuous interest in the design and development of sustainable catalytic strategies, among others by employing copper-catalysis,^50−53^ led us to the development of a straightforward protocol for the synthesis of α-alkylated nitriles (Scheme 2).

Thus, we herein report the development of a novel, highly efficient, *in situ* formed copper-based catalytic system using the low-cost and readily available CuCl_2_ in combination with *N*,*N*,*N*′,*N*′-tetramethylethylenediamine (TMEDA) as a ligand for the synthesis of α-alkylated nitriles.

Based on a series of control, kinetic, and radical scavenging/trapping experiments as well as thorough density functional theory (DFT) calculations, we propose a mechanism involving the C(sp^3^)–H functionalization of the benzylic alcohol, followed by the formation of copper-hydride species and subsequent oxidation of the alcohol substrate.

## Results and Discussion

Phenylacetonitrile **1a** (0.5 mmol) and benzyl alcohol **2a** (1 mmol) were chosen as benchmark substrates for optimization of the reaction conditions (Table 1). Based on previous works employing other metal catalysts in analogous transformations,^46^ we began by testing several copper salts at 5 mol % catalyst loading, along with 30 mol % of *t*-BuOK in toluene, at 130 °C for 18 h. A very low catalytic activity was recorded under these conditions toward the formation of the desired product **3a** (entries 1–4).

**Table 1:**
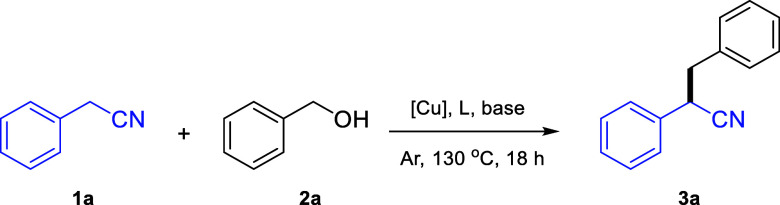

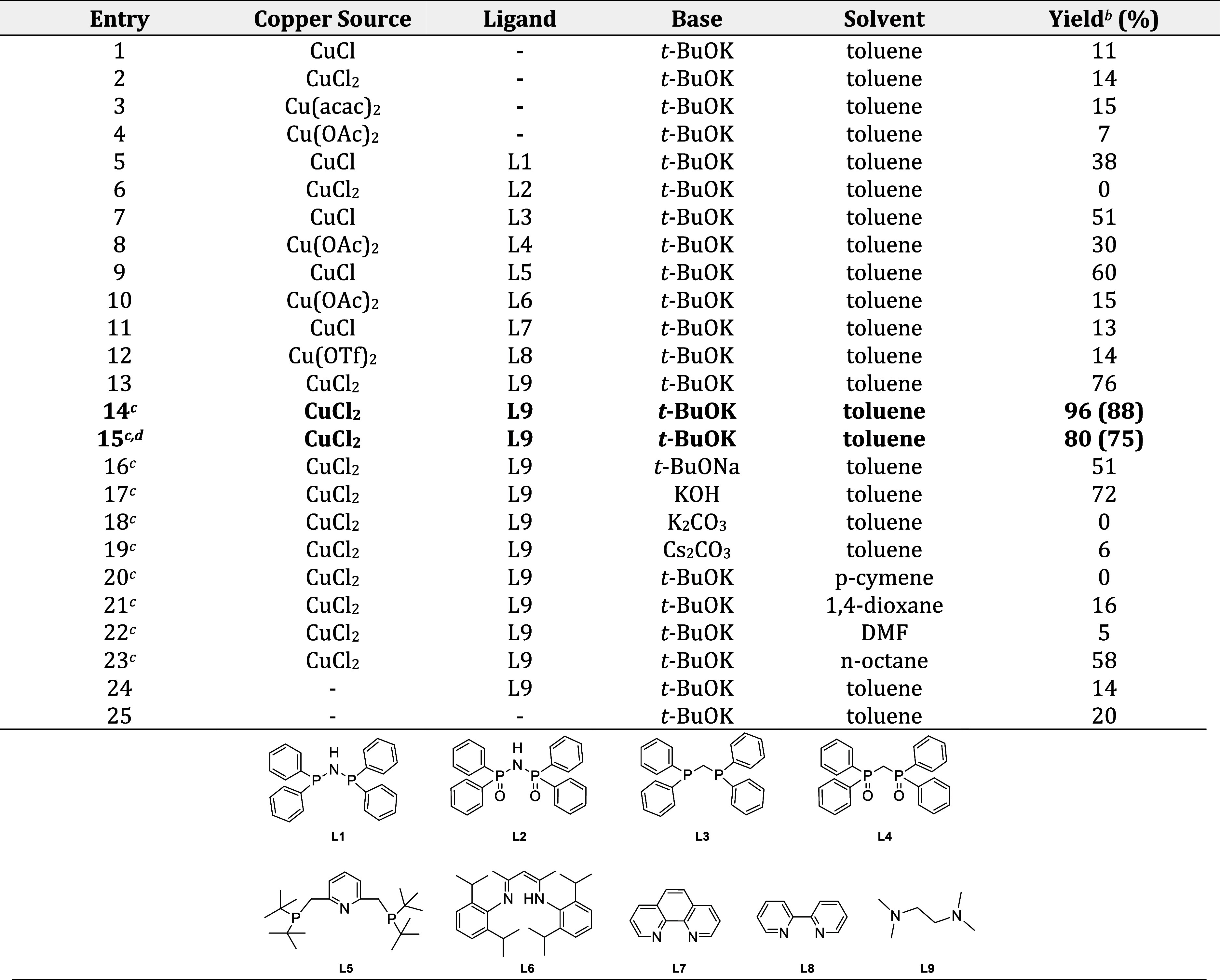
Optimization of the Reaction Conditions^a^

a Reaction conditions: **1a** (0.5 mmol), **2a** (1 mmol), copper source (5 mol %), ligand (5 mol %), base (30 mol %), and the solvent (1 mL) were heated in a J. Young tube at 130 °C for 18 h under an Ar atmosphere.

b Yields were calculated by analyzing the ^1^H NMR spectra of the crude reaction mixtures using mesitylene (0.5 mmol) as an internal standard (IS) (isolated yields in parentheses).

c Reaction mixture was heated at 140 °C for 24 h.

d Reaction was performed under air.

We then investigated the impact of several ligands with architectures that had proved to be beneficial in related reactions.^40,46,54^ The use of CuCl along with bis(diphenylphosphino)amine **L1** led to a 38% yield of desired nitrile **3a** (entry 5). The imidodiphosphinate ligand **L2** was also used, in combination with CuCl_2_, albeit not providing **3a** (entry 6). Employing bis(diphenylphosphino)methane **L3** in combination with CuCl or its corresponding phosphine oxide **L4** with Cu(OAc)_2_ led to moderate yields of 51% and 30%, respectively (entries 7 and 8). The pyridyl-cored PNP ligand **L5** was also employed with CuCl, resulting in a very good yield of the desired coupling product (60%, entry 9). However, taking into consideration that the synthesis of **L5** requires a multistep procedure and it is air-sensitive, we decided to search further for a more widely available and easy-to-handle ligand.

Inspired by recent works employing other metal catalysts along with nitrogen-based ligands in borrowing-hydrogen transformations,^40,42,46^ the bench-stable β-diketiminate ligand **L6** was employed with Cu(OAc)_2_, leading to a 15% yield of **3a** (entry 10). We then focused on commercially available ligands, such as 1,10-phenanthroline **L7** and 2,2-bipyridine (Bpy—**L8**), which provided very poor results (entries 11 and 12). Surprisingly, when tetramethylethylenediamine (TMEDA—**L9**) was used, along with anhydrous CuCl_2_, a 76% yield of **3a** was obtained.

It has to be noted that benzaldehyde and α,β-unsaturated nitrile species were observed in all ^1^H NMR spectra of the above crude reaction mixtures for reactions efficiently providing **3a**, however, in limited amounts. Upon increasing the reaction temperature to 140 °C and the reaction time to 24 h, an excellent product yield of 96% was obtained (deduced by ^1^H NMR analysis of the crude mixture), leading to an 88% isolated yield after chromatographic purification (entry 14).

Copper-based catalytic systems employing nitrogen bidentate ligands such as Bpy^55−58^ and TMEDA^59,60^ are known for their excellent catalytic activity in the aerobic oxidation of benzylic alcohols in the presence of nitroxyl radicals as cocatalysts.^61^ The initial step of this transformation is proposed to be the oxidation of the alcohol through a hydrogen autotransfer mechanism.^54,62^ Having this type of reactivity in mind and in order to study the efficiency of our optimized protocol in the presence of oxygen, an under-air reaction was set up, affording the desired product **3a** in 80% yield (entry 15). To the best of our knowledge, this is the first time that an earth-abundant 3d metal-catalyst is shown to be tolerant to open-air conditions for this type of cross-coupling transformation.

After various bases were purged (entries 16–19), *t*-BuOK was found to be the most suitable. The use of polar aprotic solvents, such as 1,4-dioxane or DMF, inhibits the progress of the reaction, providing only traces of the desired product (entries 21 and 22). Interestingly, upon replacing toluene with *p*-cymene, the reaction is completely hindered (entry 20). Additional reaction temperatures as well as catalysts and base loadings were also screened (see Supporting Information, Tables S2, S3, and S6), not leading to an improvement in the reaction outcome. Carrying out the reaction under the optimal reaction conditions but in the absence of the copper source (entry 24) resulted in limited formation of **3a**. The same result was obtained when the reaction was carried out only in the presence of a base (entry 25).

With the optimized conditions in hand, we explored the reactivity of a series of nitriles and alcohols (Table 2). Nitriles bearing electron-donating groups at the *meta* or *para* position, *i.e.*, *m*-Me (**1b**) and *p*-OMe (**1c**), were successfully coupled to benzyl alcohol **2a**, providing very good isolated yields of **3b** and **3c** (71% and 80%, respectively). Performing the same transformations under air afforded 84% and 80% yields of **3b** and **3c**, respectively. *p*-F- and *p*-Cl-phenyl acetonitrile are also amenable to our protocol; however, **3d** required an additional reaction time of 36 h (57% isolated yield). **3e** was also obtained in a very good, 73% isolated yield. *p*-F-phenyl acetonitrile was efficiently coupled with benzyl alcohol, even when the reaction was performed under air for 24 h, leading to a 52% yield of **3d**. When the synthesis of **3e** was attempted under air, it afforded a poor, 20% yield, calculated by ^1^H NMR analysis using an IS. Further nitrile scope studies included 3,4-dimethoxyphenylacetonitrile and 3,4-(methylenedioxy)phenyl acetonitrile in their reactions with benzyl alcohol. In both cases, the corresponding nitriles **3f** and **3g** were obtained in very good isolated yields, 67% and 81%, respectively.

**Table 2:**
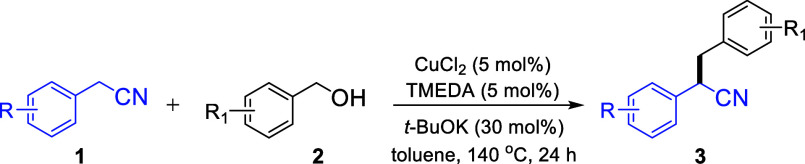

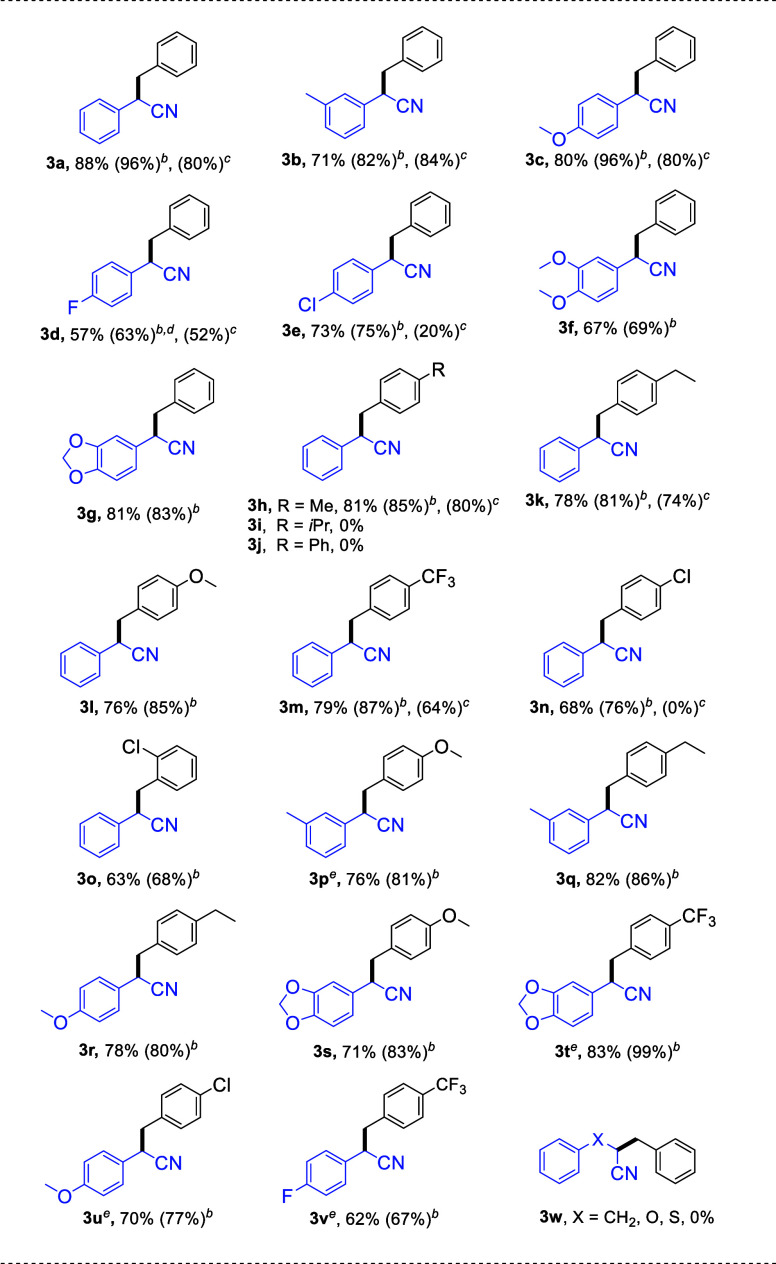
Substrate Scope of the Reaction^a^^,^^b^^,^^c^^,^^d^^,^^e^

a Reaction conditions: **1** (0.5 mmol), **2** (1 mmol), CuCl_2_ (5 mol %), TMEDA (5 mol %), *t*-BuOK (30 mol %), and toluene (1 mL) in a J. Young tube at 140 °C for 24 h under Ar.

b Yield in parentheses is calculated using mesitylene as an IS, and the reaction was performed under Ar.

c Yield in parentheses is calculated using mesitylene as an IS, and the reaction was performed under air.

d Reaction was heated for 36 h under Ar.

e Reaction was performed with 50 mol % of *t*-BuOK.

A series of substituted benzylic alcohols was also probed (Table 2). *p*-Substituted benzylic alcohols bearing electron-donating groups (−Me, −Et, and −OMe) were successfully coupled with phenyl acetonitrile toward the corresponding nitriles in very good yields of 76–81% (**3h**, **3k**, and **3l**). The same transformations provided similar results when the reactions were carried out under a noninert atmosphere. On the other hand, no product was obtained upon reacting phenylacetonitrile with *p*-isopropylbenzyl alcohol **2i** or biphenyl-4-methanol **2j** (targeted products **3i** and **3j**). The fact that a substrate (**2i**) bearing a simultaneously benzylic and tertiary hydrogen atom is not amenable to coupling, along with the observed incompatibility of *p*-cymene as the solvent, mentioned above, led us to the conclusion that free-radical species may be involved in the transformation. In other words, we reasoned that the benzylic isopropyl groups of *p*-cymene and **2i**, which can easily lead to free radicals *via* hydrogen atom abstraction, hinder the reaction by irreversibly reacting with key intermediates or catalytic species.

Employing *p*-trifluoromethylbenzyl alcohol, featuring a strongly electron-withdrawing group, led to a 79% isolated yield of desired product **3m** when the reaction was performed under inert conditions. Performing the reaction under noninert conditions led to a reduced **3m** yield (64% calculated by NMR). Upon using *p*-Cl- and *m*-Cl-benzylic alcohols, we also obtained the corresponding products (**3n** and **3o**, respectively) in very good yields.

Interestingly, the reaction of *p*-Cl-benzylic alcohol with phenylacetonitrile under air did not lead to product formation. In this case, the ^1^H NMR spectrum of the crude mixture revealed the existence of benzaldehyde and 4-chlorobenzaldehyde, suggesting the involvement of a free-radical mechanism resulting in the dehalogenation of *p*-Cl-benzylic alcohol. Additional experiments were conducted by employing a variety of different nitrile and benzyl alcohol combinations, for example, leading to the formation of coupling products **3p–3r** in 76–82% yields.

Moreover, the reaction of 3,4-(methylenedioxy)-phenylacetonitrile with *p*-methoxybenzyl alcohol or *p*-trifluoromethylbenzyl alcohol afforded the corresponding products **3s** and **3t** in 71% and 83% isolated yield (83% and 99% NMR yield), respectively. The reaction between 3,4-dimethoxyphenylacetonitrile and 4-chlorobenzyl alcohol afforded **3u** in 70% isolated yield by using a 50 mol % loading of base. Similarly, the reaction between *p*-fluorophenylacetonitrile and *p*-trifluomethylbenzyl alcohol required a 50 mol % loading of base toward the halogenated product **3v** in 62% isolated yield.

On the other hand, the coupling of hydrocinnamonitrile, phenoxyacetonitrile, or (phenylthiol)acetonitrile was not possible under our optimal catalytic conditions (targeted products **3w**).

**Scheme 3:**
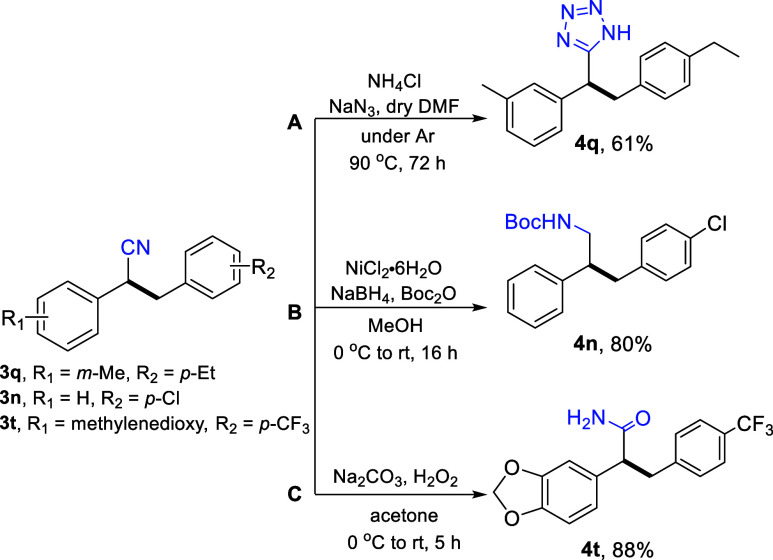
Nitrile Group Transformations on Coupling Products Derived through the Herein Developed Catalytic Protocol

The nitrile moiety can be easily transformed to a number of synthetically and biologically important functional groups. To highlight the synthetic utility of our herein developed protocol, we converted the nitrile groups of **3q**, **3n**, and **3t** into three useful functionalities shown in Scheme 3.

In specific, compound **3q** was converted to the corresponding tetrazole by simply adding NaN_3_ and NH_4_Cl in a solution of **3q** in DMF at 90 °C for 72 h under an inert atmosphere. The resulting compound (**4q**) was isolated in 61% yield after column chromatographic purification. Nitrile **3n** was reduced to the corresponding Boc-protected amine **4n** in a one-pot, two-step reaction using NaBH_4_ along with NiCl_2_·6H_2_O and di-*tert*-butyl dicarbonate in 80% isolated yield *via* a simple filtration through a silica gel plug. Finally, **3t** was transformed into **4t** using Na_2_CO_3_ and H_2_O_2_ in acetone and was isolated in 88% yield without the need for chromatographic purification after the reaction workup.

**Figure 1:**
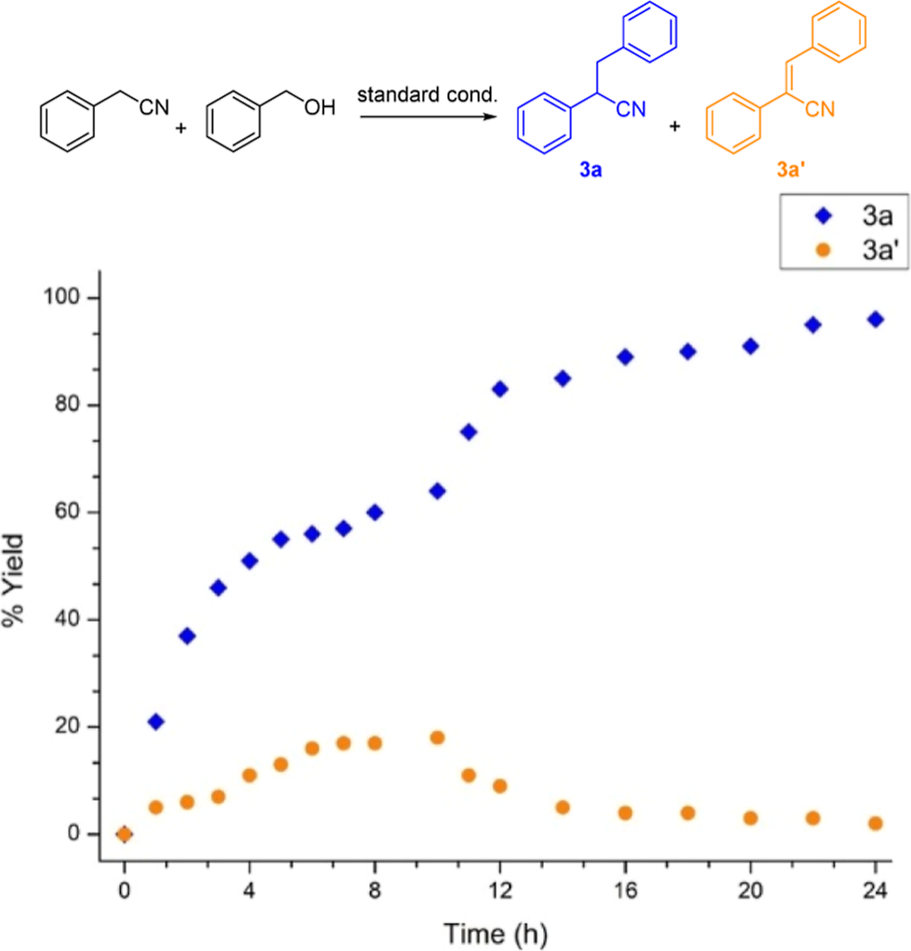
Monitoring of the reaction progress under the optimal conditions.

To obtain the kinetic profile of the herein described transformation, the progress of the reaction between phenylacetonitrile (**1a**) and benzyl alcohol (**2a**) was monitored by ^1^H NMR using 1,3,5-trimethoxybenzene as an IS (Figure 1). This study showed that the reaction initially provides both the α,β-unsaturated nitrile **3a′** and the desired product **3a**, with the yield of **3a** surpassing that of **3a′**. The yield of **3a′** reaches a maximum at about 10 h reaction time, after which it starts to decrease. A full conversion of the starting nitrile **1a** was observed at 12 h, when the yield of **3a** was measured at 80%. The reaction was completed after 24 h, when the yield of the desired product was found to be 95%. These findings suggest that the α,β-unsaturated nitrile **3a′** is an intermediate *en route* to the desired product **3a**.

**Scheme 4:**
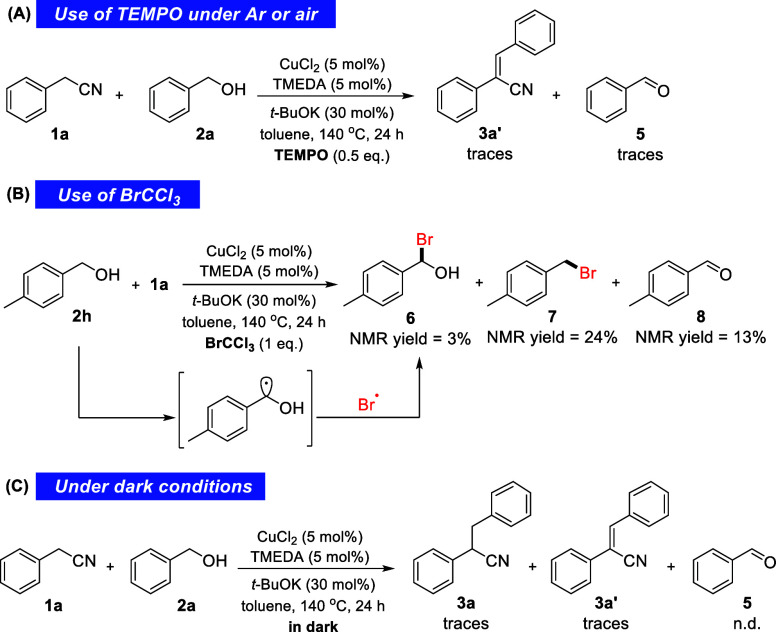
Experiments Designed to Probe the Intermediacy of Free Radicals

To further probe the mechanism of this transformation, we set up a reaction between phenylacetonitrile (**1a**) and benzyl alcohol (**2a**) in the presence of 0.5 equiv of TEMPO [(2,2,6,6-tetramethylpiperidin-1-yl)oxyl] under air or inert conditions, in both cases leading to a totally suppressed reaction (Scheme 4A). A careful analysis of the crude reaction mixtures’ ^1^H NMR spectra showed only traces of the α,β-unsaturated nitrile **3a′** and benzaldehyde (**5**). The quenching of the reaction in the presence of TEMPO suggests that radical species, crucial for the progress of the reaction, are involved.

Multiple attempts to either crystallize and analyze by X-ray crystallography possible TEMPO-trapped species or detect these through high-resolution mass spectrometry (HRMS) analysis of crude TEMPO-containing mixtures were not successful. The observed aldehyde traces most probably originate from the oxidation of benzyl alcohol under the reaction conditions, as is commonly observed in analogous systems.^25,26,30,41,45^

The formation of bromo(*p*-tolyl) methanol **6** in the presence of BrCCl_3_, which is known for its ability to brominate free-radical species, suggests the formation of a free radical on the benzylic carbon on the alcohol substrate (Scheme 4B). Bromo(*p*-tolyl) methanol **6** was detected (3%), along with benzyl bromide **7** (24%) and the corresponding aldehyde **8** (13%), upon analyzing the ^1^H NMR spectra of the crude mixture.

**Scheme 5:**
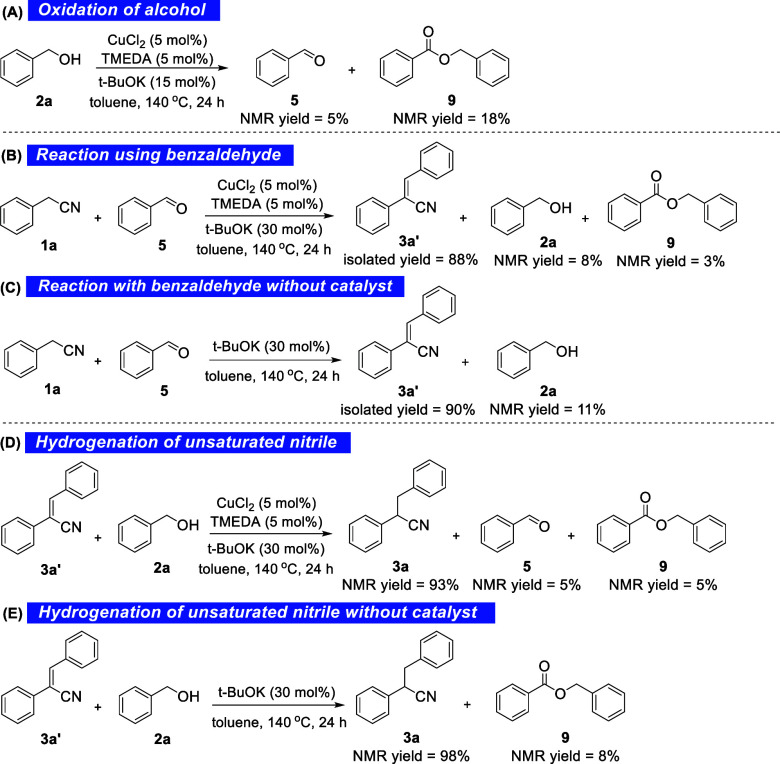
Control Experiments Carried Out to Shed More Light on the Mechanism of the Transformation

A reaction between **1a** and **2a** was also conducted under complete dark conditions (Scheme 4C), leading to only traces of **3a** and **3a′**. This observation suggests the occurrence of photochemically assisted homolytic bond cleavage. In the absence of phenylacetonitrile, under the optimal conditions, **2a** was converted into benzaldehyde **5** (5%) and benzyl benzoate **9** (18%), as quantified from the ^1^H NMR spectra of the crude reaction mixtures (Scheme 5A). The formation of **9**, which had not been observed during the optimization experiments, may originate from the dehydrogenative homocoupling of the alcohol in a base-mediated Tishchenko-type reaction.^63−68^ Furthermore, the reaction of phenylacetonitrile with benzaldehyde (derived from benzyl alcohol, as shown above) under the optimal conditions (Scheme 5B) led to an 88% NMR yield of the α,β-unsaturated nitrile **3a′**, obviously *via* a Knoevenagel condensation. This fact suggests once again that α,β-unsaturated nitriles are key intermediates toward the final α-alkylated nitriles. Benzyl benzoate **9** was again identified in small quantities (Scheme 5B), along with benzyl alcohol **2a**, which could originate from benzaldehyde *via* a Meerwein–Ponndorf–Verley (MPV) hydrogenation.^48,69−73^ Moreover, the condensation of benzaldehyde and phenylacetonitrile was shown to be feasible in the presence of a base alone at 30% loading (Scheme 5C), showing that the condensation step does not require the presence of another catalyst. Finally, reacting the isolated α,β-unsaturated nitrile **3a′** with benzyl alcohol **2a**, either under the optimal conditions (Scheme 5D) or by simply employing 30 mol % of *t*-BuOK (Scheme 5E), led to **3a** in 93% or 98% yield, respectively. Therefore, copper species are most probably not involved in the hydrogenation of α,β-unsaturated nitrile **3a′** toward the final α-alkylated nitrile **3a**.

## Proposed Mechanism and DFT Calculations

**Scheme 6:**
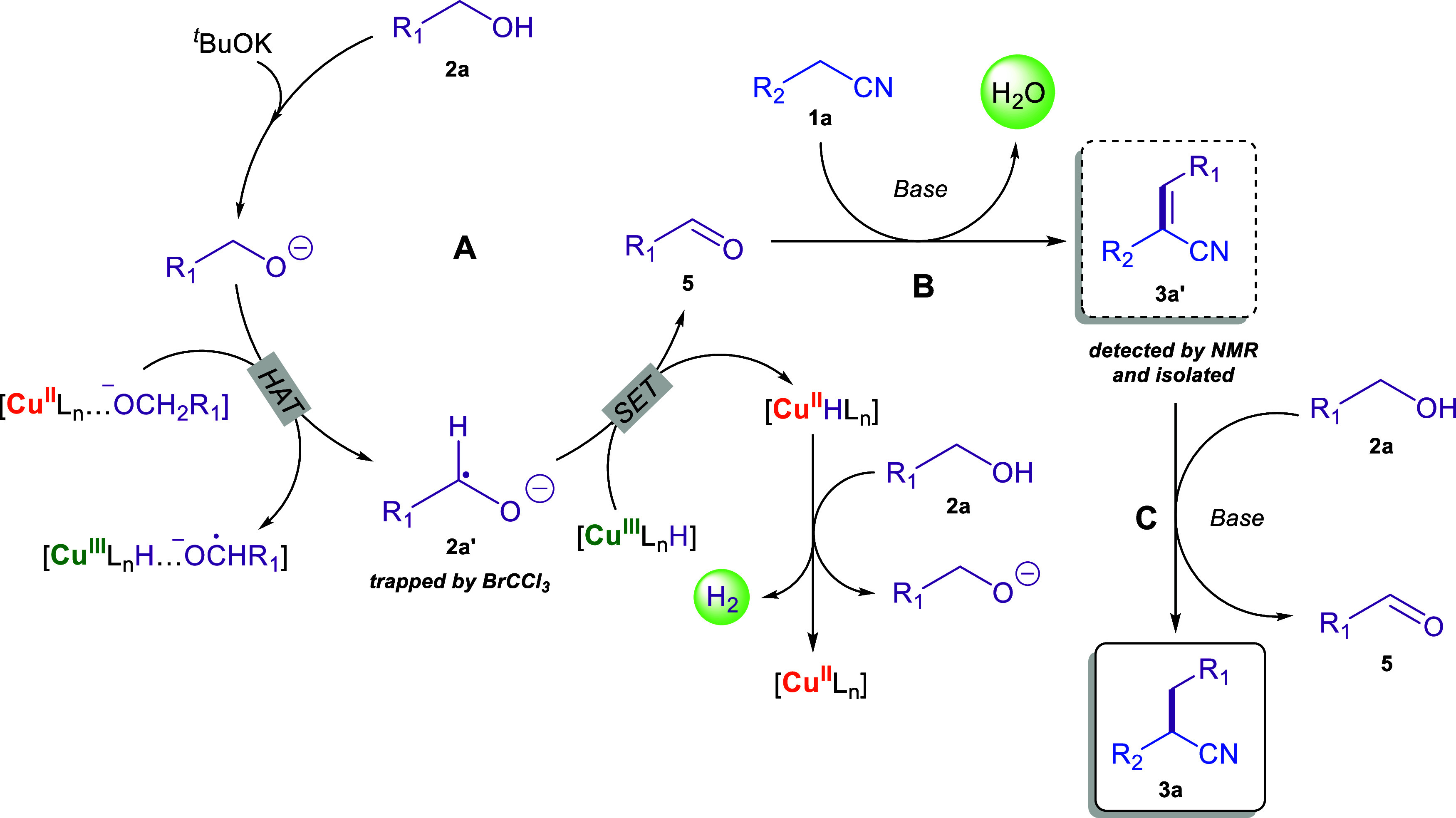
Proposed Catalytic Reaction Mechanism

Based on the above observations, a proposed mechanism for the transformation reported herein is shown in Scheme 6: initially, benzyl alcohol **2a** is deprotonated by the base, leading to the corresponding alkoxide. Cu^II^L_*n*_ species, under the high reaction temperature, catalyze the homolytic C(sp^3^)–H bond cleavage of the deprotonated alcohol substrate *via* a hydrogen atom transfer, leading to the formation of the key radical intermediate **2a′**, simultaneously generating Cu^III^–H species. Then, a single electron transfer from radical intermediate **2a′** to the Cu^III^–H species leads to the Cu^II^–H species, also affording benzaldehyde **5**. Molecular hydrogen is subsequently generated from the Cu^II^–H species and the acidic proton of the alcohol substrate **2a**. This path allows the regeneration of both the alkoxide anion and the Cu^II^L_*n*_ catalyst for the next catalytic cycle. Benzaldehyde **5** undergoes a nucleophilic attack by the deprotonated phenylacetonitrile **1a** to produce the α,β-unsaturated nitrile **3a′***via* a Knoevenagel condensation. Finally, the intermediate nitrile **3a′** is reduced to the saturated nitrile **3a***via* a base-mediated MPV hydrogenation step. The formation of water as the only byproduct along with the low catalyst and base loading renders the overall process particularly sustainable.

**Figure 2:**
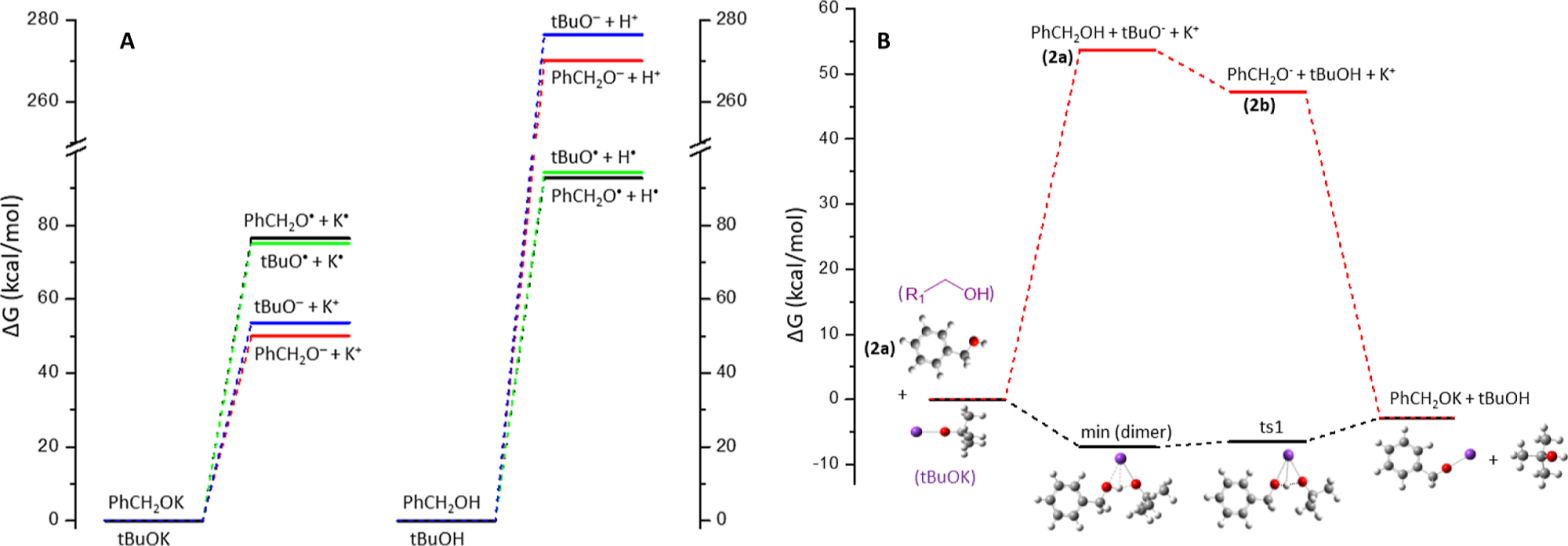
Reaction free energies of the (A) homolytic and heterolytic cleavage of *t*-BuO–K, *t*-BuO–H, PhCH_2_O–K, and PhCH_2_O–H bonds and (B) formation of PhCH_2_OK.

Given that this catalytic cycle has not been reported before, we also studied it thoroughly *via* DFT calculations. Minimum structures and transition states of the compounds involved in Scheme 6 were calculated *via* the wB97XD/6-311G+(d,p) methodology in a toluene solvent. The cleavage of PhCH_2_O–H is an endothermic reaction. While homolytic it has lower energy demands than heterolytic, it is still significantly endothermic (Figure 2A). Similarly, the cleavage of the O–K is an endothermic reaction, but for the O–K bond, the heterolytic cleavage has lower energy demands than the homolytic one (Figure 2A). The free Gibbs reaction energy of the heterolytic cleavage of the *t*-BuOK is 53.7 kcal/mol; however, *via* the formation of a PhCH_2_OH···*t*-BuOK dimer (Figure 2B), where a four-member ring is formed (O···H···O···K), the cleavage of the *t*-BuOK bond is energetically stabilized. Overall, the PhCH_2_OH + *t*-BuOK → PhCH_2_OK + *t*-BuOH reaction is exergonic (exothermic) with Δ*G* = −2.80 kcal/mol and Δ*H* = −4.94 kcal/mol at ambient conditions (see Tables 3 and S7, Supporting Information).

**Table 3:** Reaction Enthalpies Δ*H* (kcal/mol) and Free Reaction Energies Δ*G* (kcal/mol) at *T* = 298.15 K and *P* = 1 atm *via* wB97XD/6-311G+(d,p) Methodology in Toluene Solvent

	Δ*H*	Δ*G*
PhCH_2_OH + *t*BuOK → PhCH_2_OK + *t*BuOH	–4.94	–2.80
**2a** → H_2_ + **5**	13.87	6.20
**5** + **1a** → H_2_Ο + **3a′**	–0.55	–0.41
**2a** + **3a′** → **5** + **3a**	–13.91	–13.46

Ts0 also supports the importance of the potassium ion’s involvement in the reactions’ mechanism, revealing why *t*-BuOK and KOH are the most efficient bases in promoting this type of transformation.

Five copper complexes were studied as potential catalytic systems for this study (Scheme S1, Supporting Information). Detailed energy profiles are shown in Tables S7 and S8 (Supporting Information). Their reactions of formation are exergonic and exothermic. Moreover, the tetramethylethylenediamine ligand (L) forms stable complexes with both CuCl_2_ and Cu^2+^. While the Cu_2_L_2_Cl_4_ complex can be formed, it decomposes easily to CuLCl_2_, *i.e.*, the enthalpy of reaction Cu_2_L_2_Cl_4_ → 2CuLCl_2_ is exothermic by Δ*H* = −41.1 kcal/mol. Similarly, [Cu_2_L_2_]^4+^ can be decomposed. The enthalpy of the Cu^2+^ → [CuL]^2+^ reaction is −195.2 kcal/mol, and the complexation of one additional L, *i.e.*, [CuL]^2+^ + L → [CuL_2_]^2+^, is −97.7 kcal/mol.

The homolytic hydrogen additions at the CuL, [CuL]^2+^, and [CuL_2_]^2+^ complexes are exothermic reactions, with reaction enthalpies ranging from −20.3 to −84.4 kcal/mol (Table S7 and Figure S2, Supporting Information). The complexation of PhCH_2_O^–^ (**2b**) with the four used catalytic systems of Cu is an exergonic (exothermic) reaction with Δ*G*(Δ*H*) values ranging from −24.7(−36.1) kcal/mol for the CuLCl_2_ complex to 155.4(−168.5) kcal/mol for the Cu^II^L complex (Table S8, Supporting Information). In the benzyl alcohol substrate **2a** (in toluene), the C–C bond between the *ipso*-carbon of the Ph group and the methylene carbon of the –CH_2_OH group is 1.508 Å. This bond length is increased by about 0.03 Å in anion **2b**, while in the corresponding radical PhCH_2_O^•^, it is increased only by 0.006 Å. It is interesting to note that the abstraction of a H atom from the methylene group affects this C–C bond, *i.e.*, the C–C of **2a′** is shorter by 0.08 Å than the C–C of the **2b**.

The **2a**, **2b**, **2a′**, and **5** molecules linked to the catalytic systems present similar trends for the C–C bond distances (Table 4), although there are some differences with respect to the free ones. For instance, the **2a′** attached to the Cu^II^L or Cu^II^L_2_ results in a shorter C–C bond and an elongated C–O bond, compared to the free one, while **5** presents shorter C–O bonds than when free. Finally, the Cu–N bonds range from 1.9 to 2.2, and the formed Cu–O bonds range from 1.82 to 1.92 Å.

**Table 4:** Geometry of the **2a**, **2a′**, **2b**, and **5** Molecules in Toluene Solvent and Attached or Linked at the CuL_*x*_Cl_*y*_ Complexes^b^

	PhCH_2_O^–^ (**2b**)			PhCHO^–^ (**2a′**)			PhCHO (**5**)		
	C–C^a^	C–O	CCO	C–C^a^	C–O	CCO	C–C^a^	C–O	CCO
toluene	1.541	1.338	116.0	1.466	1.266	126.7	1.479	1.248	120.8
–CuL	1.519	1.404	108.9	1.414	1.304	125.6	1.486	1.207	123.9
–CuL_2_	1.521	1.377	126.7	1.407	1.343	120.3			
–CuLCl_2_	1.513	1.399	126.2	1.466	1.221	124.8	1.473	1.213	124.5
–Cu_2_L_2_	1.498	1.460	111.8	1.421	1.270	125.4			

	PhCH_2_OH (**2a**)			PhCH_2_O^•^		
	C–C^a^	C–O	CCO	C–C^a^	C–O	CCO
toluene	1.508	1.415	110.2	1.514	1.350	117.5
–CuL	1.487	1.498	107.3			

a C–C distance between the C of the Ph group and the C of the –CH_2_OH, –CH_2_O^–^, –CHO^–^, and –CHO groups.

b Cu···C = 3.353 Å.

**Figure 3:**
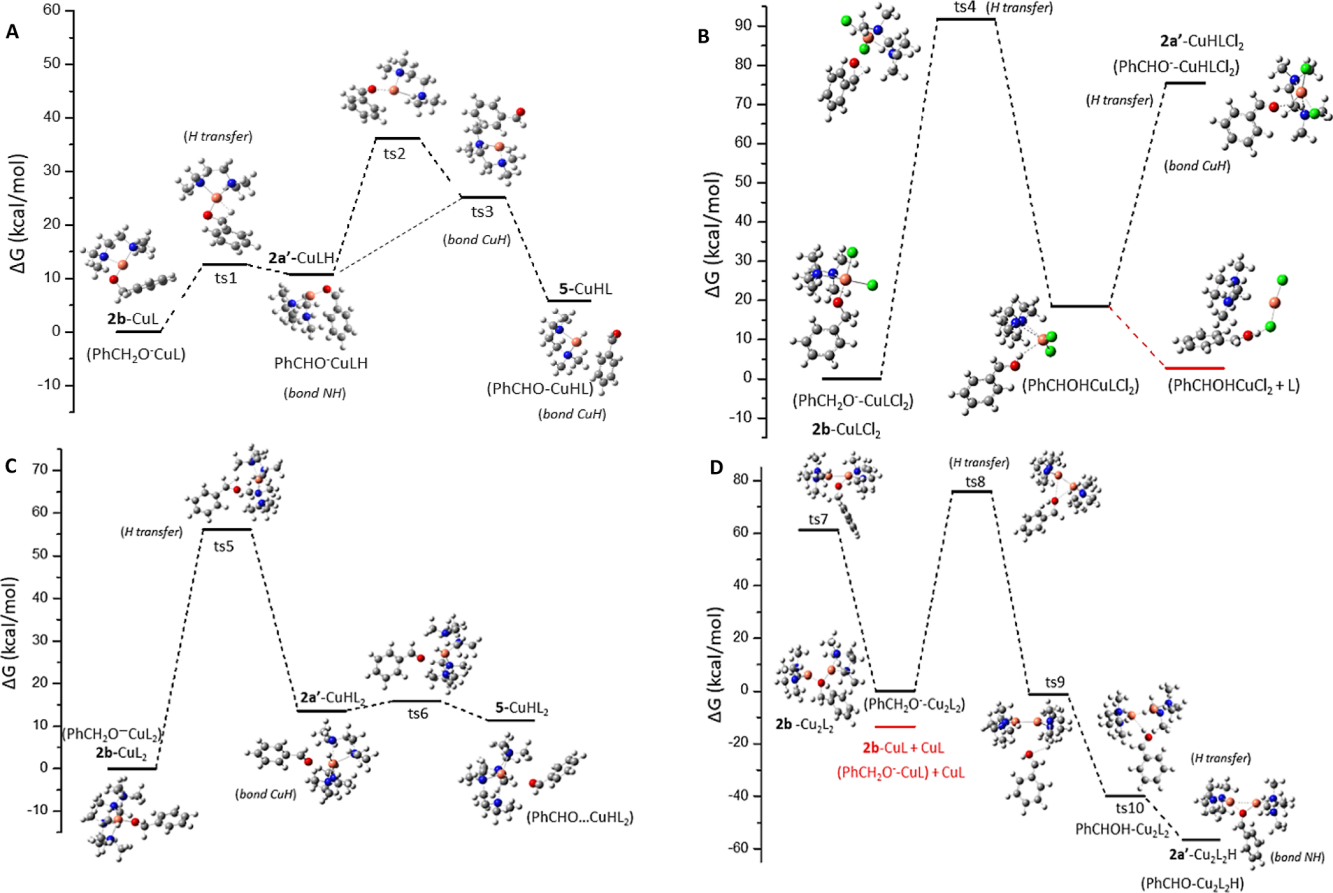
Reaction path A for the formation of benzaldehyde 5 *via* the (A) Cu^II^L catalytic system, *i.e.*, [CuL]^2+^; (B) CuLCl_2_ catalytic system; (C) Cu^II^L_2_ catalytic system, *i.e.*, [CuL_2_]^2+^; and (D) Cu_2_^II^L_2_ catalytic system, *i.e.*, [Cu_2_L_2_]^4+^.

Four reaction pathways were investigated theoretically using the four catalytic systems. The most energetically favorable one is the Cu^II^L (or [CuL]^2+^) (Figure 3A), where the energy barriers are less than 25 kcal/mol. A H atom of the benzyl alcohol methylene group –CH_2_– is transferred to the catalytic system *via* ts1. The free reaction energy demand is 12.2 kcal/mol, the H atom is attached to a N atom of the L, and **2a′** is formed. Then, the H atom is attached to the Cu atom, and benzaldehyde **5** is released. The formation reactions of **5***via* the Cu^II^LCl_2_, Cu^II^L_2_, and Cu_2_^II^L_2_ complexes are also shown in Figure 3B–D. The energy demands *via* Cu^II^LCl_2_ are very high, and the complex decomposes—see the formation of the PhCHOHClCuCl complex (Figure 3B). Furthermore, the use of Cu^II^L_2_ (or [CuL_2_]^2+^) as a catalyst has a higher energy demand, at about 55 kcal/mol, than in the case of Cu^II^L due to the steric effect of Cu^II^L_2_. The H is attached to Cu and **2a′**, and finally **5** is formed (Figure 3C).

Then, the Cu_2_^II^L_2_ complex was calculated as the catalyst. The energy demands are also high, and as **2a′** is formed, the Cu–Cu breaks (Figure 3D), and the **2b** anion is linked at both Cu centers. The Cu–Cu bond distance in Cu_2_L_2_ (Figure S2, Supporting Information) is 2.112 Å, while that in **2b**–Cu_2_L_2_ is 2.725 Å.

The reaction **2b**–Cu_2_L_2_ → **2b**–CuL + CuL is exothermic by −13.7 kcal/mol, and, therefore, **5** can be formed *via* the CuL catalyst (Figure 3A). It should be noted that the formation of **2a′**–Cu_2_L_2_ is exothermic by −56.6 kcal/mol with respect to **2b**–Cu_2_L_2_ and −42.9 kcal/mol with respect to **2b**–CuL + CuL because the interaction of both CuL groups further stabilizes the **2a′** anion. The Δ*G* for the formation of the **2a′** catalyst *via* Cu^II^L, Cu^II^L_2_, and Cu^II^LCl_2_ is 10.4, 13.6, and 18.4 kcal/mol, respectively.

**Figure 4:**
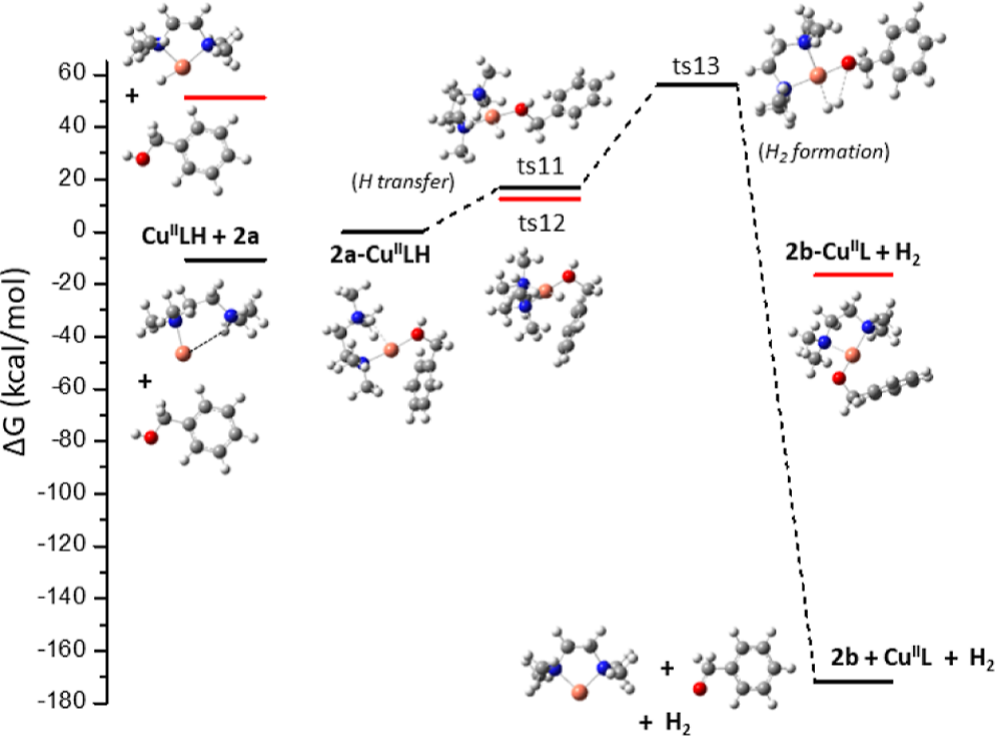
Last step of the catalytic cycle A for the formation of **5***via* the Cu^II^L catalytic system and catalyst regeneration.

Finally, Δ*G* of the formation of the **5** catalyst *via* Cu^II^L and Cu^II^L_2_ is 5.3 and 6.1 kcal/mol, respectively. The last step of the catalytic cycle, for the formation of benzaldehyde **5**, corresponds to regeneration of the catalyst (Figure 4). Benzyl alcohol **2a** forms a complex with **Cu**^**II**^**LH**, where the hydrogen is attached to N, *i.e.*, **2a**–Cu^II^LH, and then the H is transferred to the metal center. Two transition states, ts11 and ts12, are formed. The hydride H^–^ is attached to Cu, and a proton H^+^ from **2a** generates a H_2_ molecule—see ts13; finally, the H_2_ molecule is released. The Cu^II^LH–**2a** → Cu^II^L + **2b** + H_2_ reaction is exergonic, with a Δ*G* of −16.4 kcal/mol. Overall, the catalytic cycle A corresponds to the reaction **2a** → H_2_ + **5**, which is slightly endergonic by 6.2 kcal/mol regardless of the catalytic system; however, the copper catalyst has a key role in the hydrogen atom abstraction and transfer toward the formation of benzaldehyde **5** and the liberation of H_2_.

**Figure 5:**
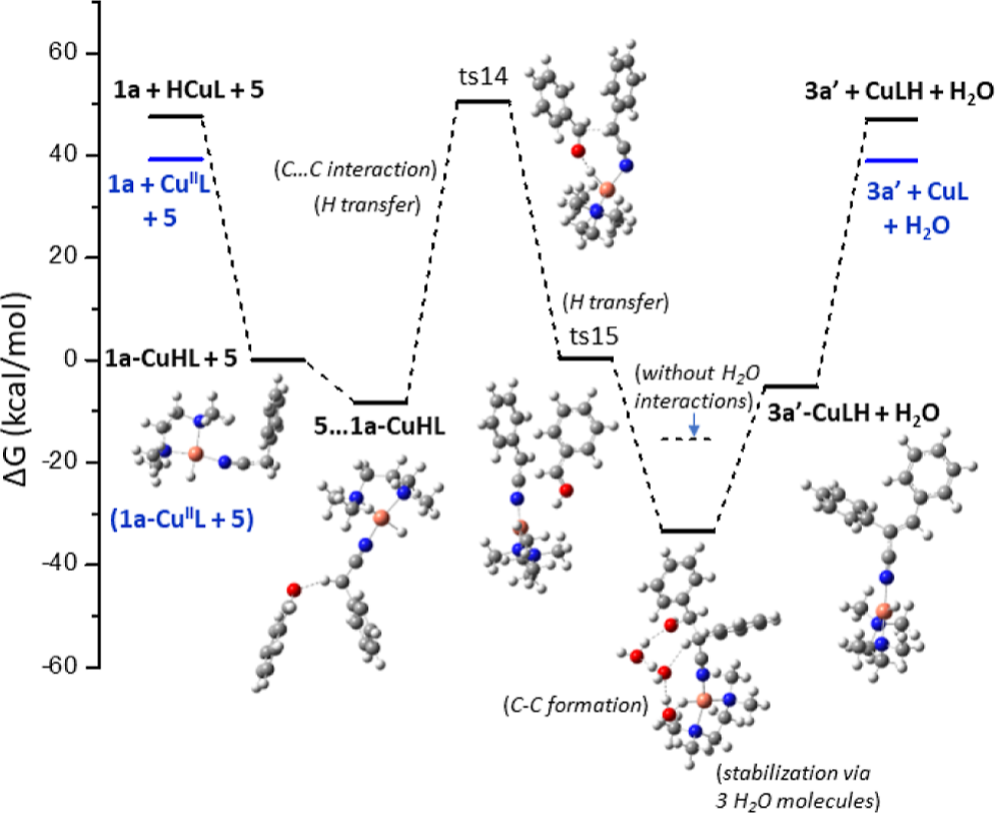
Reaction path for the formation of **3a′***via* the CuL and CuHL catalytic systems (at zero energy, **1a**–CuL + **5** and **1a**–CuHL + **5** have been located).

**Figure 6:**
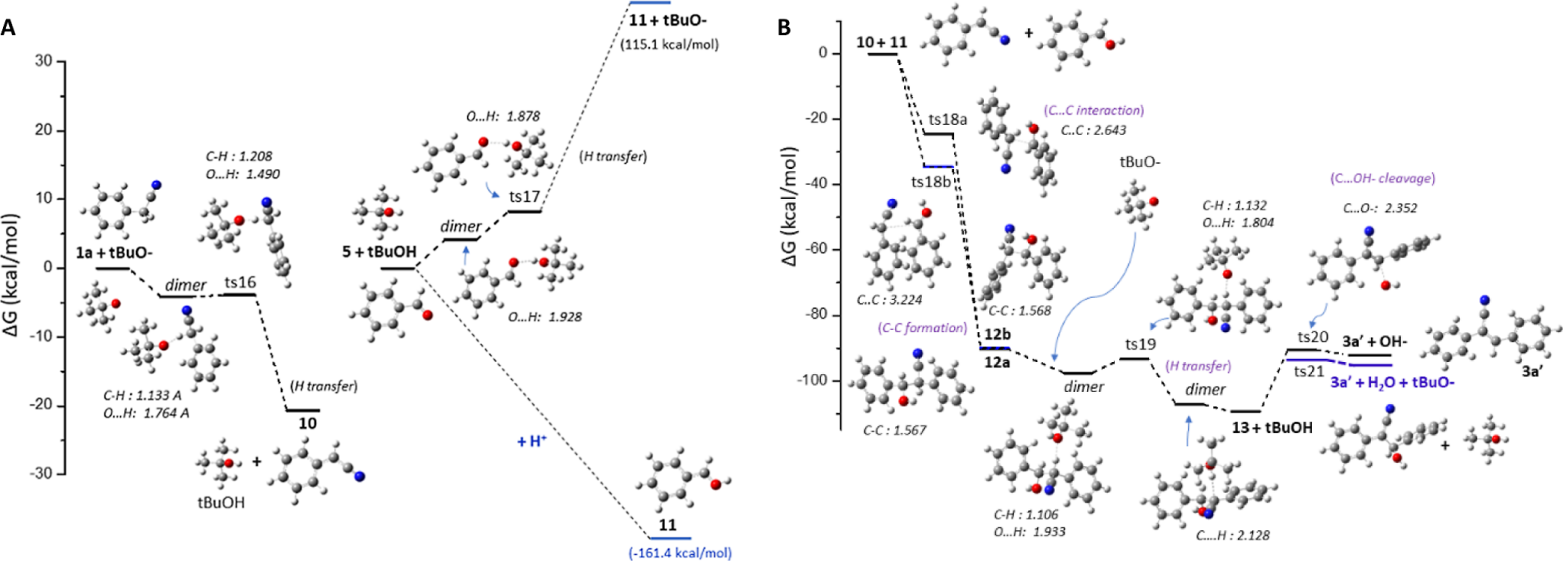
(A) Reaction path for the formation of **3a′** in the presence of a base, (A) formation of **10** [PhCHCN]^−^ and **11** [PhCHOH]^+^, and (B) condensation between **10** and **11**.

In the proposed mechanism of Scheme 6, in the catalytic cycle B, **3a′** is formed *via* the reaction **5** + **1a** → H_2_O + **3a′**, which is slightly exergonic by a Δ*G* of −0.41 kcal/mol (Table 3). The condensation of benzaldehyde and phenylacetonitrile can lead to **3a′**, either with the use of a Cu catalyst (Figure 5) or through a Knoevenagel condensation reaction, producing the α,β-unsaturated nitrile **3a′** (Figure 6). In the case of the Cu catalyst, phenylacetonitrile **1a** is attached to the catalyst, leading to **1a–CuL** and **1a–CuHL** complexes.

Then, benzaldehyde **5** interacts with the complex, where a triangle is formed between **5**, **1a**, and **CuHL**; see ts14. The hydride atom of the cupric center stabilizes the formation of the ts14 transition state. It should be noted that water molecules assist in the stabilization of the –OH group from **ts15**, resulting in the **3a′–CuLH** complex. There are significant energy demands for the formation of ts14; however, the formation of **1a–CuHL** provides the necessary energy. The Cu–N bond distance in the formed complexes between Cu and N of the CN group ranges from 1.85 to 1.95 Å, while the C≡N bond distance ranges from 1.147 to 1.188 Å.

Also note that in phenylacetonitrile **1a**, the C≡N bond distance is 1.152 Å, showing that the triple bond is retained in the formed complexes.

The reaction path for the synthesis of **3a′** through the condensation of benzaldehyde **5** and phenylacetonitrile **1a** is depicted in Figure 6. At first, a dimer between **1a** and *t*-BuO^–^ is formed, where a hydrogen atom from the benzyl alcohol methylene group –CH_2_ interacts with *t*-BuO^–^, with a stabilization energy Δ*G* of −4.1 kcal/mol. The hydrogen transfer is achieved *via* transition state ts16, with a very low energy barrier of 0.2 kcal/mol leading to the formation of anion **10** [PhCHCN]^−^; the reaction **1a** + *t*-BuO^–^ → **10** + *t*-BuOH is exergonic, with a reaction energy of −20.7 kcal/mol (Figure 6B). The oxygen atom of benzaldehyde **5** can be protonated, resulting in **11** [PhCHOH]^+^. The proton transfer from *t*-BuOH to **5** is endergonic; however, the use of *t*-BuO^–^ for the formation of the [PhCHCN]^−^ assists the reaction of **5** + *t*-BuOH → **11** + *t*-BuO^–^.

The Gibbs reaction energy of the formation is endergonic, but the reaction enthalpy is slightly exothermic. Then, the anion **10** and cation **11** can interact and be condensed, forming a C–C bond resulting in **12***via* the ts18a and ts18b structures, depending on their position. The ts18b structure is more stable than ts18a because of the π–π interaction of the two Ph groups. This interaction is responsible for the C–C bond distance of ts18b of 3.224 Å, which corresponds to the bond distance of π–π interactions.^74^

On the other hand, the C–C bond distance of ts18a is 2.643 Å. The reaction energy **10** + **11** → **12** is significantly exothermic with a reaction energy of Δ*G* = −90.3 kcal/mol. Compound **12**, which has a nitrile and a hydroxyl group, can form a dimer with *t*-BuO^–^, where a H···O bond is formed (Figure 6B).

The dimer has an interaction energy of −7.3 kcal/mol, and the H is transferred from **12** to *t*-BuO^–^*via* the ts19 structure, which corresponds to a small energy barrier of 4.2 kcal/mol. The anion **13** [PhCH(OH)C(CN)Ph]^−^ is formed; its formation reaction is exergonic, *i.e.*, **12** + *t*-BuO^–^ → **13** + *t*-BuOH with Δ*G* = −19.2 kcal/mol. Finally, the OH^–^ of **13** is easily cleaved *via* ts20, which corresponds to an energy barrier of 18.9 kcal/mol, while the removal *via* the assistance of the formation of dimer with the *t*-BuOH lowers the energy barrier by 3.1 kcal/mol. Both reactions **13** → **3a′** + HO^–^ with Δ*G* = 17.6 kcal/mol and **13** + *t*-BuOH → **3a′** + H_2_O + *t*-BuO^–^ with Δ*G* = 14.6 kcal/mol are endergonic but with small energy demands.

**Figure 7:**
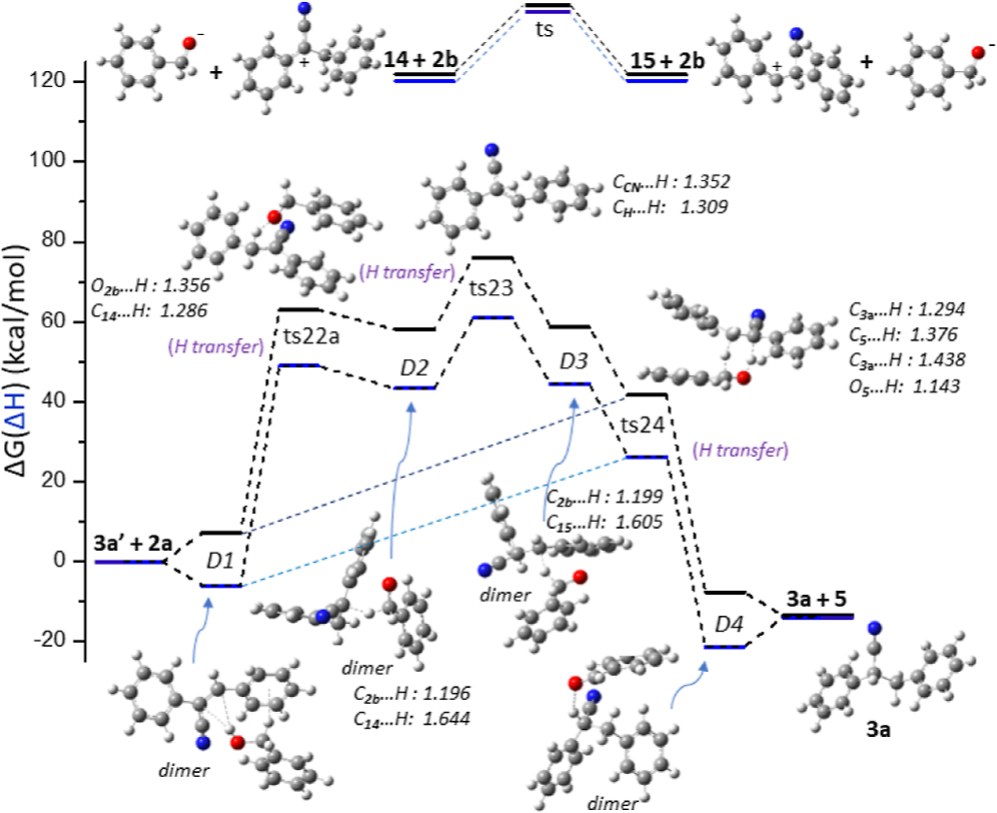
Reaction path for the formation of **3a** from the α,β-unsaturated nitrile **3a′** in the presence of a base; both Δ*G* Gibbs free energy (black line) and Δ*H* enthalpy (blue line) are plotted.

Along these lines, DFT calculations confirm that **3a′** can be formed *via* the Knoevenagel condensation of **5** and **1a** in the presence of a base without requiring the use of a metal catalyst. These results are in full agreement with the experimental observation of the excellent yield of **3a′** in the presence of a base alone (Scheme 5C). Finally, according to reaction cycle C, **3a** is formed through the reaction **3a′** + **2a** → **5** + **3a**, which is exergonic by a Δ*G*(Δ*H*) of −13.5(−13.9) kcal/mol (Figure 7).

Initially, a dimer is formed between **3a′** and **2a**. A variety of dimers, where the hydroxyl proton of **2a** interacts with the –CN group or the C atoms of the C=C group, are formed. The >C=C< ↔ >C^+^–C^–^< can receive the hydroxyl proton of **2a** or *t*-BuOH, resulting in cations **14** or **15**. The energy barrier for isomerization reaction **14** ↔ **15** is Δ*G* = 17.9 kcal/mol. Their formation reactions *via***2a** or *t*-BuOH are endergonic by 122 kcal/mol, while their formation reaction *via* the addition of the dissolved proton is exergonic by −148 kcal/mol (Table S7). The hydroxyl proton of **2a** or of *t*-BuOH is transferred to the C^–^ atom of the >C=C< ↔ >C^+^–C^–^< group, and the reaction barrier is about 50 kcal/mol.

However, the interaction of another R–OH molecule with the dimer **3a′**···**2a** or **3a′**···*t*-BuOH stabilizes the proton transfer by about 10 kcal/mol. Both cations **14** and **15** interact with **2b** (PhCH_2_O^–^), forming dimers D2 and D3, where a C–H bonds with the H atom of the CH_2_ group of **2b** (Figure 7).

Then, the O^–^ can form a double bond with the C of **2b**, while a hydrogen of its CH_2_ is transferred, saturating the nitrile and resulting in **3a**. Transition state ts24 has two hydrogen bonds between **3a′** and **2a**; the corresponding C–H and O–H bond distances range from 1.143 to 1.438 Å (Figure 7). This structure has one imaginary frequency that corresponds to the transfer of both H from **2a** to **3a′**; thus, the reaction **3a′** + **2a** → **5** + **3a** can also occur *via* ts24 that has a reaction barrier of Δ*G*(Δ*H*) = 41.6(26.3) kcal/mol.

To sum up, DFT calculations fully support the viability of the catalytic pathway proposed for the formation of the aldehyde from the corresponding alcohol, shedding light on the whole reaction mechanism. A variety of different Cu^II^ catalytic complexes were used, and the preferred reaction pathway has energy barriers of up to 24 kcal/mol.

The homolytic cleavage of the C(sp^3^)–H bond of the benzyl alcohol is favored energetically, and the release of H_2_ is likely to occur. Furthermore, the condensation of benzaldehyde and phenylacetonitrile can lead to **3a′**, either *via* the use of a Cu catalyst or *via* a condensation reaction in the presence of a base alone.

Finally, the unsaturated intermediate nitrile **3a′** is reduced to the corresponding saturated nitrile **3a**, with benzyl alcohol **2a** playing an important role in this step.

## Conclusions

We report the first copper-based catalytic system for the α-alkylation of aryl acetonitriles with benzyl alcohols, taking place through a C(sp^3^)–H hydrogen atom abstraction on the alcohol substrate. This sustainable, user-friendly, and low-cost catalytic protocol enables the formation of the desired nitriles in up to 99% yield, with the use of low catalyst and base loadings. A series of mechanistic and control experiments provide crucial information about the mechanism and the intermediates of the studied reaction. DFT calculations provide further insights, confirming the proposed unprecedented reaction mode for this transformation.

## Experimental Section

### General Catalytic Procedure under Ar

On a Schlenk-line, under an Ar atmosphere, a flame-dried (3×) J. Young tube was charged with anhydrous CuCl_2_ (5 mol %), *t*-BuOK (30 mol %), and a solution of TMEDA (5 mol %) in toluene (1 mL) and stirred for 5 min until solids were partially dissolved. Then, the alcohol (1 mmol) and nitrile (0.5 mmol) were added, and the reaction mixture was heated at 140 °C for 24 h in a sealed tube in a preheated oil bath. After cooling to room temperature, ethyl acetate was added, and the reaction mixture was filtered through a short plug of silica gel. The solvent was removed under vacuum, and the resulting residue was purified by column chromatography on silica gel using a mixture of petroleum ether/ethyl acetate as an eluent system to afford the desired nitriles. The same experimental procedure was followed for the reactions performed under air in a J. Young tube, except the use of the Schlenk-line.

### Computational Details

The geometries of the minima, intermediates, and transition states involved in the synthetic procedures were fully energetically optimized by DFT calculations [wB97XD^75^/6-311G+(d,p)^76^. The transition states (ts) were calculated employing the STQN method for locating transition structures.^77^ The wB97XD functional, which uses a version of Grimme’s D2 dispersion model, is regarded as an appropriate functional since dispersion forces exist in some transition states. Furthermore, its effectiveness in the calculation of catalytic reactions and weak interactions has already been checked.^74,75,78^ For all minima structures and transition states, their frequencies were calculated to confirm that they are true minima and transition states, respectively. The solvent has been included as a dielectric constant, employing the polarizable continuum model,^79^ which has been proven to reproduce solvent effects well.^80,81^ All calculations were carried out using the Gaussian 16 program.^82^

## Data Availability

The data underlying this study are available in this published article and its Supporting Information.

## Supporting Information Available

Experimental procedures, additional optimization experiments, computational information, compound characterization data, and copies of spectra (PDF)
