# Supplementary material for: Copper-Catalyzed α-Alkylation of Aryl Acetonitriles with Benzyl Alcohols
Source: J Org Chem. 2024 Sep 18;89(19):14242–54. doi: 10.1021/acs.joc.4c01662 (PMC11459520; doi:10.1021/acs.joc.4c01662)
Supplement: Supplementary file 1 — jo4c01662_si_001.pdf [file jo4c01662_si_001.pdf]

Electronic Supplementary Information (ESI)

Copper-catalyzed *α*-alkylation of aryl acetonitriles with benzyl alcohols

Marianna Danopoulou,[a] Leandros P. Zorba,[a] Athanasia P. Karantoni,[b] Demeter Tzeli,[b,c] and Georgios C. Vougioukalakis*[a]

[a] Laboratory of Organic Chemistry, National and Kapodistrian University of Athens, Panepistimiopolis, Athens 15771, Greece. Email: vougiouk@chem.uoa.gr
[b] Laboratory of Physical Chemistry, National and Kapodistrian University of Athens, Panepistimiopolis, Athens 15771, Greece.
[c] Theoretical and Physical Chemistry Institute, National Hellenic Research Foundation, Vas. Constantinou, 48, 11635, Athens, Greece

Table of Contents

1. General considerations S2
2. General procedure for catalytic reactions S2
2.1. Optimization of the reaction conditions S4
3. Kinetic profile S7
4. Derivatization of the cyanide moiety – Synthetic applications S8
4.1. Transformation of the cyanide group to 1H-tetrazole S8
4.2. Transformation of the cyanide group to Boc-protected amine S9
4.3. Transformation of the cyanide group to an amide S10
5. Experimental mechanistic studies S11
5.1. Radical traping experiment using $BrCCl_3$ S11
5.2. Radical quenching using TEMPO S11
6. Characterization of products S12
7. Copies of ^1H, $^{13}C\{^1H\}$ and $^{19}F\{^1H\}$ NMR spectra S19
8. Computational Details S47
9. References S96

1. General considerations

All reactions were performed under an Ar atmosphere following standard Schlenk techniques unless otherwise noted. All chemicals and reagents used for the experiments were purchased from Sigma-Aldrich, Fluorochem, Across Organics, Alfa-Aesar, or Thermo-Fisher Scientific and were used without further purification, besides benzaldehyde, which was purified by distillation prior to use. Ligands **L2** and **L4** were prepared according to literature procedures.[1,2] Thin-layer chromatography (TLC) was performed on Merck® silica gel 60 F_{254} plates with the layer thickness of 0.25 mm. Flash column chromatography was performed on Merck silica gel 60 (230 – 400 mesh). ^{1}H, ^{13}C-{^{1}H} and ^{19}F-{^{1}H} nuclear magnetic resonance (NMR) spectra were recorded on a Bruker® Avance 400 MHz Ultrashield or a Bruker® Avance 500 MHz Ultrashield instrument operating at 298 K, using the residual solvent peak as reference ($CDCl_3$: δ_H = 7.26 ppm, δ_C = 77.16 ppm, DMSO-d_6: δ_H = 2.50 ppm, δ_C = 39.52 ppm). Chemical shifts δ are given in p.p.m. and ^{1}H NMR peaks are assigned as: s (singlet), bs (broad singlet), d (doublet), dd (doublet of doublets), t (triplet), td (triplet of doublets), q (quartet), qd (quartet of doublets) and m (multiplet). GC–MS analysis was carried out on a SHIMADZU GCMS–QP2010 Plus with a DB–5 column. HRMS spectra were recorded on Bruker® Maxis Impact QTOF spectrometer.

2. General procedure for catalytic reactions

Catalytic protocol for under Ar atmosphere reactions. On a Schlenk-line under an Ar atmosphere, a flame dried (x3) J. Young tube was charged with anhydrous $CuCl_2$ (5 mol%), *t*-BuOK (30 mol%), a solution of TMEDA (5 mol%) in toluene (1 mL) and stirred for 5 minutes until solids are partially dissolved. The alcohol (1 mmol) and the nitrile (0.5 mmol) were then added and the reaction mixture was heated at 140 ^{o}C for 24 h in the sealed tube in a preheated oil bath. After cooling to room temperature, ethyl acetate was added and the reaction mixture was filtered through a short plug of silica. The solvent was removed under vacuo, internal standard (mesitylene / 1,3,5-trimethoxy benzene, 0.5 mmol) was added, and the mixture was analyzed by ^{1}H NMR to calculate the yield of the reaction. The solution was concentrated under vacuo and the

crude residue was purified by column chromatography on silica gel using a mixture of petroleum ether/ethyl acetate (or hexanes/diethyl ether) as eluent system to afford the desired product.

Catalytic protocol for under air atmosphere reactions. A J. Young tube was charged with anhydrous CuCl2 (5 mol%), t-BuOK (30 mol%), a solution of TMEDA (5 mol%) in toluene (1 mL) and stirred for 5 minutes until solids were partially dissolved. Then, the alcohol (1 mmol) and nitrile (0.5 mmol) were added, and the reaction mixture was heated at 140 ^{o}C for 24 h, in the sealed tube, in a preheated oil bath. After cooling to room temperature, ethyl acetate was added, and the reaction mixture was filtered through a short plug of silica gel. The solvent was removed under vacuo, internal standard (mesitylene / 1,3,5-trimethoxy benzene, 0.5 mmol) was added, and the mixture was analyzed by ^{1}H NMR to calculate the yield of the reaction. The solution was concentrated under vacuo and the crude residue was purified by column chromatography on silica gel using a mixture of petroleum ether/ethyl acetate (or hexanes/diethyl ether) as eluent system to afford the desired product.

2.1. Optimization of the reaction conditions

Table S1. Screening of the catalytic system[a]

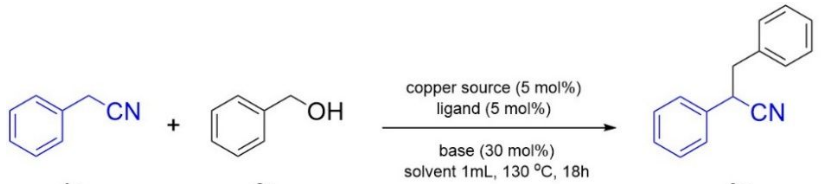

1a 2a 3a

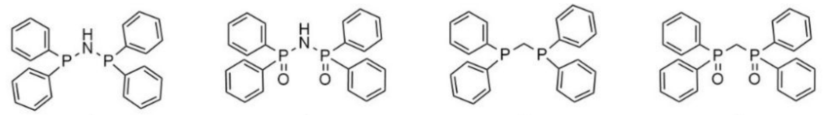

1 2 3 4

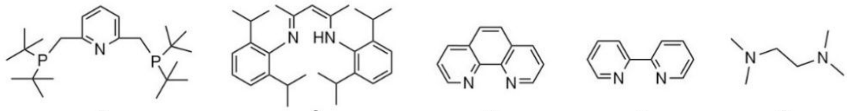

5 6 7 8 9

Entry	Copper source (5mol%)	Ligand (5mol%)	Base (30mol%)	Yield of 3a[b]
1	$Cu(OAc)_2$	-	*t*-BuOK	7%
2	$Cu(acac)_2$	-	*t*-BuOK	15%
3	$CuCl_{2\ anhydrous}$	-	*t*-BuOK	14%
4	CuCl	-	*t*-BuOK	11%
5	$Cu(OAc)_2$	L1	*t*-BuOK	0
6	CuCl	L1	*t*-BuOK	38%
7	$CuCl_{2\ anhydrous}$	L2	*t*-BuOK	0
8	$Cu(OAc)_2$	L3	*t*-BuOK	traces
9	CuCl	L3	*t*-BuOK	51%
10	$Cu(OAc)_2$	L4	*t*-BuOK	30%
11	CuCl	L5	*t*-BuOK	60%
12	$Cu(OAc)_2$	L6	*t*-BuOK	15%
13	CuCl	L7	*t*-BuOK	13%
14	$Cu(OTf)_2$	L8	*t*-BuOK	14%
15	$CuCl_2 \cdot 2H_2O$	L9	*t*-BuOK	60%
**16**	**$CuCl_{2\ anhydrous}$**	**L9**	***t*-BuOK**	**76%**

17[c]	$CuCl_{2\ anhydrous}$	L9	*t*-BuOK	70%
18	-	L9	*t*-BuOK	14%
19	-	-	*t*-BuOK	20%
20	-	-	-	0

Reaction conditions: [a] Phenylacetonitrile (0.5 mmol), benzyl alcohol (1 mmol), copper-source (5 mol%), ligand (5 mol%), *t*-BuOK (30 mol%), toluene (1 mL) in a J. Young tube at 130 ^{o}C for 18 h under Ar. [b] Yield was calculated using mesitylene (0.5 mmol) as internal standard. [c] Reaction was performed in a J. Young pressure tube in open-air.

Table S2. Temperature screening[a]

Entry	Temperature (^{o}C)	Yield[b]
1	120	5%
2	130	76%
3	140	90%
4	**140**	**96%**[c]
5	140	80%[c,d]

Reaction conditions: [a] Benzyl alcohol (1 mmol), phenylacetonitrile (0.5 mmol), $CuCl_2$ anhydrous (5 mol%), TMEDA (5 mol%), *t*-BuOK (30 mol%), toluene (1 mL) in a J. Young tube for 18 h under Ar. [b] Yield was calculated using mesitylene (0.5 mmol) as internal standard. [c] Reaction time 24 h. [d] Reaction was performed in a J. Young pressure tube in open-air.

Table S3. Catalyst loading screening[a]

Entry	$CuCl_2$ (x mol%)	TMEDA (x mol%)	Yield[b]
1	3	3	31%
**2**	**5**	**5**	**96%**

Reaction conditions: [a] Benzyl alcohol (1 mmol), phenylacetonitrile (0.5 mmol), $CuCl_2$ anhydrous (x mol%), TMEDA (x mol%), *t*-BuOK (30 mol%), toluene (1 mL) in a J.Young tube at 140 ^{o}C for 24 h under Ar. [b] Yield was calculated using mesitylene (0.5 mmol) as internal standard.

Table S4. Solvent screening[a]

Entry	Solvent	Yield[b]
**1**	**toluene**	**96%**
2	p-cymene	0
3	n-octane	58%
4	DMF	traces
5	1,4-dioxane	16%

Reaction conditions: [a] Benzyl alcohol (1 mmol), phenylacetonitrile (0.5 mmol), $CuCl_2$ anhydrous (5 mol%), TMEDA (5 mol%), *t*-BuOK (30 mol%), solvent (1 mL) in a J.Young tube at 140 °C for 24 h under Ar. [b] Yield was calculated using mesitylene (0.5 mmol) as internal standard.

Table S5. Base screening[a]

Entry	Base	Yield[b]
**1**	***t*-BuOK**	**96%**
2	*t*-BuONa	51%
3	KOH	72%
4	K_2CO_3	0
5	Cs_2CO_3	6%
6	DBU	0

Reaction conditions: [a] Benzyl alcohol (1 mmol), phenylacetonitrile (0.5 mmol), $CuCl_2$ anhydrous (5 mol%), TMEDA (5 mol%), base (30 mol%), toluene (1 mL) in a J. Young tube at 140 °C for 24 h under Ar. [b] Yield was calculated using mesitylene (0.5 mmol) as internal standard.

Table S6. Base-loading screening[a]

Entry	***t*-BuOK (x mol%)**	Yield[b]
1	10	0
2	20	24%
**3**	**30**	**96%**
4	-	0

Reaction conditions: [a] Benzyl alcohol (1 mmol), phenylacetonitrile (0.5 mmol), $CuCl_2$ anhydrous (5 mol%), TMEDA (5 mol%), *t*-BuOK (x mol%), toluene (1 mL) in a J.Young tube at 140 °C for 24 h under Ar. [b] Yield was calculated using mesitylene (0.5 mmol) as internal standard.

3. Kinetic profile

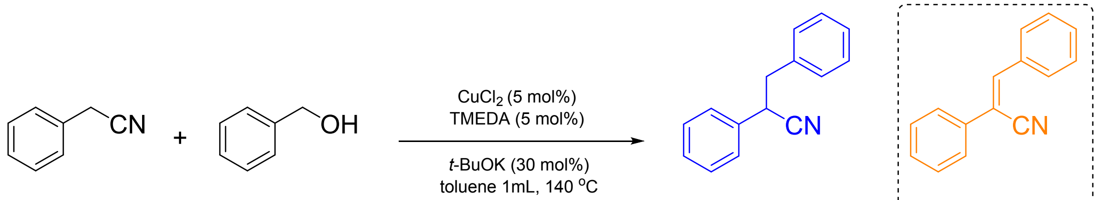

1a **2a** **3a** **3a'**

On a Schlenk-line under an Ar atmosphere, a flame dried (x3) J. Young tube was charged with phenylacetonitrile (0.5 mmol), benzyl alcohol (1 mmol), *t*-BuOK (30 mol%), $CuCl_2$ (5 mol%), TMEDA (5 mol%), toluene (1 mL), and 1,3,5-trimethoxybenzene (0.5 mmol). The reaction mixture was immersed in a preheated oil-bath at 140 ^{o}C. A small aliquot was retrieved every 1 h and analyzed by ^{1}H NMR, after removal of the solvent under reduced pressure. Two identical reactions were performed in order to identify the kinetic profile of the transformation. The first one was measured from time 0 to 12 h and the second from 14 h until completion (24 h).

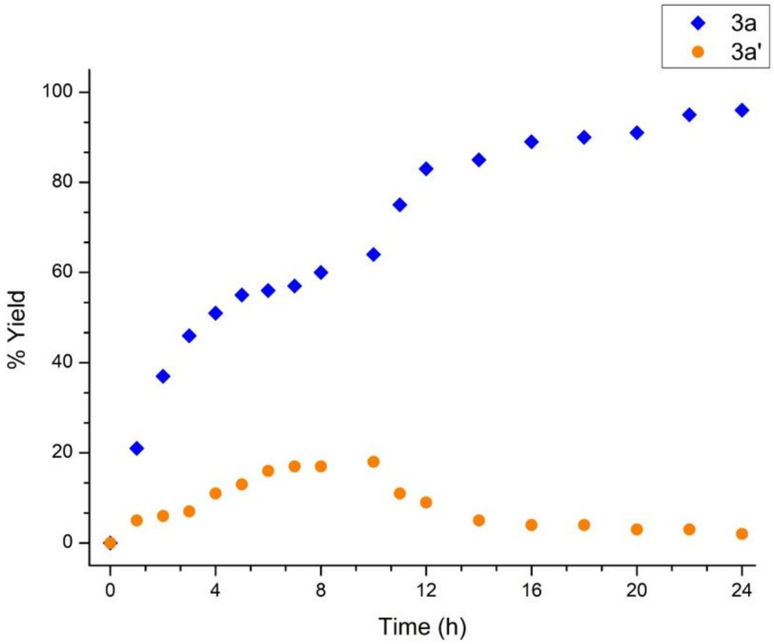

Figure S1. Kinetic profile of the synthesis of **3a** and **3a'**.

4. Derivatization of the cyanide moiety – Synthetic applications

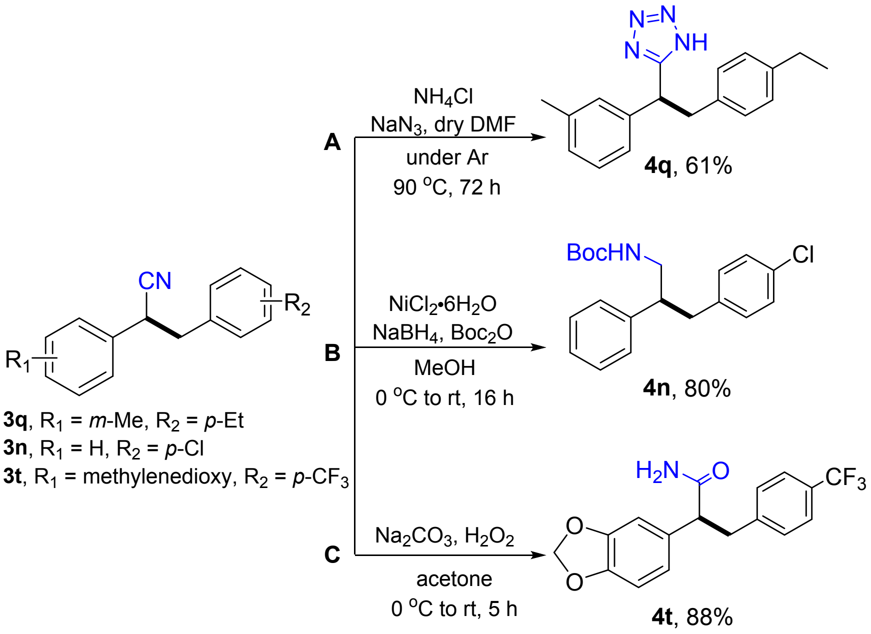

4.1. Transformation of the cyanide group to 1H-tetrazole

5-(2-(4-ethylphenyl)-1-(*m*-tolyl)ethyl)-1H-tetrazole (4q): In a flame dried J. Young tube under an Ar atmosphere, **3q** (0.2 mmol, 1 eq) was dissolved in dry DMF (1 mL). Then, NaN_3 (1 mmol, 5 eq) and NH_4Cl (1 mmol, 5 eq) were added and the reaction mixture was immersed in an oil-bath at 90 °C for 72 h in a sealed tube. The reaction was allowed to cool to room temperature, HCl 1M (50 mL) was added, and the mixture was stirred vigorously for a few minutes and then extracted with EtOAc (3 x 25 mL). The combined organic phases were washed with water (3 x 50 mL), then brine (50 mL), and dried over $MgSO_4$. The solvent was removed under vacuo and the resulting crude residue was subjected to column chromatography eluting with petroleum ether/ethyl acetate (starting from 90/10 to 0/100, broad elution of product starting from 80/20) to afford the pure product as a brown oil. Isolated yield: 61%, 36 mg.

^{1}H NMR (400 MHz, $CDCl_3$) δ 12.49 (bs, 1H, -NH), 7.19 (t, J = 7.7 Hz, 1H), 7.08 – 6.95 (m, 7H), 4.52 (t, J = 7.8 Hz, 1H), 3.65 (dd, J = 13.9, 8.0 Hz, 1H), 3.33 (dd, J = 13.9, 7.5 Hz, 1H), 2.56 (q, J = 7.7 Hz, 2H), 2.29 (s, 3H), 1.17 (td, J = 7.6, 1.7 Hz, 3H); **^{13}C{^{1}H} NMR** (100 MHz, $CDCl_3$) δ 159.0, 142.7, 138.97 (d, J = 7.92 Hz), 135.3, 129.0, 129.0, 128.8, 128.7, 128.0, 125.1, 43.8, 40.8, 28.5, 21.5, 15.6; **HRMS (**Q-TOF) *m/z* calculated for $C_{18}H_{20}N_4Na$ $[M+Na]^+$: 293.1761, found: 293.1749.

4.2. Transformation of the cyanide group to Boc-protected amine

tert-butyl (3-(4-chlorophenyl)-2-phenylpropyl)carbamate (4n) : A 25 mL round bottomed flask equipped with a stirring bar was charged with nitrile **3n** (0.25 mmol, 1 eq) dissolved in MeOH (5 mL). The solution was cooled with an ice-bath at 0 ^{o}C, where Boc_2O (0.5 mmol, 2 eq) and $NiCl_2$•$6H_2O$ (10 mol%) were added. After that, $NaBH_4$ (1.75 mmol, 7 eq) was added dropwise to the solution, resulting a color change from pale-yellow to black, and the reaction mixture was allowed to stir at room temperature for 16 h. After the completion of this time, the reaction was quenched with H_2O and the solvent was removed under vacuo. The resulting mixture was extracted with EtOAc (30 mL) and $NaHCO_3$ (2 x 30 mL). The combined organic phases were washed with water, brine, and dried over Na_2SO_4. The solvent was removed under vacuo, and the resulting brownish oil was filtered through a short silica plug. The desired product was obtained as an orange oil, pure without any further purification. Isolated yield: 80%, 70 mg.

^{1}H NMR (400 MHz, $CDCl_3$) δ 7.29 (d, 2H J = 8.2 Hz), 7.23 – 7. 21 (m, 1H), 7.15 – 7.08 (m, 4H), 6.93 (d, J = 7.9 Hz, 2H), 4.35 (bs, 1H), 3.59 – 3.53 (m, 1H), 3.27 – 3.20 (m, 1H), 3.06 – 2.99 (m, 1H), 2.93 (dd, J = 13.7, 6.1 Hz, 1H), 2.82 (dd, J = 13.7, 8.6 Hz, 1H), 1.39 (s, 9H); **^{13}C{^{1}H} NMR** (100 MHz, $CDCl_3$) δ 155.9, 141.7, 138.1, 131.9, 130.4, 128.7, 128.4, 128.0, 127.0, 79.3, 47.9, 45.5, 39.9, 28.4; **HRMS** (Q-TOF) *m/z* calculated for $C_{20}H_{24}ClNO_2Na$ $[M+Na]^+$: 368.1388, found: 368.1404.

4.3. Transformation of the cyanide group to an amide

2-(benzo[*d*][1,3]dioxol-5-yl)-3-(4-(trifluoromethyl)phenyl)propanamide (4t): A 25 mL round bottomed flask equipped with a stirring bar was charged with nitrile **3t** (0.26 mmol, 1 eq) and Na_2CO_3 (1.3 mmol, 5 eq) dissolved in acetone (3 mL). The solution was cooled at 0 °C in an ice-bath, and H_2O_2 30% (w/w) (3 mL) was added. The reaction mixture was then allowed to stir at room temperature for 5 h. After the completion of that time, the reaction was quenched with H_2O and the solvent was removed under vacuo. The resulting mixture was extracted with EtOAc (30 mL). The combined organic phases were washed with water, brine, and dried over Na_2SO_4.The solvent was removed under vacuo and the resulting solid was filtered through a short silica plug. The desired product was obtained pure as a white solid, without the need of further purification by column chromatography. Isolated yield: 88%, 80 mg.

^{1}H NMR (400 MHz, DMSO-d_6) δ 7.60 (d, J = 7.8 Hz, 2H), 7.40 (d, J = 6.7 Hz, 3H, overlapping peaks of 2H aromatic and a -NH), 6.96 (s, 1H), 6.86 – 6.73 (m, 3H, overlapping peaks of 2H aromatic and a -NH), 5.97 (t, J = 2.5 Hz, 2H), 3.72 (dd, J = 9.1 and 6.3 Hz, 1H), 3.30 (dd, J = 13.9, 9.3 Hz, 1H), 2.92 (dd, J = 13.7, 6.3 Hz, 1H); **^{13}C{^{1}H} NMR** (100 MHz, DMSO-d_6) δ 173.8, 147.1, 146.0, 144.9, 144.9, 134.1, 129.6, 128.5, 127.2, 126.9, 126.6, 126.3, 125.8, 124.9, 124.9, 124.9, 124.8, 123.1, 120.9, 108.0, 107.9, 100.8, 51.9, 38.4; **^{19}F{^{1}H} NMR** (376 MHz, DMSO-d_6) δ -60.72; **HRMS** (Q-TOF) *m/z* calculated for $C_{17}H_{14}F_3NO_3Na$ $[M+Na]^+$: 360.0818, found: 360.0812.

5. Experimental mechanistic studies

5.1. Radical traping experiment using $BrCCl_3$

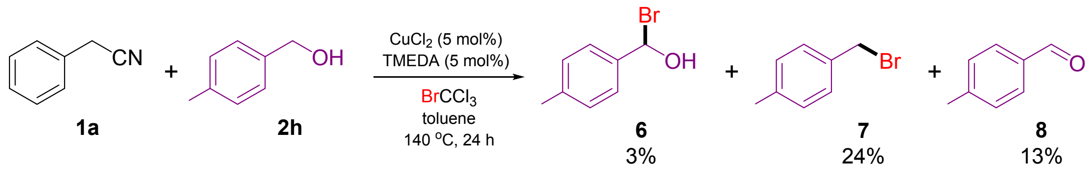

On a Schlenk-line under an Ar atmosphere, a flame dried (x3) J. Young tube was charged with anhydrous $CuCl_2$ (5 mol%), *t*-BuOK (30 mol%), a solution of TMEDA (5 mol%) in toluene (1 mL) and stirred for 5 minutes until solids were partially dissolved. Then, alcohol (1 mmol), nitrile (0.5 mmol) and $BrCCl_3$ (0.5 mmol) were added and the reaction mixture was heated at 140 °C for 24 h in the sealed tube in a preheated oil bath. After cooling to room temperature, the solvent was evaporated under vacuo and the crude reaction mixture was analyzed by ^{1}H-NMR and GC-MS. ^{1}H-NMR showed that bromo(p-tolyl)methanol **6** was obtained in 3% yield, along with the corresponding benzyl bromide **7**, which was obtained in 24% yield (**7** is formed from the decomposition of **6**). Benzaldehyde **8** was also obtained in 13% yield, as shown by ^{1}H-NMR. GC-MS confirmed the existence of **7** and **8**, but the halogenated alcohol **6** was not observed (most probably it decomposes to 7 in the capillary column).

5.2. Radical quenching using TEMPO

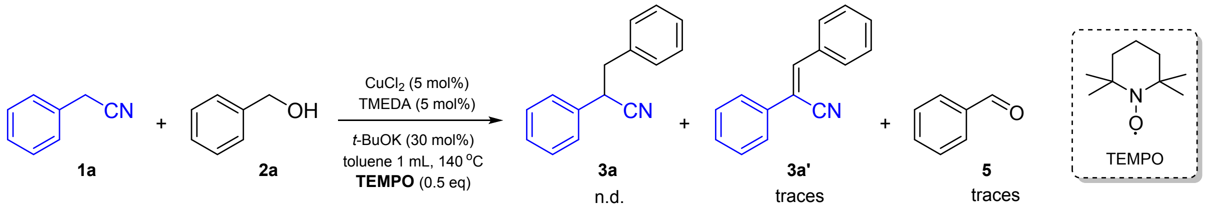

On a Schlenk-line under an Ar atmosphere, a flame dried (x3) J. Young tube was charged with anhydrous $CuCl_2$ (5 mol%), *t*-BuOK (30 mol%), a solution of TMEDA (5 mol%) in toluene (1 mL) and stirred for 5 minutes until solids were partially dissolved. The alcohol (1 mmol), the nitrile (0.5 mmol) and 2,2,6,6-tetramethylpiperidine 1-oxyl (TEMPO) (0.5 mmol) were then added and the reaction mixture was heated at 140 °C for 24 h in the sealed tube in a preheated oil bath. After cooling to room temperature, the solvent was evaporated under vacuo and the crude reaction mixture was analyzed by ^{1}H-NMR.

6. Characterization of products

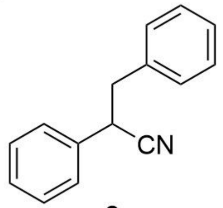

3a

2,3-diphenylpropanenitrile (3a)[3]: Obtained under the optimized reaction conditions. The compound was isolated as a colorless oil using flash column chromatography eluting with petroleum ether/ethyl acetate 97/3 (Yield using mesitylene as internal standard - IS: 96%, Isolated yield: 88%, 91 mg). **^{1}H NMR** (400 MHz, $CDCl_3$) δ 7.38 – 7.26 (m, 8H), 7.15 – 7.13 (m, 2H), 4.00 (dd, J = 8.3, 6.5 Hz, 1H), 3.23 – 3.11 (qd, J = 13.6, 7.4 Hz, 2H); **^{13}C{^{1}H} NMR** (100 MHz, $CDCl_3$) δ 136.3, 135.3, 129.3, 129.1, 128.7, 128.3, 127.5, 127.4, 120.4, 42.3, 39.9

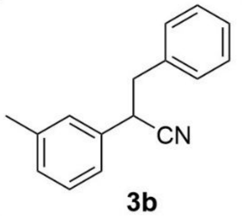

3b

3-phenyl-2-(*m*-tolyl)propanenitrile (3b)[4]: Obtained under the optimized reaction conditions. The compound was isolated as a colorless oil using flash column chromatography eluting with petroleum ether/ethyl acetate 97/3 (Yield using mesitylene as IS: 82%, Isolated yield: 71%, 79 mg). **^{1}H NMR** (400 MHz, $CDCl_3$) δ 7.33 – 7.22 (m, 4H), 7.17 – 7.13 (m, 3H), 7.13 (s, 1H), 7.06 (d, J = 7.8 Hz, 1H), 3.95 (dd, J = 8.5, 6.4 Hz, 1H), 3.20 – 3.09 (m, 2H), 2.35 (s, 3H); **^{13}C{^{1}H} NMR** (100 MHz, $CDCl_3$) δ 138.9, 136.5, 135.2, 129.2, 129.0, 128.9, 128.6, 128.2, 127.4, 124.5, 120.5, 42.3, 39.8, 21.4

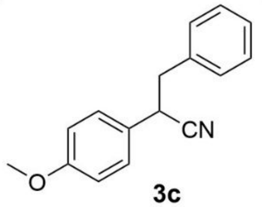

3c

2-(4-methoxyphenyl)-3-phenylpropanenitrile (3c)[3]: Obtained under the optimized reaction conditions. The compound was isolated as a white solid using flash column chromatography eluting with petroleum ether/ethyl acetate 95/5 (Yield using mesitylene as IS: 96%, Isolated yield: 80%, 95 mg). **^{1}H NMR** (400 MHz, $CDCl_3$) δ 7.30 – 7.26 (m, 3H), 7.14 (dd, J = 13.7, 7.2 Hz, 4H), 6.87 (d, J = 8.59 Hz, 2H), 3.95 (dd, J = 8.2, 6.6 Hz, 1H), 3.81 (s, 3H), 3.14 (qd, J = 13.6, 7.3 Hz, 2H); **^{13}C{^{1}H} NMR** (100 MHz, $CDCl_3$) δ 159.4, 136.4, 129.3, 128.7, 128.6, 127.3, 127.2, 120.7, 114.4, 55.4, 42.3, 39.0

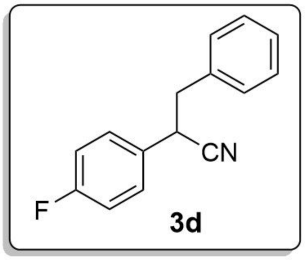

2-(4-fluorophenyl)-3-phenylpropanenitrile (3d)[3]: Obtained under the optimized reaction conditions with reaction time 36 h. The compound was isolated as a white solid using flash column chromatography eluting with petroleum ether/ethyl acetate 97/3 (Yield using mesitylene as IS: 63%, Isolated yield: 57%, 64 mg). **^{1}H NMR** (400 MHz, $CDCl_3$) δ 7.32 – 7.19 (m, 3H), 7.20 (dd, J = 8.5, 5.2 Hz, 2H), 7.11 – 7.08 (m, 2H), 7.04 (t, J = 8.5 Hz, 2H), 3.99 (t, J = 7.3 Hz, 1H), 3.24 – 3.06 (m, 2H); **^{13}C{^{1}H} NMR** (100 MHz, $CDCl_3$) δ 163.8, 161.3, 136.0, 131.0 (d, J = 3.5 Hz), 129.3 (d, J = 5.3 Hz), 129.3, 128.8, 127.6, 120.3, 116.2, 115.9, 42.3, 39.1; **^{19}F{^{1}H} NMR** (376 MHz, $CDCl_3$) δ -113.53

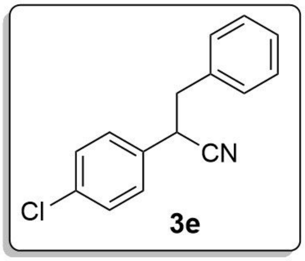

2-(4-chlorophenyl)-3-phenylpropanenitrile (3e)[3]: Obtained under the optimized reaction conditions. The compound was isolated as a white solid using flash column chromatography eluting with petroleum ether/ethyl acetate 97/3 (Yield using mesitylene as IS: 75%, Isolated yield: 73%, 88 mg). **^{1}H NMR** (400 MHz, $CDCl_3$) δ 7.34 – 7.28 (m, 5H), 7.18 – 7.16 (m, 2H), 7.12 – 7.10 (m, 2H), 3.99 (t, J = 7.3 Hz, 1H), 3.22 – 3.08 (m, 2H); **^{13}C{^{1}H} NMR** (100 MHz, $CDCl_3$) δ 135.9, 134.3, 133.7, 129.3, 129.3, 129.0, 128.8, 127.6, 120.0, 42.1, 39.2

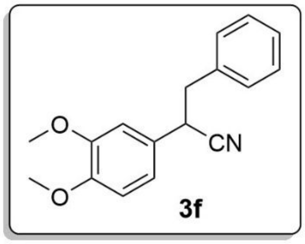

2-(3,4-dimethoxyphenyl)-3-phenylpropanenitrile (3f)[3]: Obtained under the optimized reaction conditions. The compound was isolated as a white solid using flash column chromatography eluting with petroleum ether/ethyl acetate 85/15 (Yield using mesitylene as IS: 61%, Isolated yield: 67%, 89 mg). **^{1}H NMR** (400 MHz, $CDCl_3$) δ 7.31 – 7.26 (m, 3H), 7.13 – 7.11 (d, J = 6.9 Hz, 2H), 6.84 – 6.79 (m, 2H), 6.64 (s, 1H), 3.97 – 3.93 (m, 1H), 3.88 (s, 3H), 3.80 (s, 3H), 3.22 – 3.08 (m, 2H); **^{13}C{^{1}H} NMR** (100 MHz, $CDCl_3$) δ 149.2, 149.0, 136.4, 129.4, 128.7, 127.6, 127.4, 120.7, 119.9, 111.4, 110.7, 56.0, 42.3, 39.4

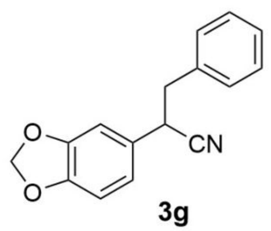

3g

2-(benzo[*d*][1,3]dioxol-5-yl)-3-phenylpropanenitrile (3g)[4]: Obtained under the optimized reaction conditions. The compound was isolated as a white solid using flash column chromatography eluting with petroleum ether/ethyl acetate 95/5 (Yield using 1,3,5-trimethoxybenzene as IS: 83%, Isolated yield: 81%, 102 mg). **^{1}H NMR** (400 MHz, $CDCl_3$) δ 7.32 – 7.28 (m, 3H), 7.14 (d, *J* = 7 Hz, 2H), 6.77 – 6.75 (m, 2H), 6.69 (d, *J* = 8.1 Hz, 1H), 5.98 (s, 2H), 3.90 (t, *J* = 7.4 Hz, 1H), 3.20 – 3.06 (qd, *J* = 13.5, 7.2 Hz, 2H); **^{13}C{^{1}H} NMR** (100 MHz, $CDCl_3$) δ 148.3, 147.6, 136.3, 129.3, 128.9, 128.7, 127.5, 121.1, 120.5, 108.6, 107.9, 101.5, 42.4, 39.5

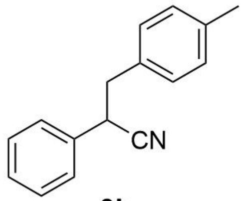

3h

2-phenyl-3-(*p*-tolyl)propanenitrile (3h)[5]: Obtained under the optimized reaction conditions. The compound was isolated as a colorless oil using flash column chromatography eluting with petroleum ether/diethyl ether 95/5 (Yield using mesitylene as IS: 89%, Isolated yield: 81%, 90 mg). **^{1}H NMR** (400 MHz, $CDCl_3$) δ 7.39 – 7.30 (m, 3H), 7.28 – 7.26 (m, 2H), 7.10 (d, *J* = 7.7 Hz, 1H), 7.03 (d, *J* = 7.7 Hz, 2H), 3.97 (dd, *J* = 8.4, 6.6 Hz, 1H), 3.18 – 3.07 (m, 1H), 2.33 (s, 3H); **^{13}C{^{1}H} NMR** (100 MHz, $CDCl_3$) δ 137.0, 135.4, 133.3, 129.3, 129.1, 129.0, 128.2, 127.5, 120.5, 41.8, 39.9, 21.1

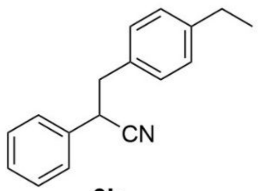

3k

3-(4-ethylphenyl)-2-phenylpropanenitrile (3k)[6]: Obtained under the optimized reaction conditions. The compound was isolated as a colorless oil using flash column chromatography eluting with petroleum ether/ethyl acetate 98/2 (Yield using mesitylene as IS: 81%, Isolated yield: 78%, 92 mg). **^{1}H NMR** (400 MHz, $CDCl_3$) δ 7.39 – 7.26 (m, 5H), 7.14 (d, *J* = 7.6 Hz, 2H), 7.08 (d, *J* = 7.9 Hz, 2H), 3.98 (dd, *J* = 8.4, 6.5 Hz, 1H), 3.19 – 3.08 (m, 2H), 2.63 (q, *J* = 7.6 Hz, 2H), 1.23 (t, *J* = 7.6 Hz, 3H); **^{13}C{^{1}H} NMR** (100 MHz, $CDCl_3$) δ 143.4, 135.4, 133.6, 129.2, 129.0, 128.2, 128.1, 127.5, 120.5, 41.9, 40.0, 28.5, 15.6

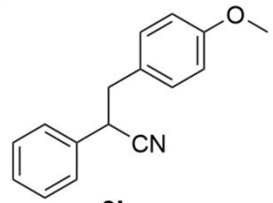

3l

3-(4-methoxyphenyl)-2-phenylpropanenitrile (3l)[3]: Obtained under the optimized reaction conditions. The compound was isolated as a yellowish oil using flash column chromatography eluting with petroleum ether/ethyl acetate 90/10 (Yield using mesitylene as IS: 85%, Isolated yield: 76%, 90 mg). **^{1}H NMR** (400 MHz, $CDCl_3$) δ 7.38 – 7.31 (m, 3H), 7.25 – 7.22 (m, 2H), 7.05 – 7.03 (m, 2H), 6.84 – 6.80 (m, 2H), 3.96 (dd, J = 8.1, 6.5 Hz, 1H), 3.79 (s, 3H), 3.18 – 3.04 (m, 2H); **^{13}C{^{1}H} NMR** (100 MHz, $CDCl_3$) δ 158.9, 135.3, 130.3, 129.0, 128.3, 128.2, 127.5, 120.5, 114.0, 55.2, 41.4, 40.1

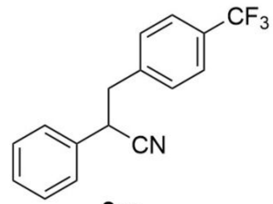

3m

2-phenyl-3-(4-(trifluoromethyl)phenyl)propanenitrile (3m)[3]: Obtained under the optimized reaction conditions. The compound was isolated as a white solid using flash column chromatography eluting with petroleum ether/ethyl acetate 97/3 (Yield using mesitylene as IS: 87%, Isolated yield: 79%, 108 mg). **^{1}H NMR** (400 MHz, $CDCl_3$) δ 7.55 (d, J = 8.01 Hz, 2H), 7.40 – 7.34 (m, 3H), 7.26 – 7.23 (m, 4H), 4.04 (dd, J = 7.9, 6.6 Hz, 1H), 3.28 – 3.18 (m, 2H); **^{13}C{^{1}H} NMR** (100 MHz, $CDCl_3$) δ 140.2 (d, J = 1.5 Hz), 134.6, 130.2, 129.9, 129.7, 129.6, 129.2, 129.2, 128.5, 128.2, 127.5, 125.6 (q, J = 3.8 Hz), 122.8, 120.0, 41.7, 39.3; **^{19}F{^{1}H} NMR** (376 MHz, $CDCl_3$) δ -62.46

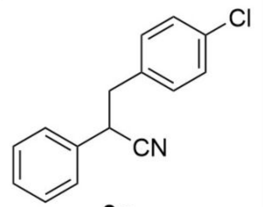

3n

3-(4-chlorophenyl)-2-phenylpropanenitrile (3n)[5]: Obtained under the optimized reaction conditions. The compound was isolated as a yellowish solid using flash column chromatography eluting with petroleum ether/diethyl ether 90/10 (Yield using mesitylene as IS: 77%, Isolated yield: 68%, 82 mg). **^{1}H NMR** (400 MHz, $CDCl_3$) δ 7. 40 – 7.31 (m, 3H), 7.27 – 7.23 (m, 4H), 7.04 (d, J = 7.9 Hz, 2H), 3.99 (t, J = 7.2 Hz, 1H), 3.19 – 3.09 (m, 2H); **^{13}C{^{1}H} NMR** (100 MHz, $CDCl_3$) δ 134.8, 134.6, 133.4, 130.7, 129.1, 128.8, 128.4, 127.5, 120.1, 41.4, 39.6

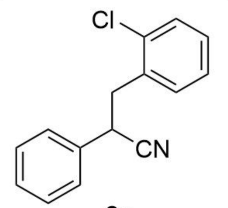

3o

3-(2-chlorophenyl)-2-phenylpropanenitrile (3o)[4]: Obtained under the optimized reaction conditions, by using 50 mol% of base. The compound was isolated as a white solid using flash column chromatography eluting with petroleum ether/ethyl acetate 95/5 (Yield using mesitylene as IS: 68%, Isolated yield: 63%, 76 mg). **^{1}H NMR** (400 MHz, $CDCl_3$) δ 7.41 – 7.33 (m, 6H), 7.26 – 7.21 (m, 3H), 4.18 (dd, J = 9.7, 6.1 Hz, 1H), 3.34 – 3,20 (m, 2H); **^{13}C{^{1}H} NMR** (100 MHz, $CDCl_3$) δ 135.3, 134.2, 134.1, 131.9, 129.8, 129.2, 129.2, 128.4, 127.3, 127.2, 120.2, 40.5, 37.7

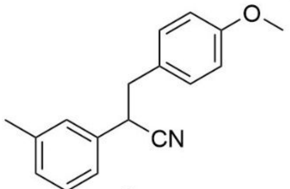

3p

3-(4-methoxyphenyl)-2-(*m*-tolyl)propanenitrile (3p): Obtained under the optimized reaction conditions, by using 50 mol% of base. The compound was isolated as a colorless oil using flash column chromatography eluting with petroleum ether/diethyl ether 90/10 (Yield using 1,3,5-trimethoxybenzene as IS: 81%, Isolated yield: 76%, 96 mg). **^{1}H NMR** (400 MHz, $CDCl_3$) δ 7.23 (d, J = 7.8 Hz, 1H), 7.14 (d, J = 7.6 Hz, 1H), 7.10 – 7.04 (m, 4H), 6.85 – 6.82 (m, 2H), 3.92 (t, J = 7.5 Hz, 1H), 3.80 (s, 3H), 3.14 – 3.04 (m, 2H), 2.36 (s, 3H); **^{13}C{^{1}H} NMR** (100 MHz, $CDCl_3$) δ 158.9, 138.9, 135.3, 130.3, 128.9 (d, J = 2.2 Hz), 128.6, 128.2, 124.6, 120.6, 114.0, 55.3, 41.5, 40.1, 21.4; **HRMS** (Q-TOF) *m/z* calculated for $C_{17}H_{17}NONa$ $[M+Na]^+$: 274.1202, found: 274.1202

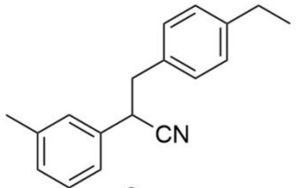

3q

3-(4-ethylphenyl)-2-(*m*-tolyl)propanenitrile (3q): Obtained under the optimized reaction conditions. The compound was isolated as an orange oil using flash column chromatography eluting with hexanes/diethyl ether 97/3 (Yield using mesitylene as IS: 86%, Isolated yield: 82%, 102 mg). **^{1}H NMR** (400 MHz, $CDCl_3$) δ 7.26 – 7.23 (m, 1H), 7.15 – 7.06 (m, 7H), 3.93 (t, J = 7.7 Hz, 1H), 3.16 – 3.06 (m, 2H), 2.63 (q, J = 7.8 Hz, 2H), 2.35 (s, 3H), 1.23 (td, J = 7.6, 2.2 Hz, 3H); **^{13}C{^{1}H} NMR** (100 MHz, $CDCl_3$) δ 143.4, 138.9, 135.4, 133.7, 129.2, 128.9, 128.1, 124.5, 120.6, 42.0, 40.0, 28.5, 21.4, 15.6; **HRMS** (Q-TOF) *m/z* calculated for $C_{18}H_{19}NNa$ $[M+Na]^+$: 272.1410, found: 272.1417

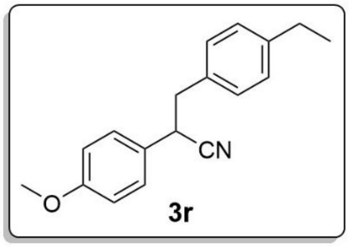

3-(4-ethylphenyl)-2-(4-methoxyphenyl)propanenitrile (3r): Obtained under the optimized reaction conditions. The compound was isolated a yellowish crystalline solid using flash column chromatography eluting with petroleum ether/diethyl ether 92/8 (Yield using mesitylene as IS: 80%, Isolated yield: 78%, 103 mg). **^{1}H NMR** (500 MHz, $CDCl_3$) δ 7.22 – 7.19 (m, 2H), 7.15 (d, J = 7.9 Hz, 2H), 7.09 (d, J = 7.8 Hz, 2H), 6.91 – 6.88 (m, 2H), 3.95 (dd, J = 8.5, 6.4 Hz, 1H), 3.82 (s, 3H), 3.17 – 3.06 (m, 2H), 2.65 (q, J = 7.6 Hz, 2H), 1.25 (t, J = 7.6 Hz, 3H); **^{13}C{^{1}H} NMR** (125 MHz, $CDCl_3$) δ 159.3, 143.3, 133.6, 129.2, 128.6, 128.1, 127.4, 120.8, 114.3, 55.3, 41.8, 39.1, 28.5, 15.6; **HRMS** (Q-TOF) *m/z* calculated for $C_{18}H_{19}NO_2Na$ $[M+Na]^+$: 288.1359, found: 288.1373

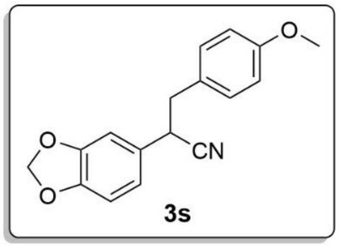

2-(benzo[*d*][1,3]dioxol-5-yl)-3-(4-methoxyphenyl)propanenitrile (3s): Obtained under the optimized reaction conditions. The compound was isolated as a yellow solid using flash column chromatography eluting with petroleum ether/ethyl acetate 80/20 (Yield using mesitylene as IS: 83%, Isolated yield: 71%, 100 mg). **^{1}H NMR** (400 MHz, $CDCl_3$) δ 7.06 – 7.03 (m, 2H), 6.85 – 6.81 (m, 2H), 6.77 – 6.74 (m, 2H), 6.68 (dd, J = 8.0, 1.9 Hz, 1H), 5.98 (s, 2H), 3.87 (dd, J = 5.7, 2.4 Hz, 1H), 3.79 (s, 3H), 3.07 (qd, J = 13.7, 7.3 Hz, 1H); **^{13}C{^{1}H} NMR** (100 MHz, $CDCl_3$) δ 158.9, 148.1, 147.5, 130.3, 128.9, 128.3, 121.0, 120.6, 114.0, 108.5, 107.9, 101.4, 55.2, 41.4, 39.7; **HRMS** (Q-TOF) *m/z* calculated for $C_{17}H_{15}NO_3Na$ $[M+Na]^+$: 304.0949, found: 304.0940

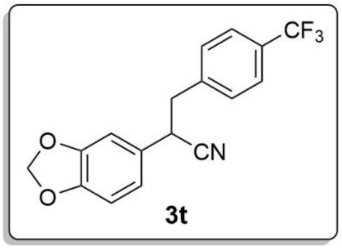

2-(benzo[*d*][1,3]dioxol-5-yl)-3-(4-(trifluoromethyl)phenyl) propanenitrile (3t): Obtained under the optimized reaction conditions. The compound was isolated an orange oil using flash column chromatography eluting with petroleum ether/ethyl acetate 90/10 (Yield using 1,3,5-trimethoxybenzene as IS: 99%, Isolated yield: 83%, 132 mg). **^{1}H NMR** (400 MHz, $CDCl_3$) δ 7.56 (d, J = 7.9 Hz, 2H), 7.24 (s, 2H), 6.77 (d, J = 8.5 Hz, 1H), 6.67 (d, J = 8.0 Hz, 1H), 6.00 (s, 2H), 3.94 (t, J = 7.3 Hz, 1H), 3.24 – 3.14 (m, 2H); **^{13}C{^{1}H} NMR** (100 MHz, $CDCl_3$) δ 148.4, 147.8, 140.2, 130.3, 129.9, 129.6, 129.3, 128.2, 128.2, 125.6 (q, J = 3.8 Hz), 125.5, 122.8, 121.1, 120.0, 108.7, 107.7, 101.6, 41.8, 39.0; **^{19}F{^{1}H}**

NMR (376 MHz, $CDCl_3$) δ -62.50 ; **HRMS** (Q-TOF) *m/z* calculated for $C_{17}H_{12}F_3NO_3Na$ $[M+Na]^+$: 342.0712, found: 342.0704

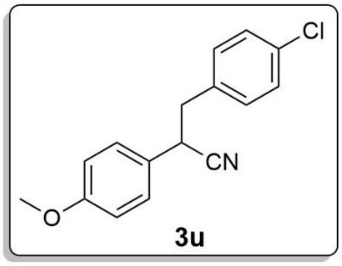

3-(4-chlorophenyl)-2-(4-methoxyphenyl)propanenitrile (3u): Obtained under the optimized reaction conditions, by using 50 mol% of base. The compound was isolated as a white solid using flash column chromatography eluting with petroleum ether/ethyl acetate 85/15 (Yield using mesitylene as IS: 77%, Isolated yield: 70%, 95 mg). **^{1}H NMR** (500 MHz, $CDCl_3$) δ 7.25 (d, *J* = 8.1 Hz, 2H), 7.15 – 7.12 (m, 2H), 7.03 (d, *J* = 8.1 Hz, 2H), 6.88 – 6.85 (m, 2H), 3.94 (t, *J* = 7.2 Hz, 1H), 3.80 (s, 3H), 3.10 (dd, *J* = 13.6, 7.2 Hz, 2H); **^{13}C{^{1}H} NMR** (125 MHz, $CDCl_3$) δ 159.5, 134.7, 133.3, 130.7, 128.8, 128.7, 126.7, 120.4, 114.4, 55.4, 41.5, 38.8; **HRMS** (Q-TOF) *m/z* calculated for $C_{16}H_{14}ClNONa$ $[M+Na]^+$: 294.0656, found: 294.0656

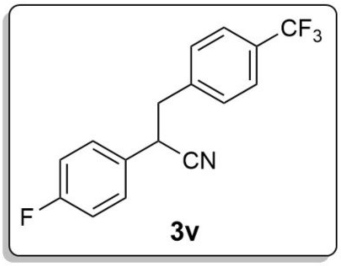

2-(4-fluorophenyl)-3-(4-(trifluoromethyl)phenyl)propanenitrile (3v): Obtained under the optimized reaction conditions, by using 50 mol% of base. The compound was isolated as a pale-yellow oil using flash column chromatography eluting with petroleum ether/ethyl acetate 92/8 (insufficient purification of the starting nitrile from the product since they appear having the exact same Rf under various solvent systems in TLC) Second column chromatography with toluene (TLC in toluene revealed two spots separated) (Yield using mesitylene as IS: 67%, Isolated yield: 62%, 90 mg). **^{1}H NMR** (400 MHz, $CDCl_3$) δ 7.57 – 7.55 (m, 2H), 7.23 – 7.19 (m, 4H), 7.09 – 7.04 (m, 2H), 4.03 (t, *J* = 7.2 Hz, 1H), 3.21 (td, *J* = 7.7, 7.0 and 2.2 Hz, 2H); **^{13}C{^{1}H} NMR** (100 MHz, $CDCl_3$) δ 163.9, 161.4, 139.9, 130.4, 130.4, 130.1, 129.8, 129.3, 129.3, 128.2, 125.8, 125.7, 125.7, 125.7, 125.4, 122.7, 120.0, 119.8, 116.4, 116.2, 41.8, 38.7; **^{19}F{^{1}H} NMR** (376 MHz, $CDCl_3$) δ -62.58, -112.95; **HRMS** (Q-TOF) *m/z* calculated for $C_{16}H_{11}F_4NNa$ $[M+Na]^+$: 316.0720, found: 316.0718

7. Copies of ^{1}H, $^{13}C\{^{1}H\}$ and $^{19}F\{^{1}H\}$ NMR spectra

^{1}H NMR (400 MHz, $CDCl_3$) of 3a:

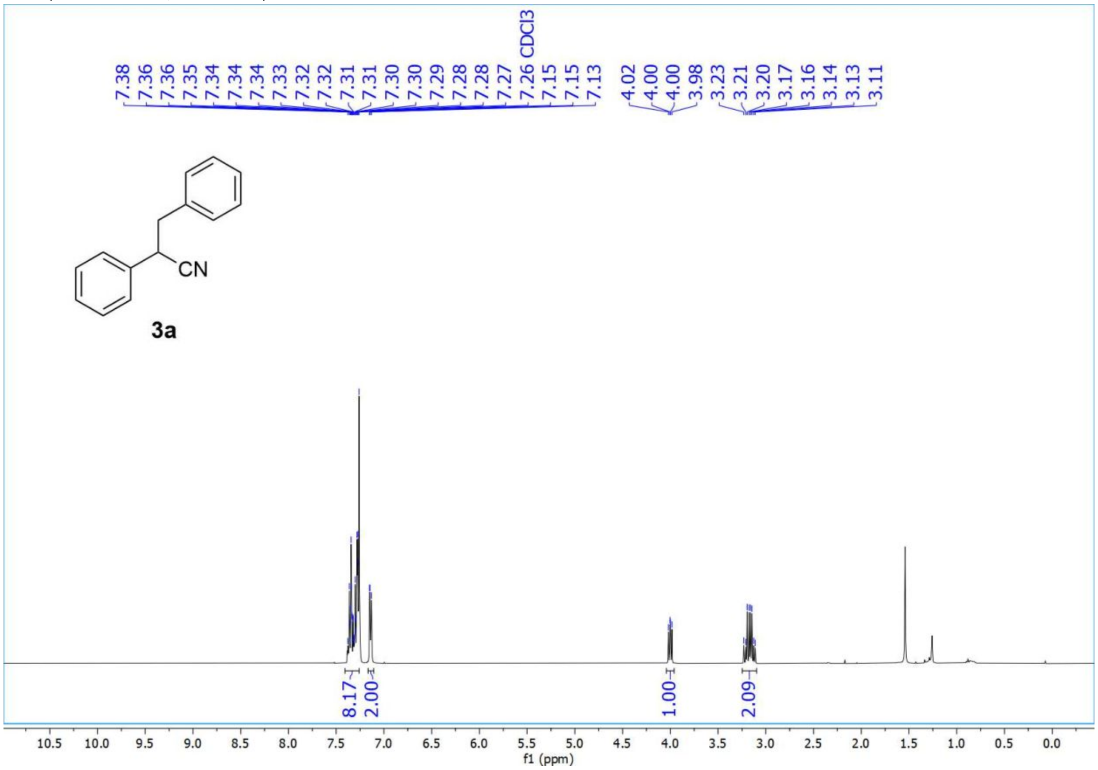

$^{13}C\{^{1}H\}$ NMR (100 MHz, $CDCl_3$) of 3a:

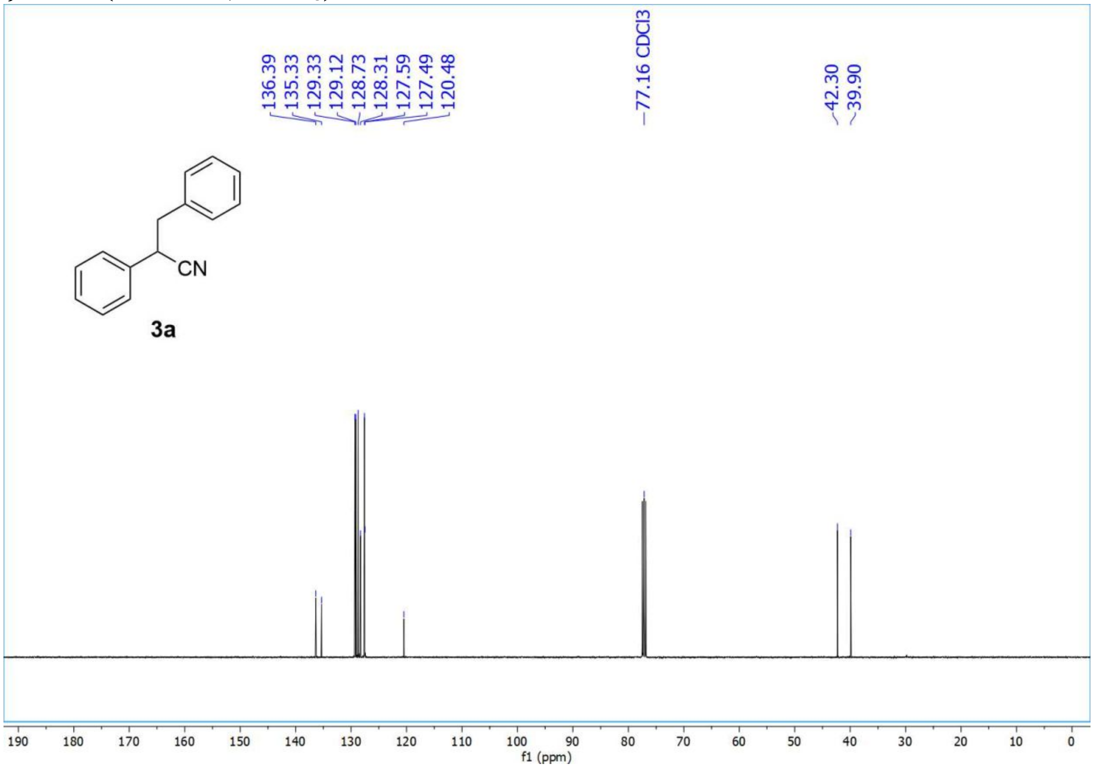

^{1}H NMR (400 MHz, $CDCl_3$) of 3b:

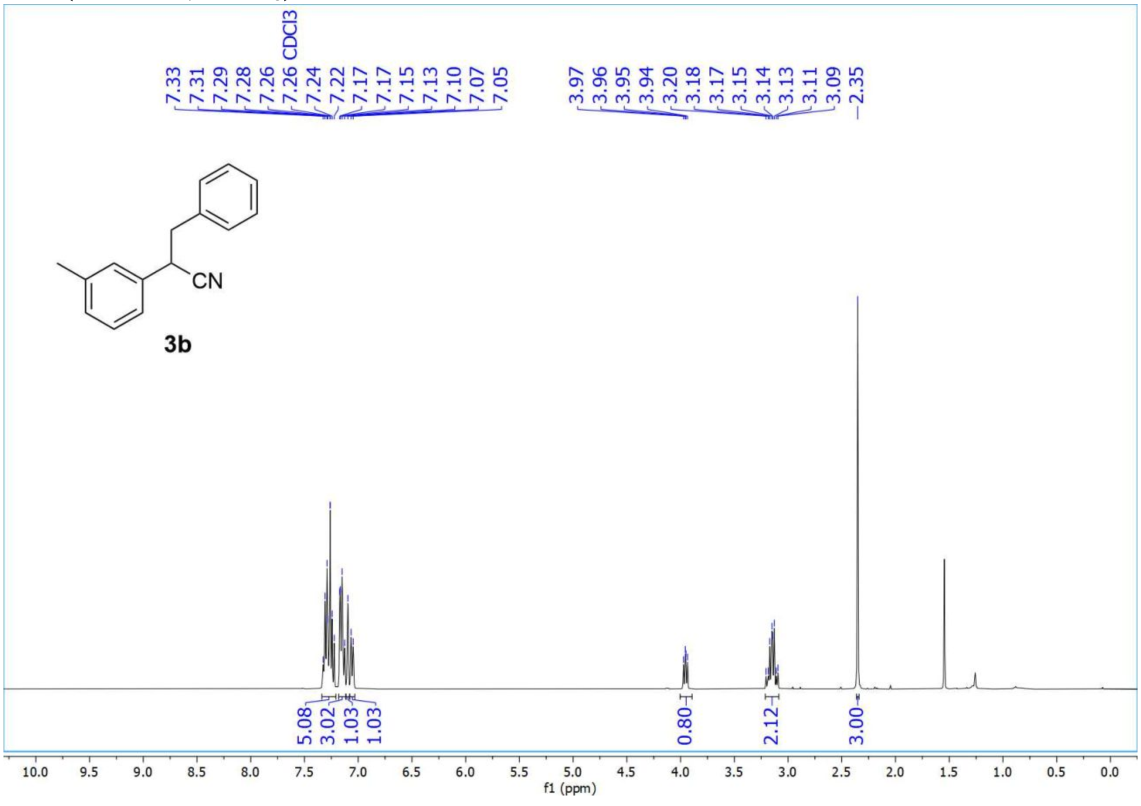

^{13}C{^{1}H} NMR (100 MHz, $CDCl_3$) of 3b:

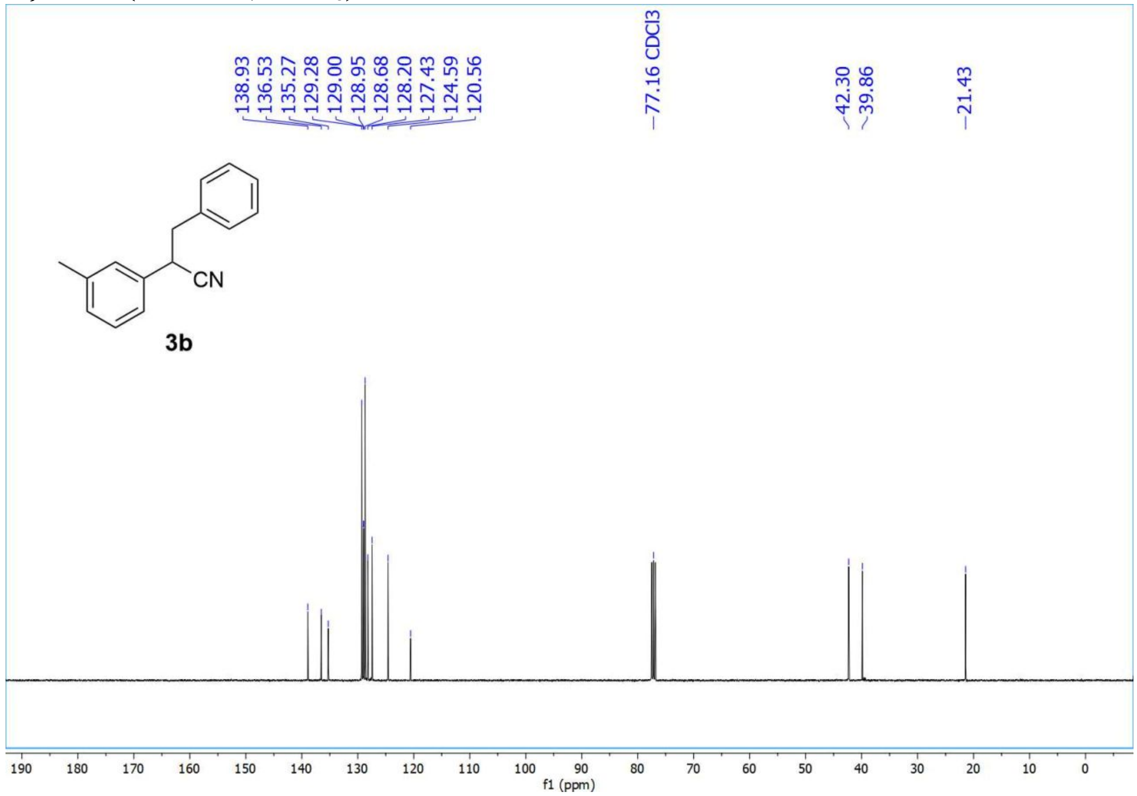

^{1}H NMR (400 MHz, $CDCl_3$) of 3c:

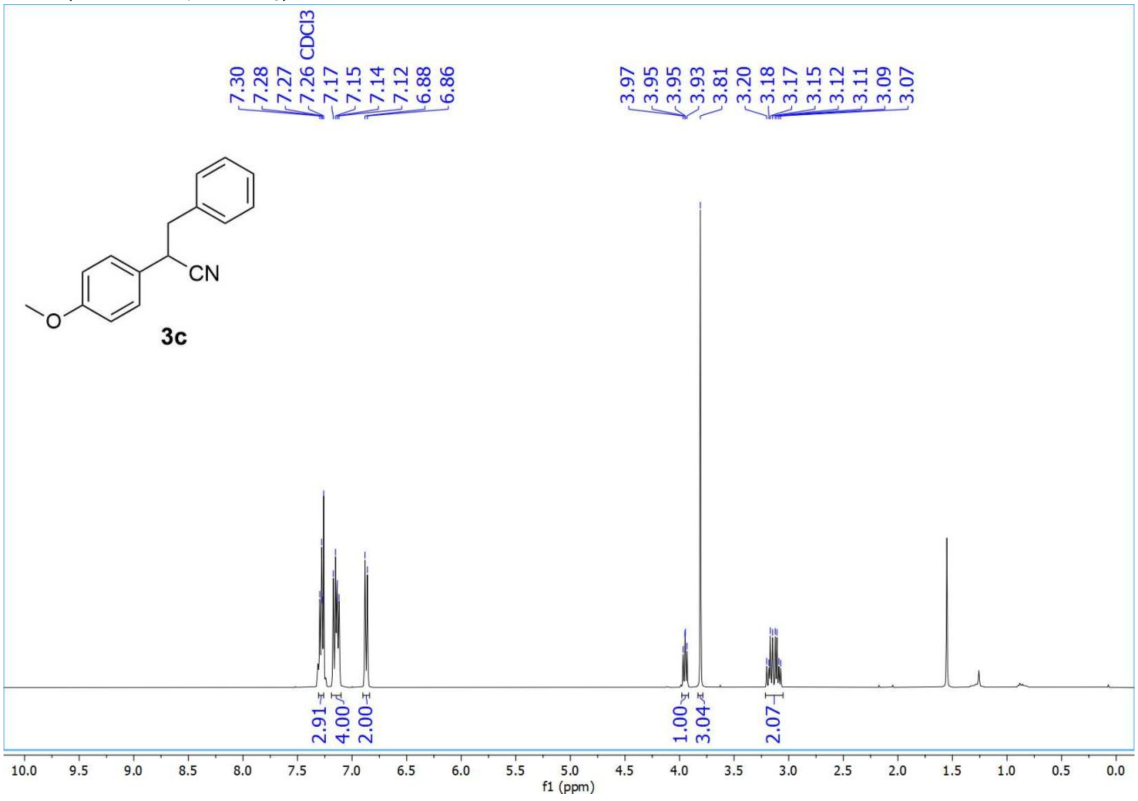

^{13}C{^{1}H} NMR (100 MHz, $CDCl_3$) of 3c:

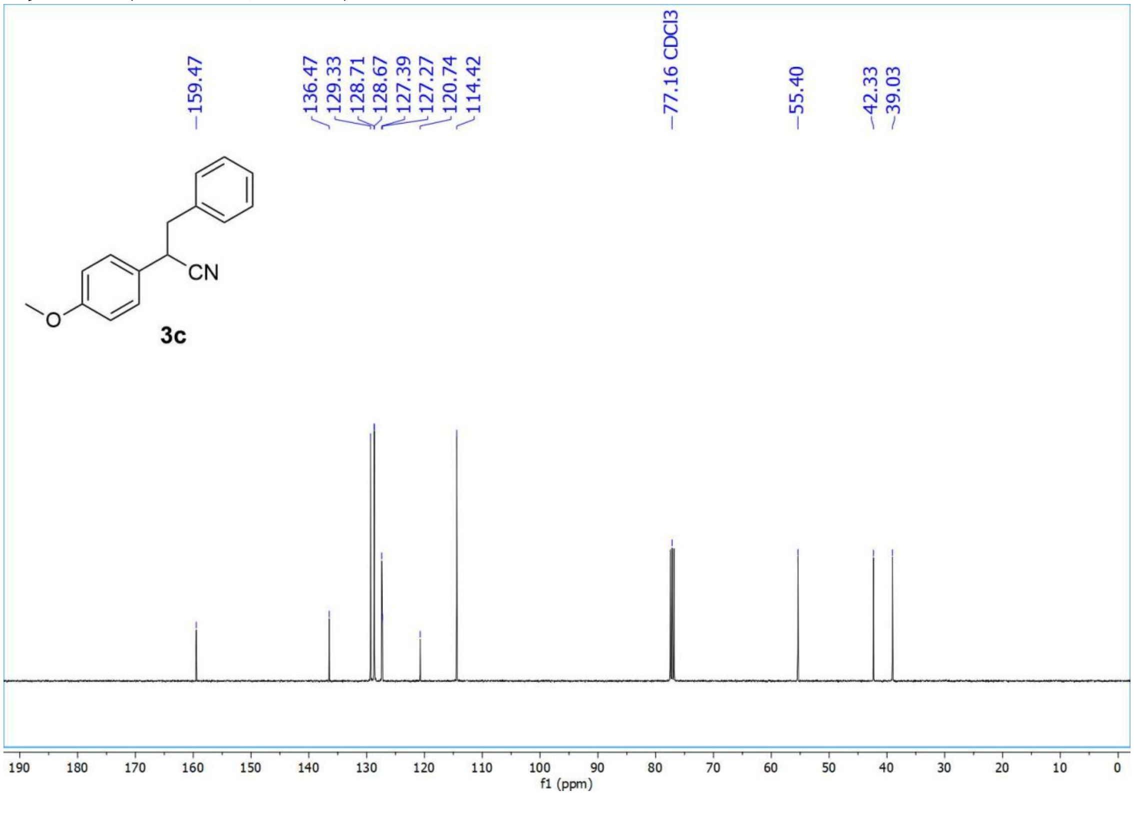

^{1}H NMR (400 MHz, $CDCl_3$) of 3d:

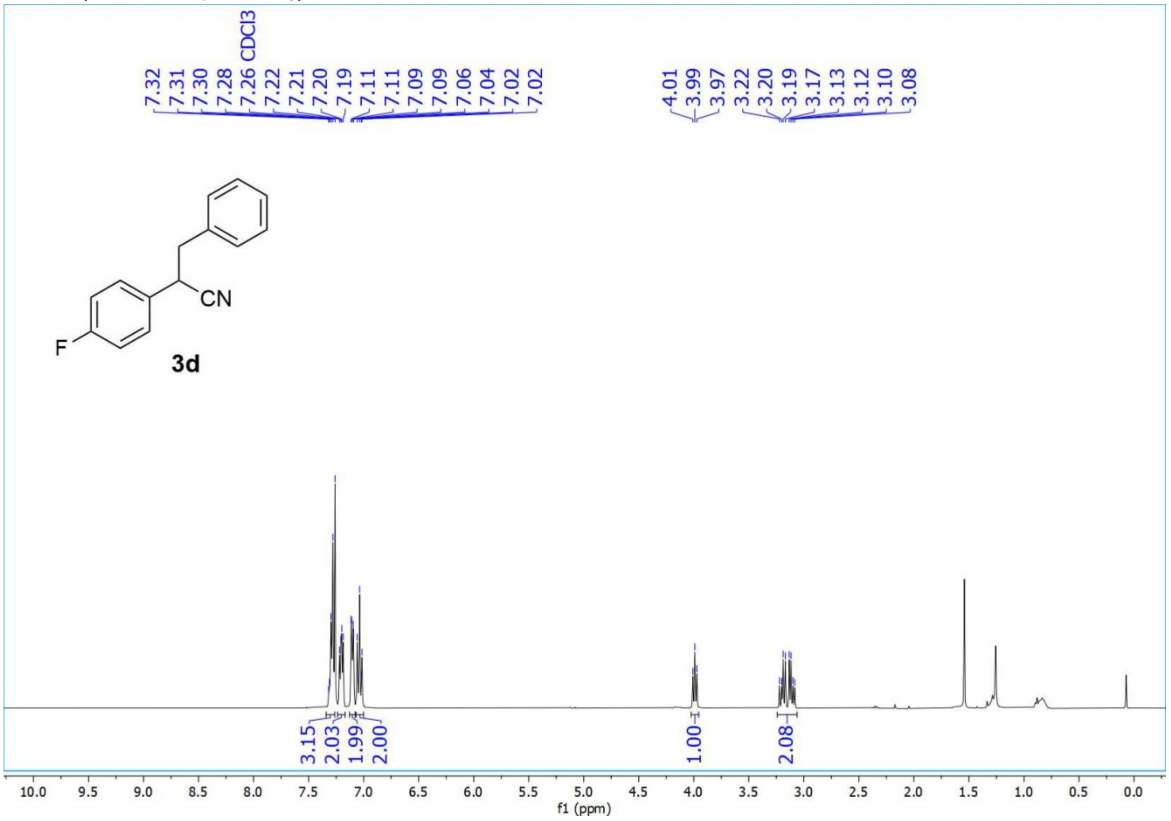

$^{13}C\{^{1}H\}$ NMR (100 MHz, $CDCl_3$) of 3d:

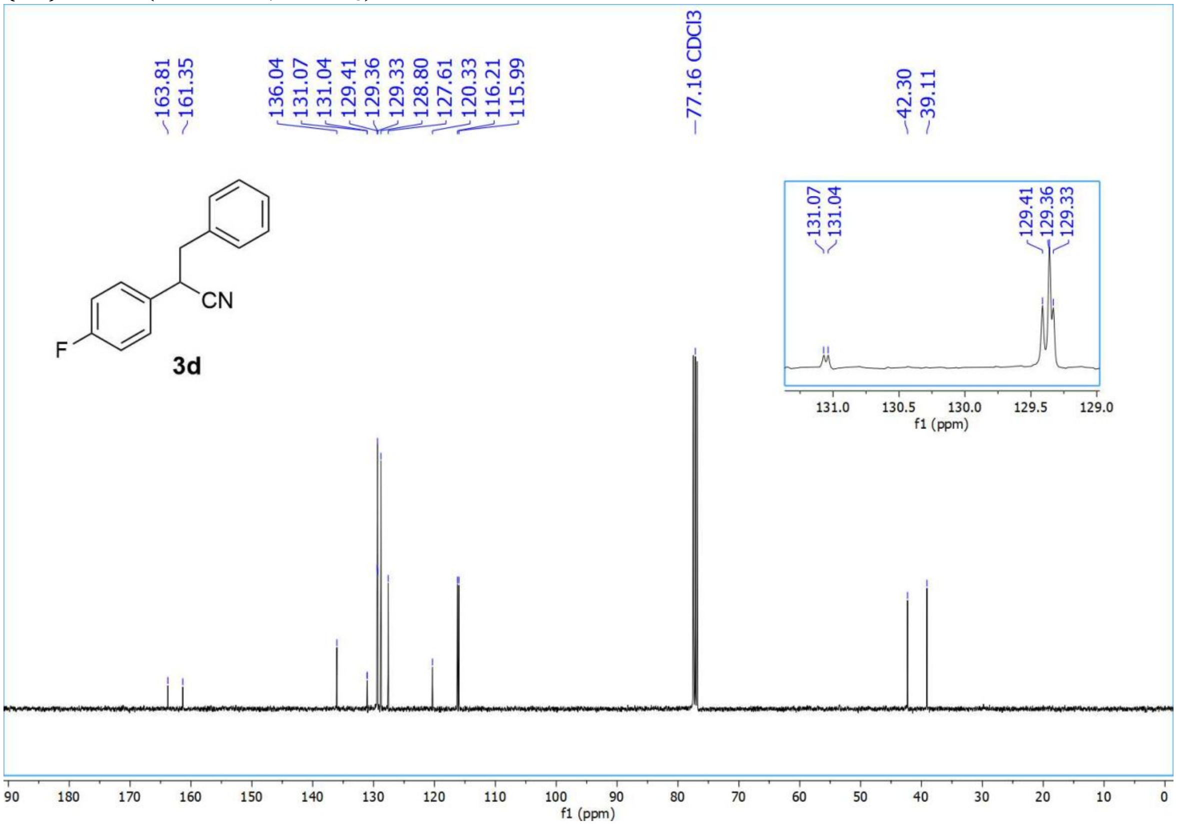

$^{19}F\{^1H\}$ NMR (376 MHz, $CDCl_3$) of 3d:

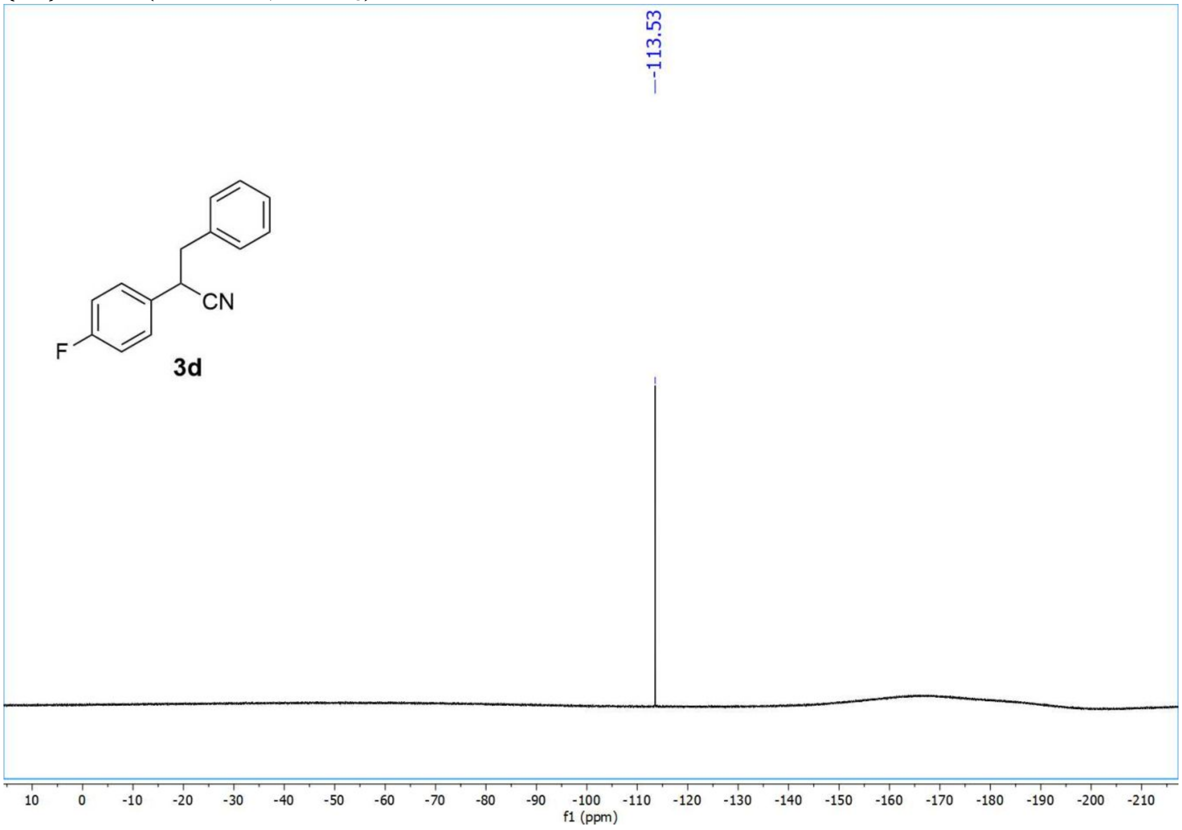

^{1}H NMR (400 MHz, $CDCl_3$) of 3e:

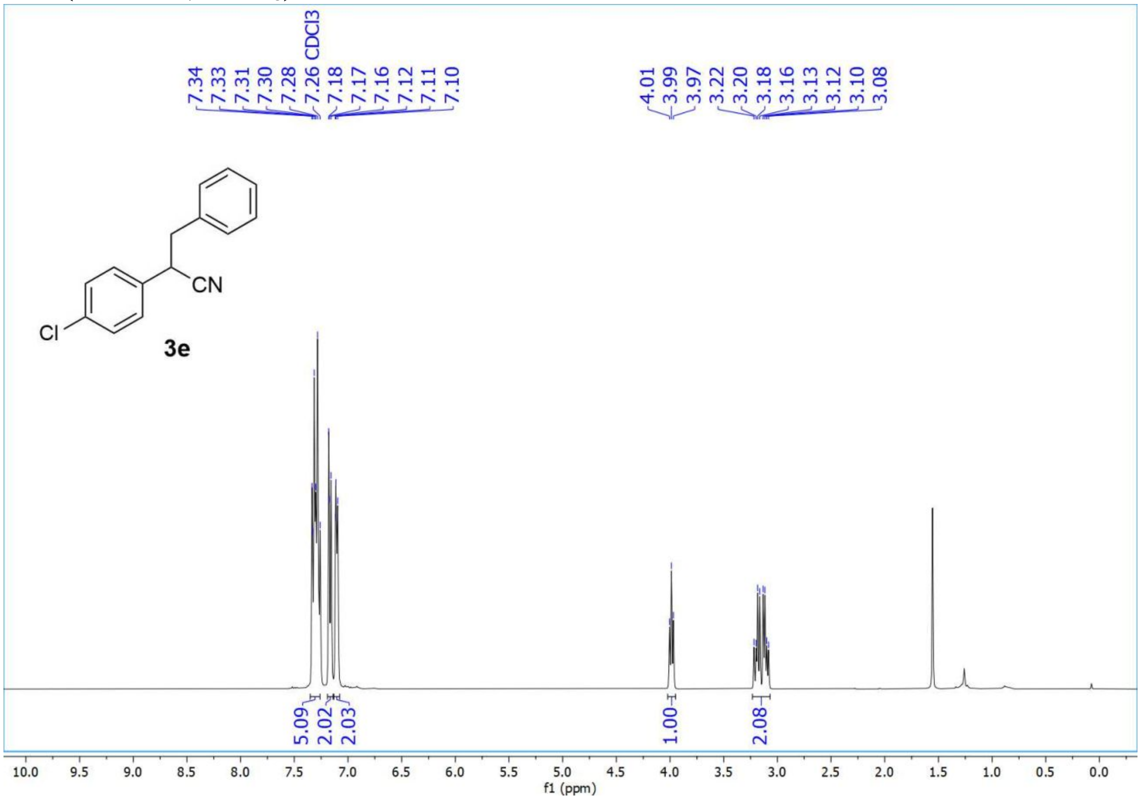

$^{13}C\{^{1}H\}$ NMR (100 MHz, $CDCl_3$) of 3e:

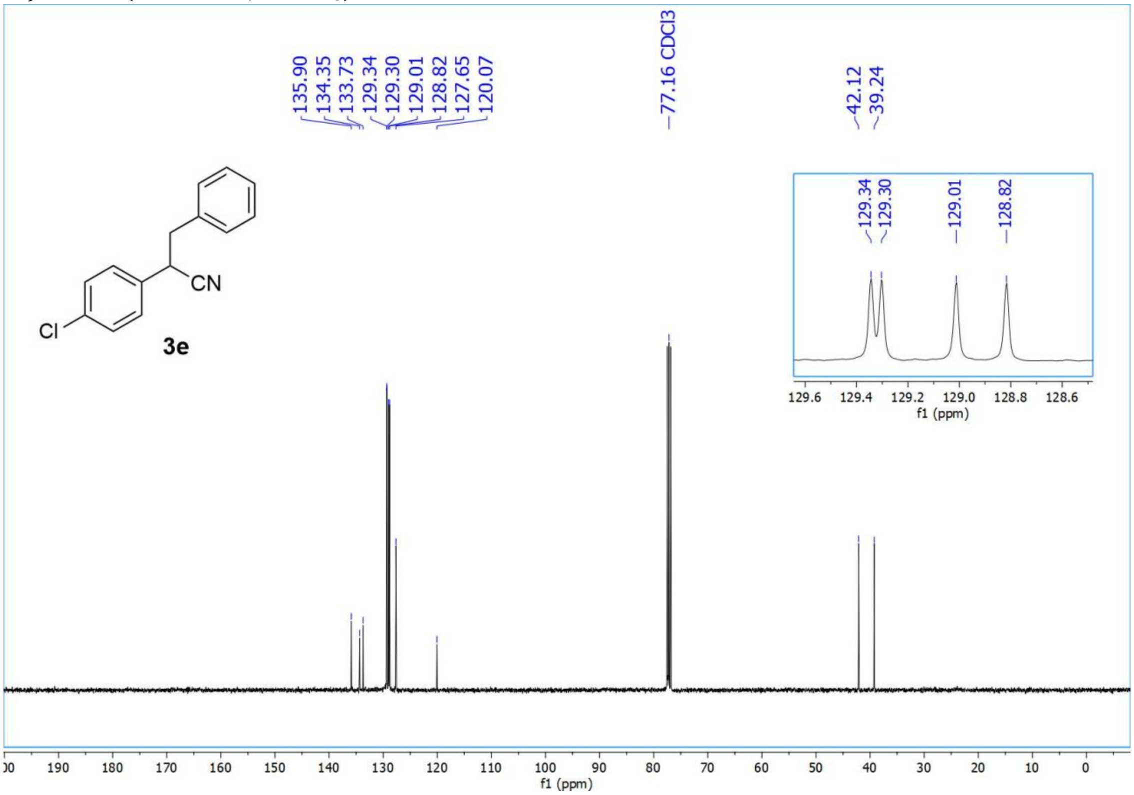

^{1}H NMR (400 MHz, CDCl$_3$) of 3f:

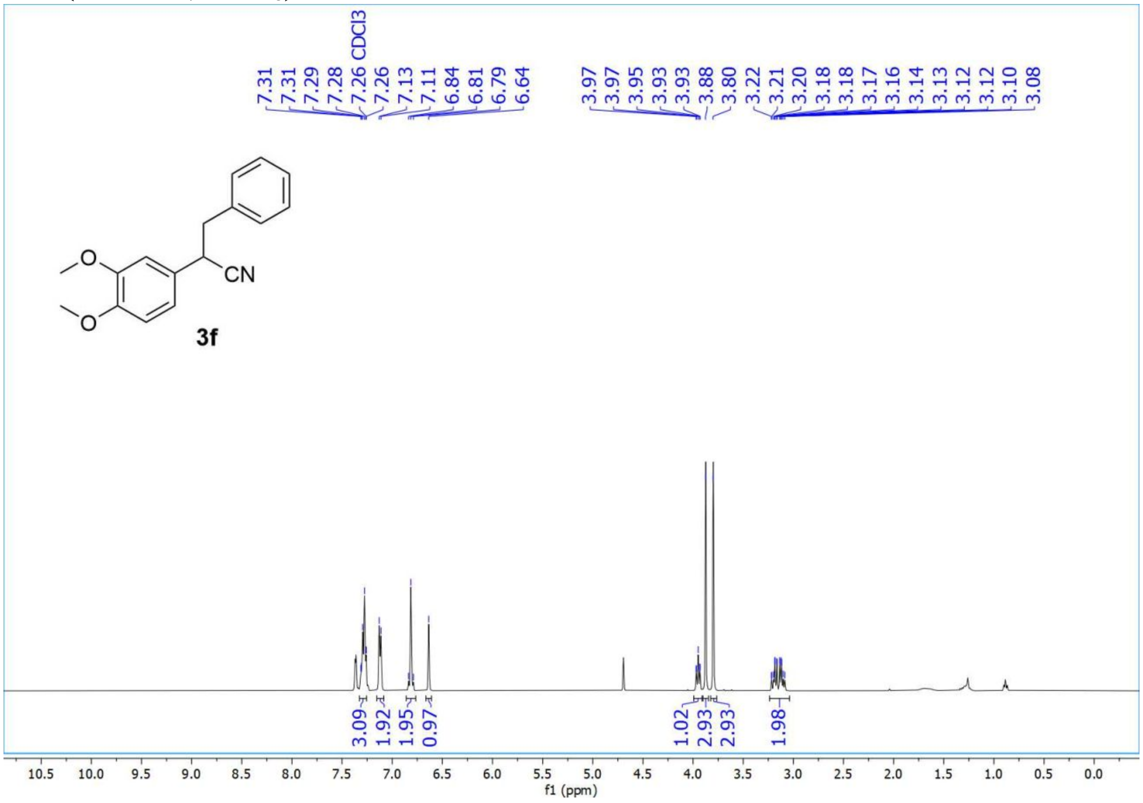

^{13}C{^{1}H} NMR (100 MHz, CDCl$_3$) of 3f:

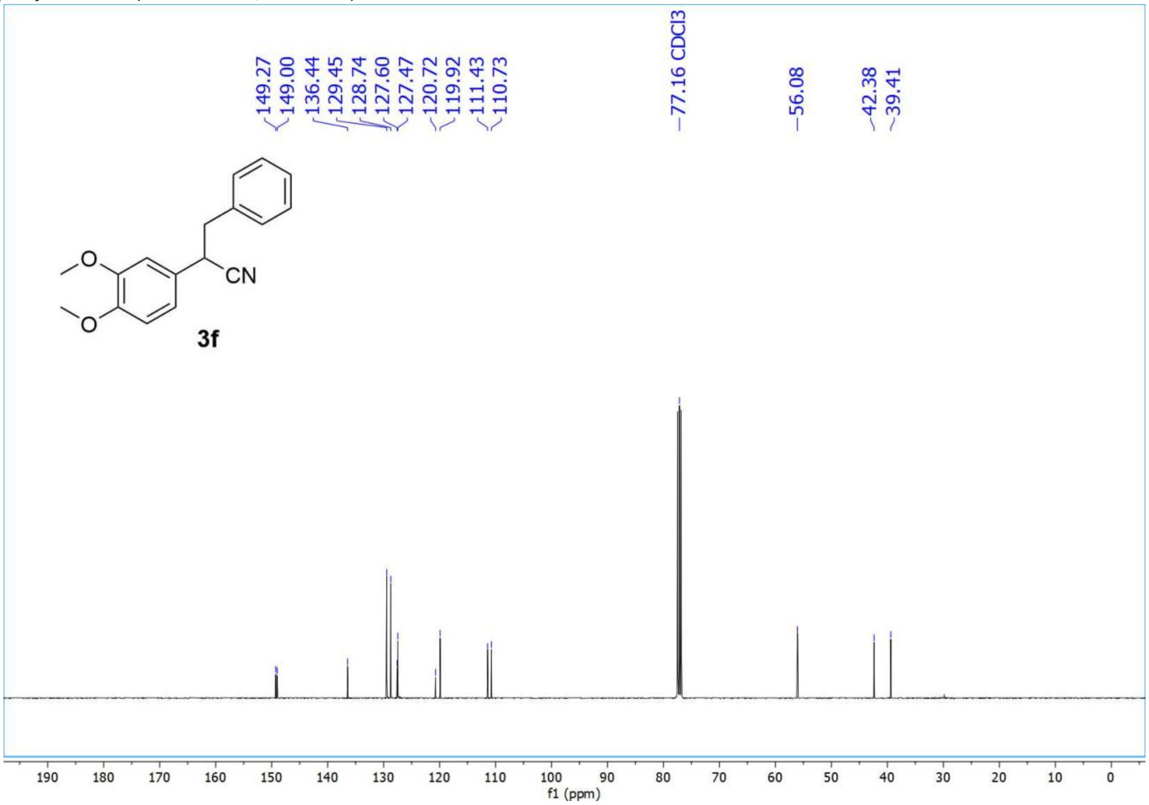

^{1}H NMR (400 MHz, $CDCl_3$) of 3g:

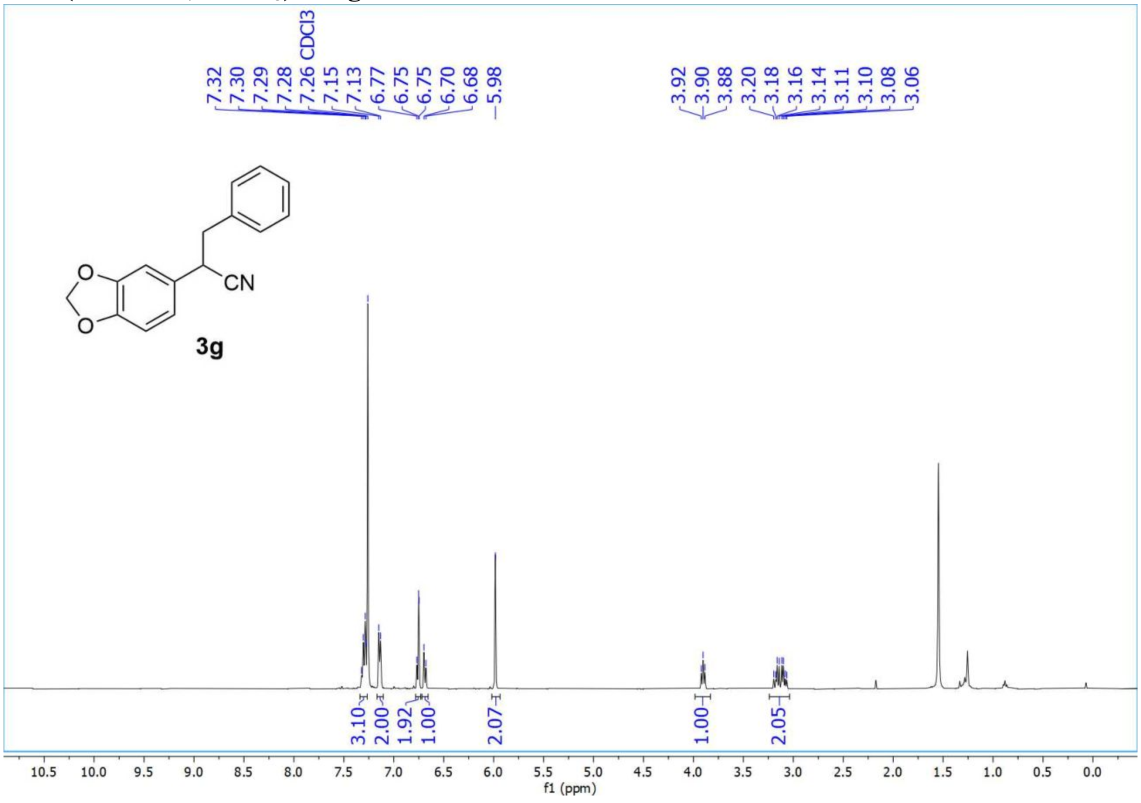

$^{13}C\{^1H\}$ NMR (100 MHz, $CDCl_3$) of 3g:

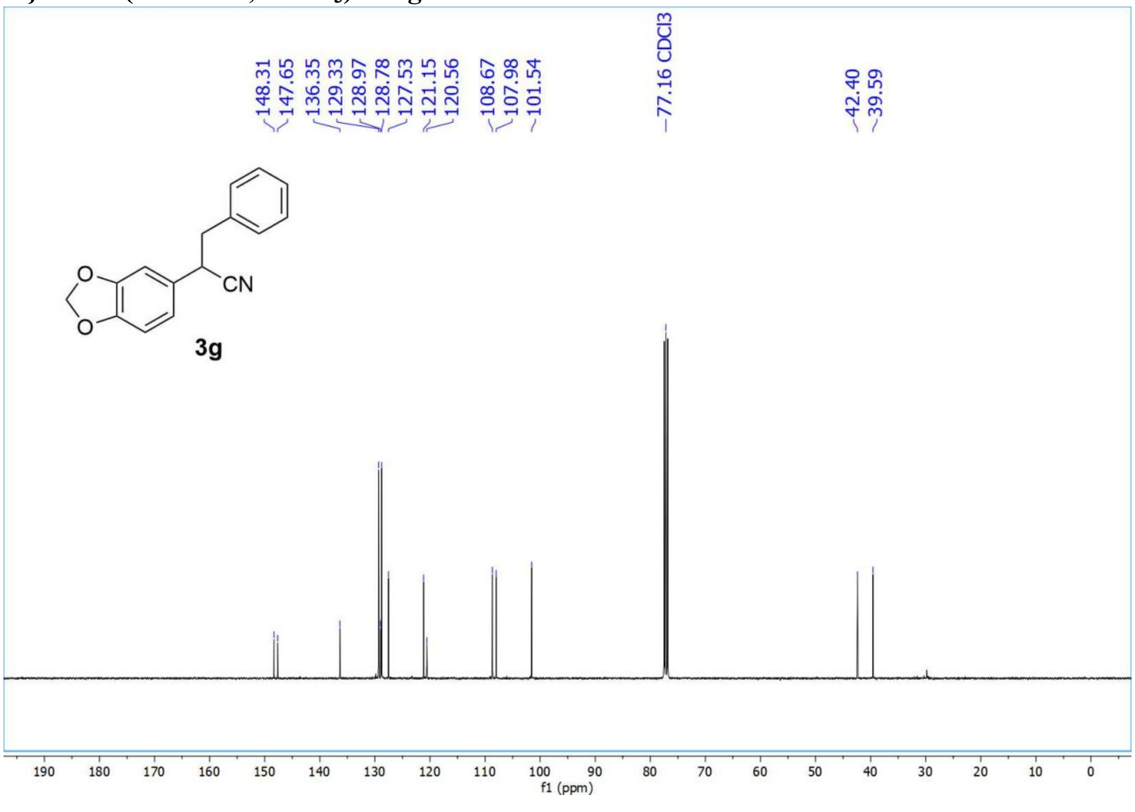

^{1}H NMR (400 MHz, $CDCl_3$) of 3h:

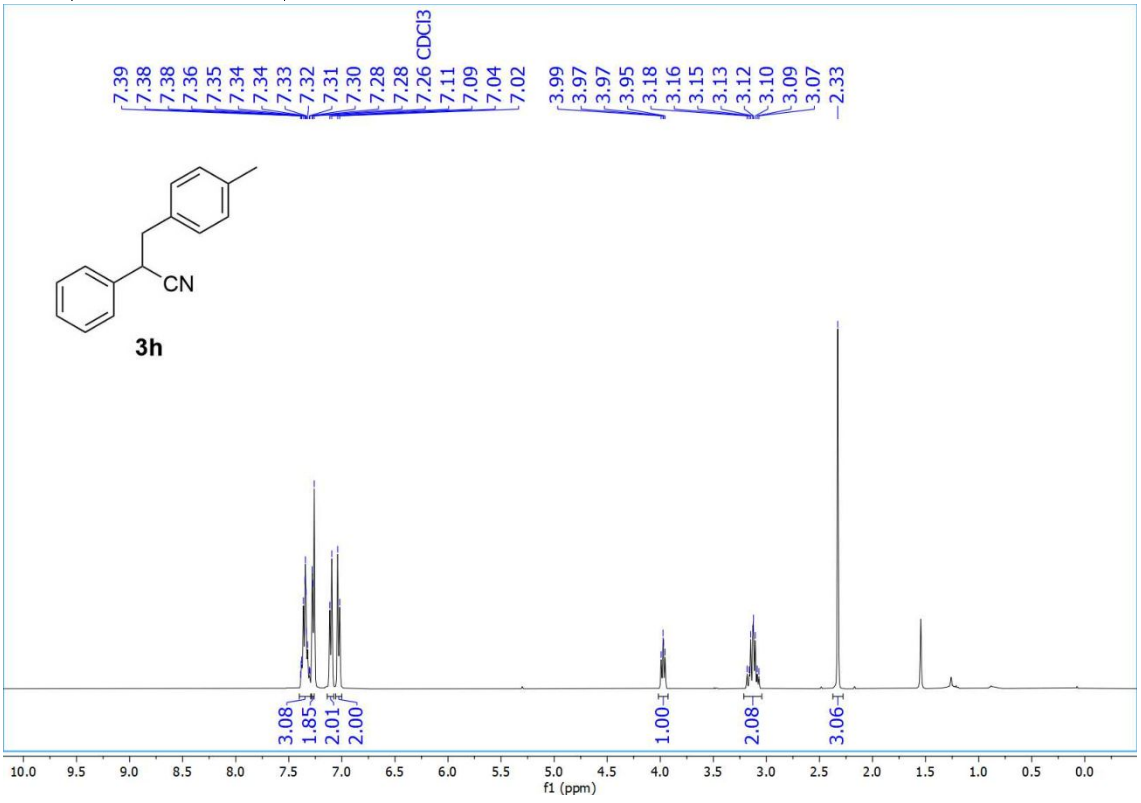

$^{13}C\{^{1}H\}$ NMR (100 MHz, $CDCl_3$) of 3h:

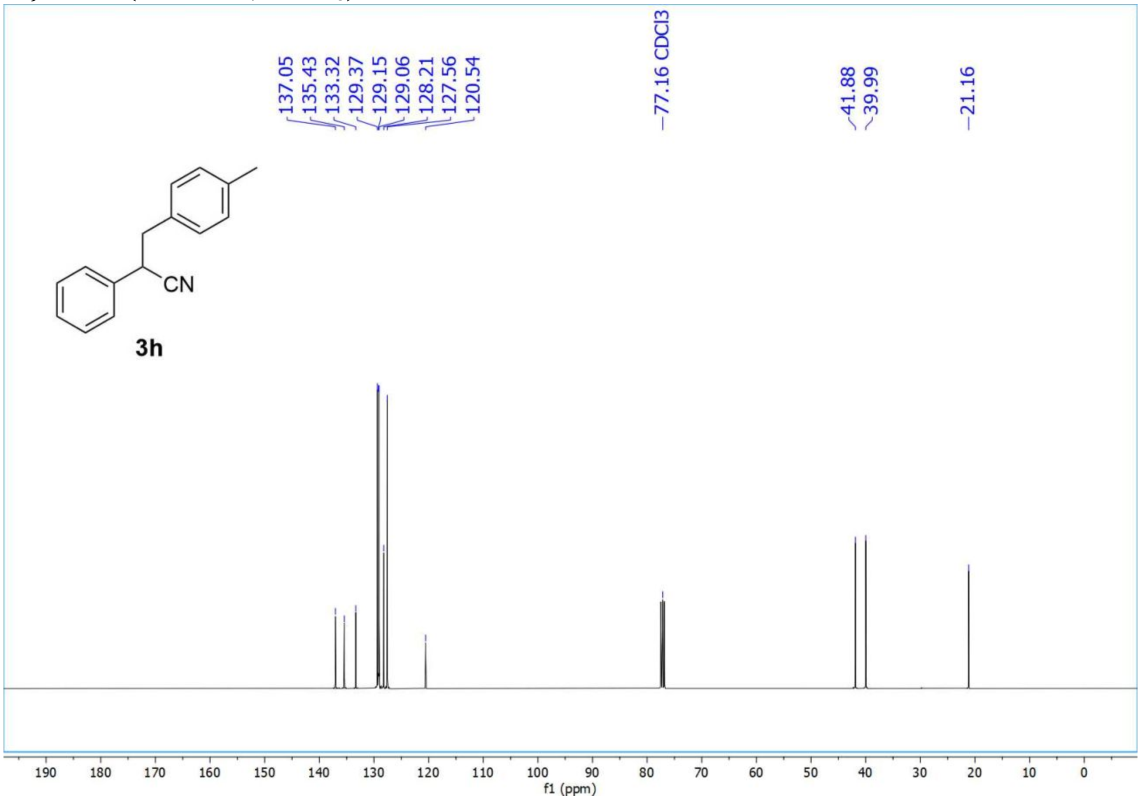

^{1}H NMR (400 MHz, $CDCl_3$) of 3k:

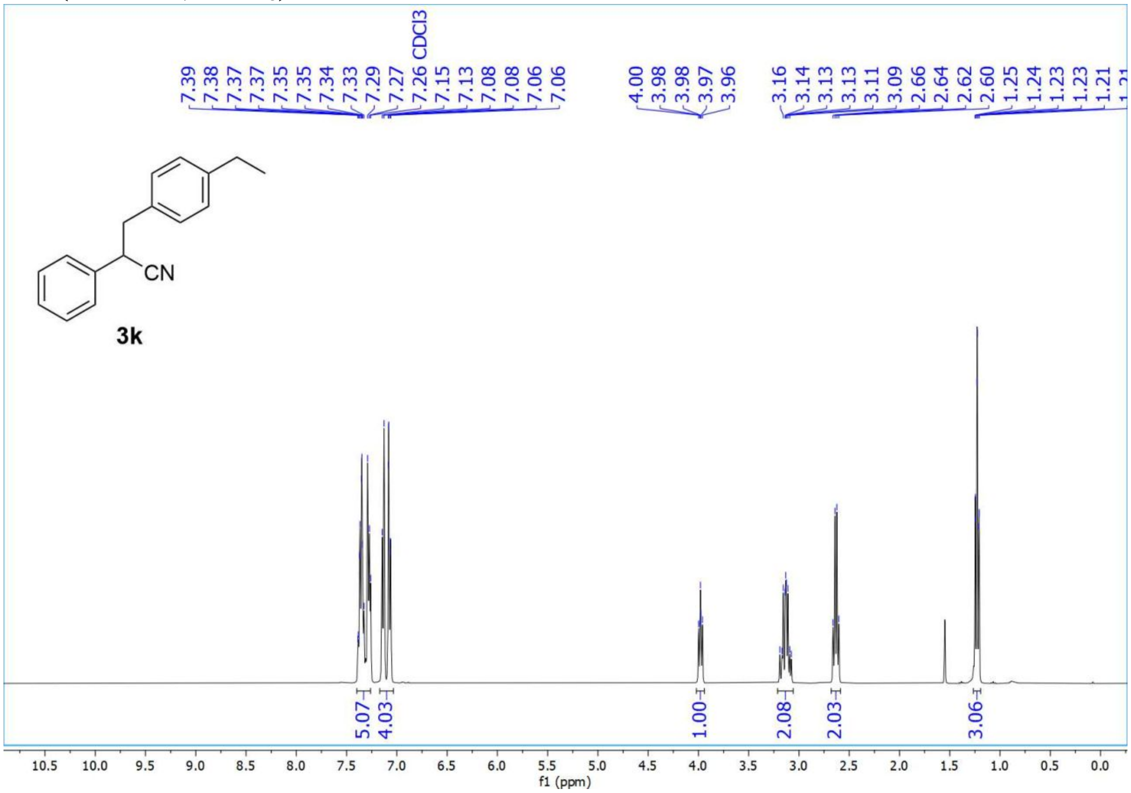

^{13}C{^{1}H} NMR (100 MHz, $CDCl_3$) of 3k:

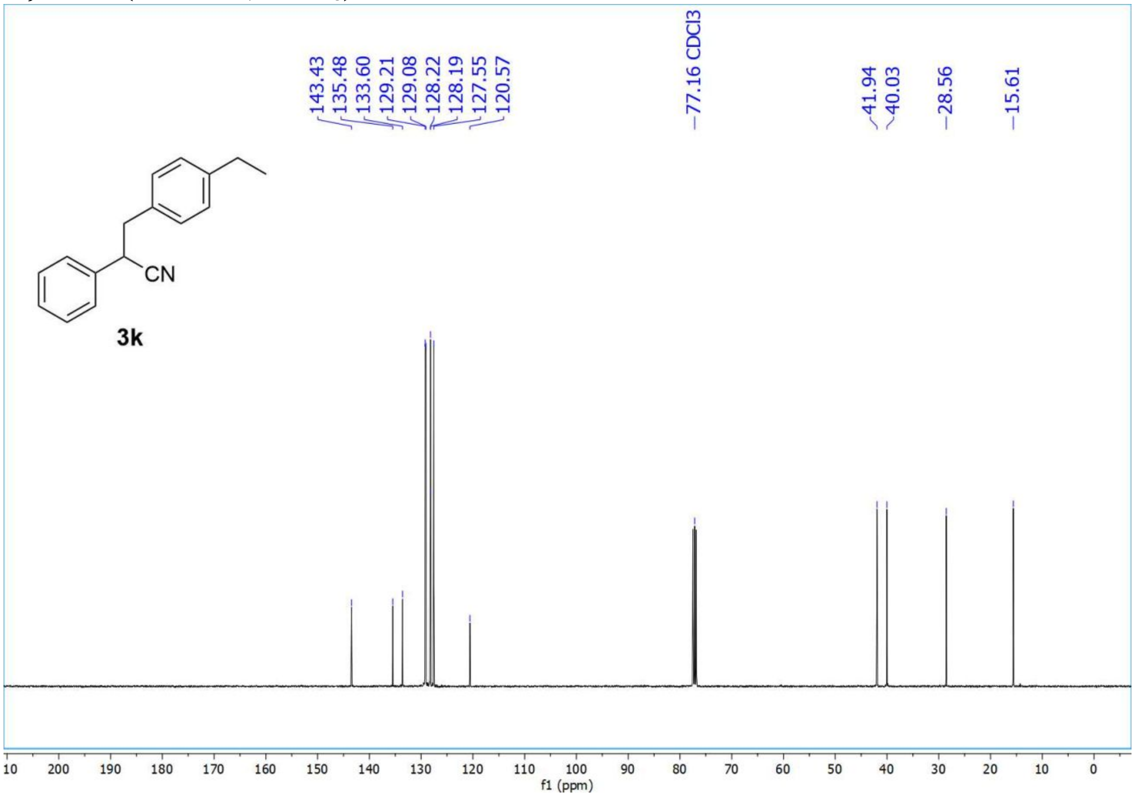

^{1}H NMR (400 MHz, $CDCl_3$) of 3l:

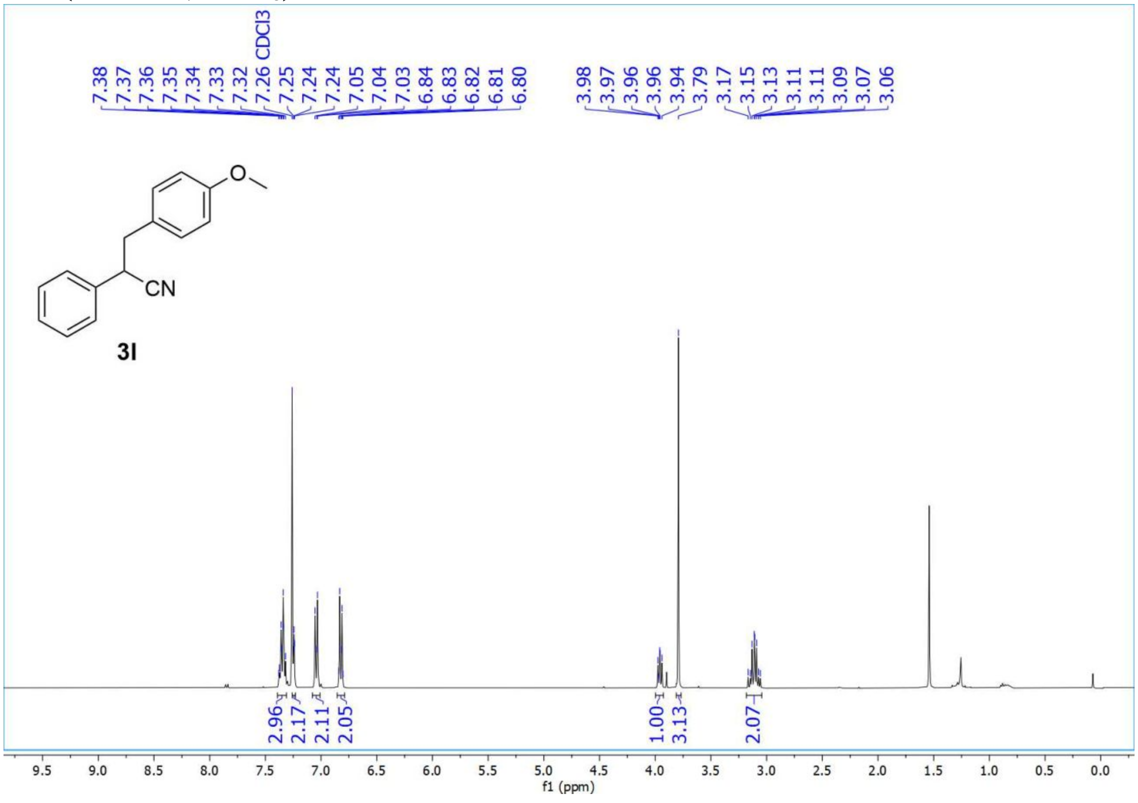

$^{13}C\{^1H\}$ NMR (100 MHz, $CDCl_3$) of 3l:

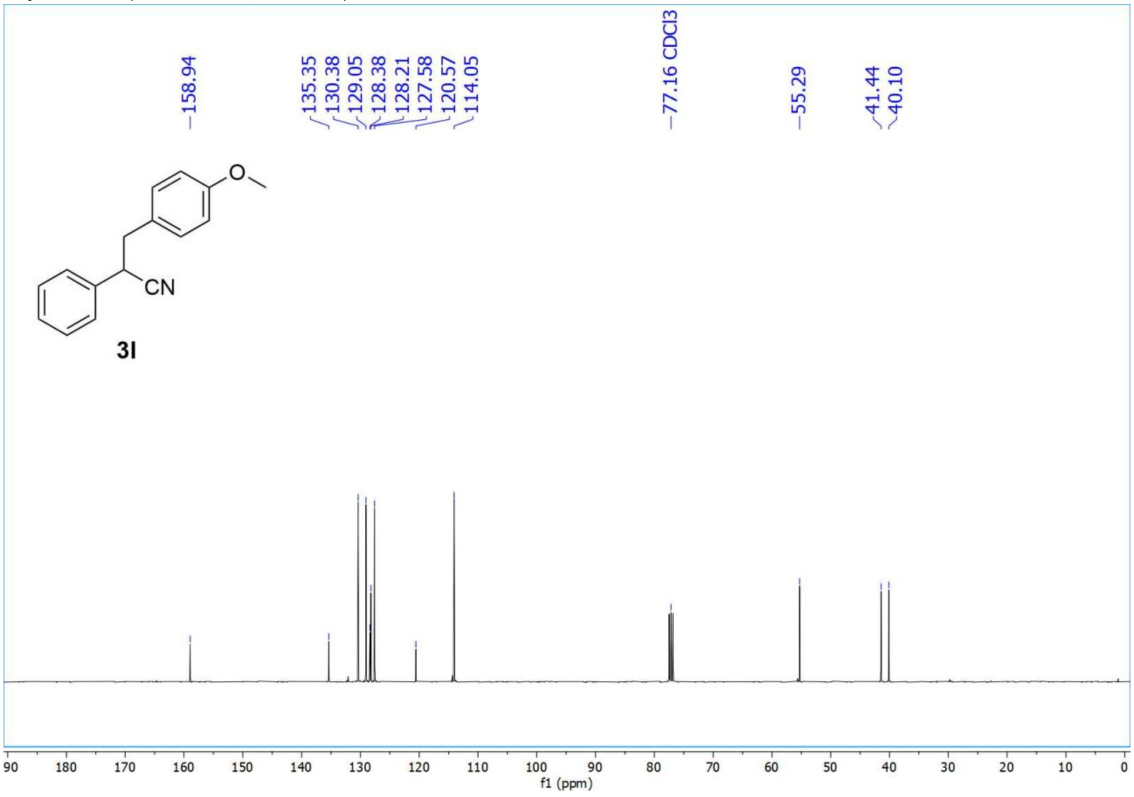

^{1}H NMR (400 MHz, $CDCl_3$) of 3m:

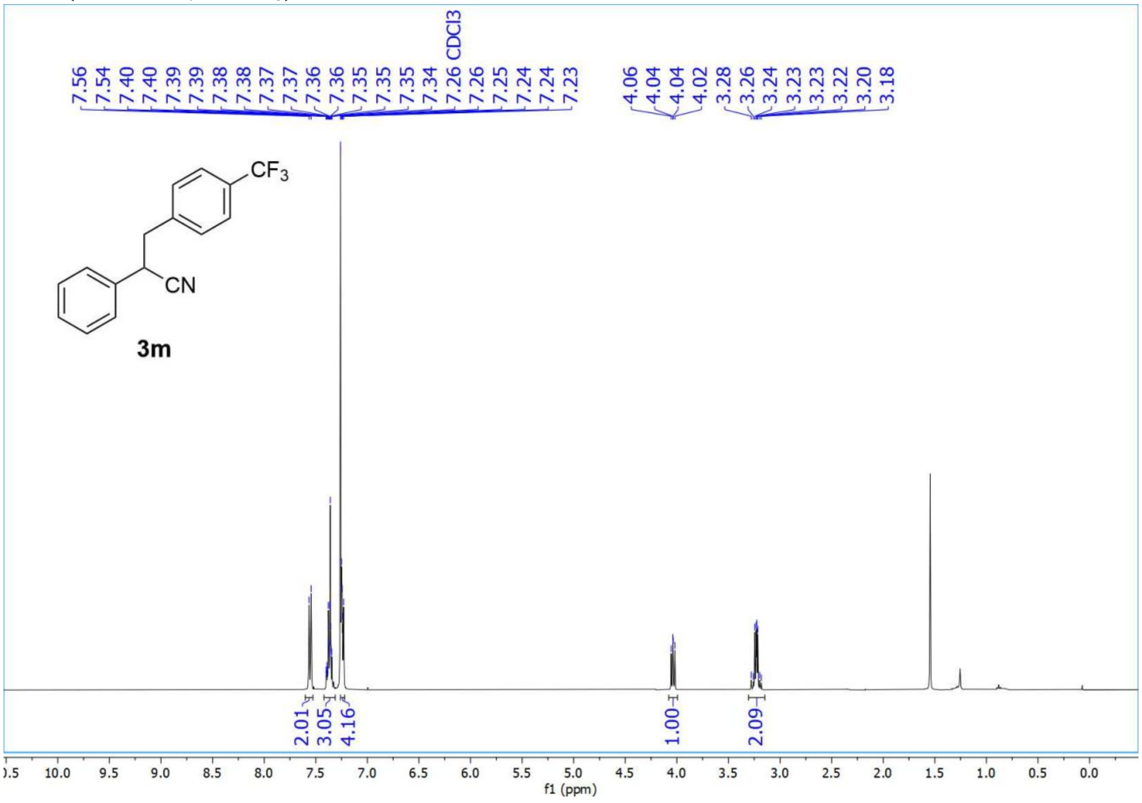

$^{13}C\{^{1}H\}$ NMR (100 MHz, $CDCl_3$) of 3m:

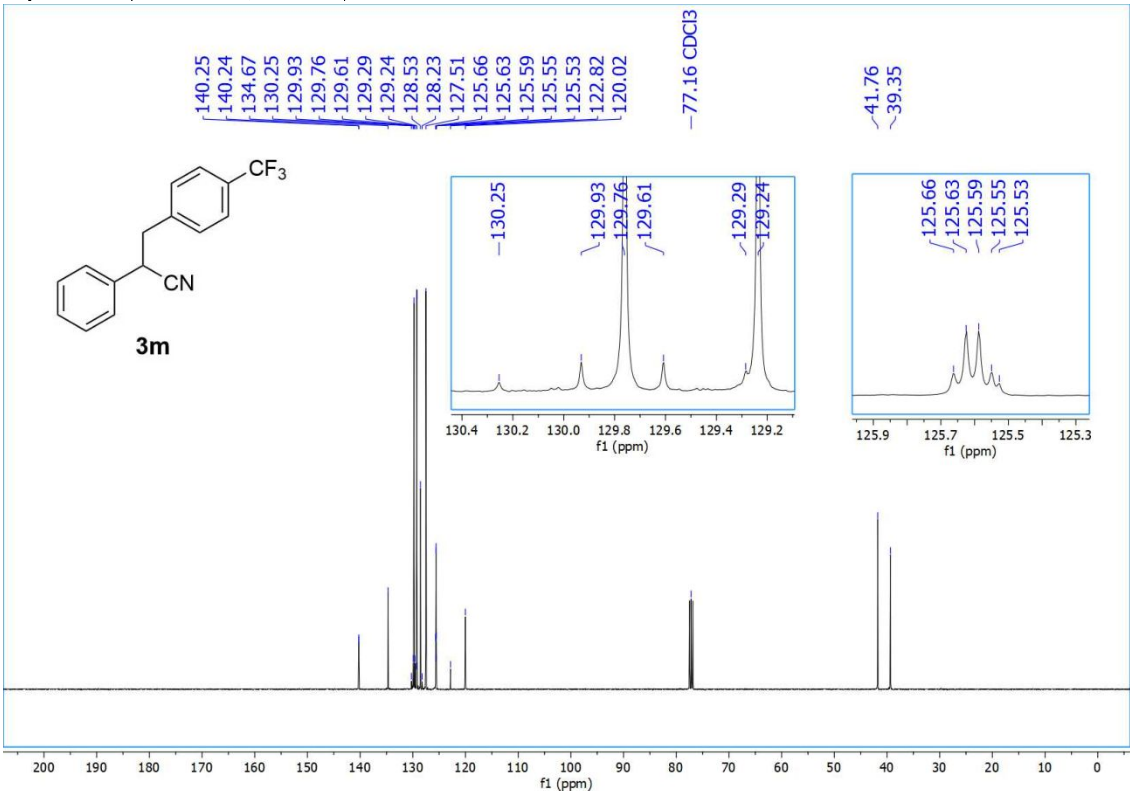

$^{19}F\{^1H\}$ NMR (376 MHz, $CDCl_3$) of 3m:

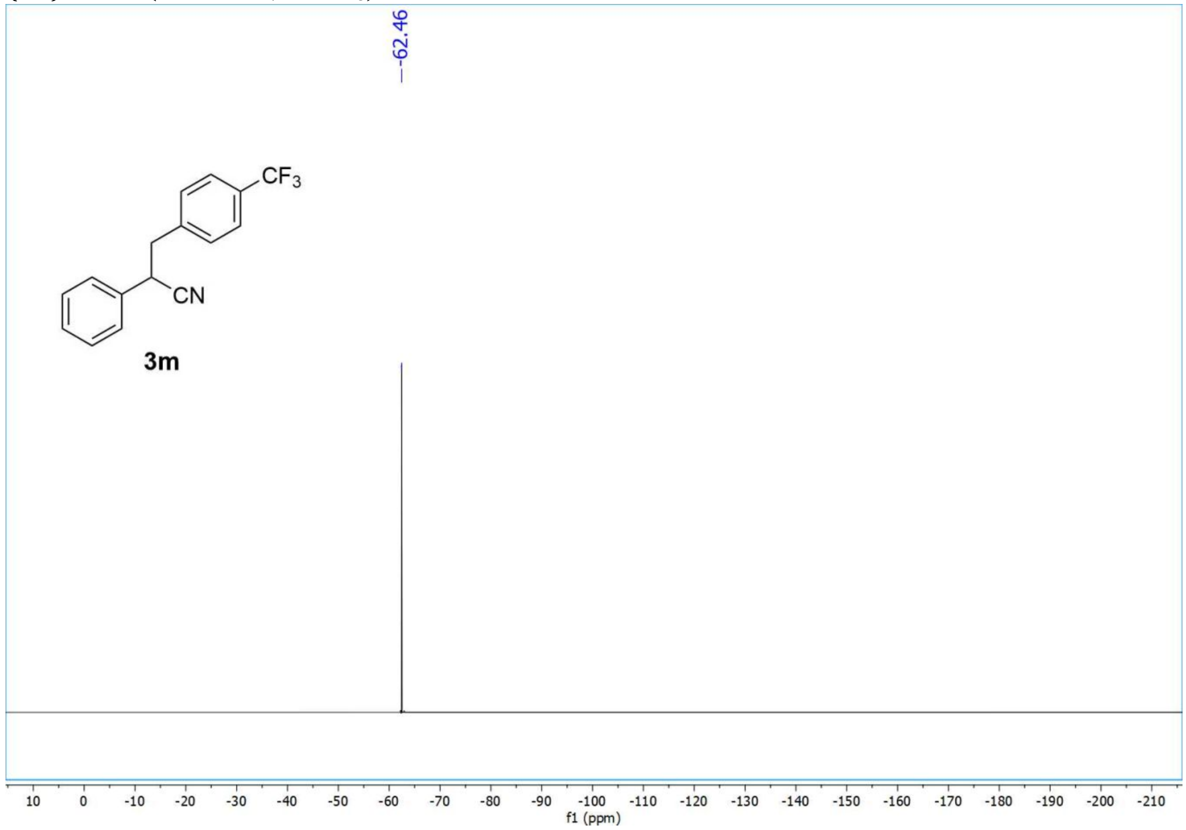

^{1}H NMR (400 MHz, $CDCl_3$) of 3n:

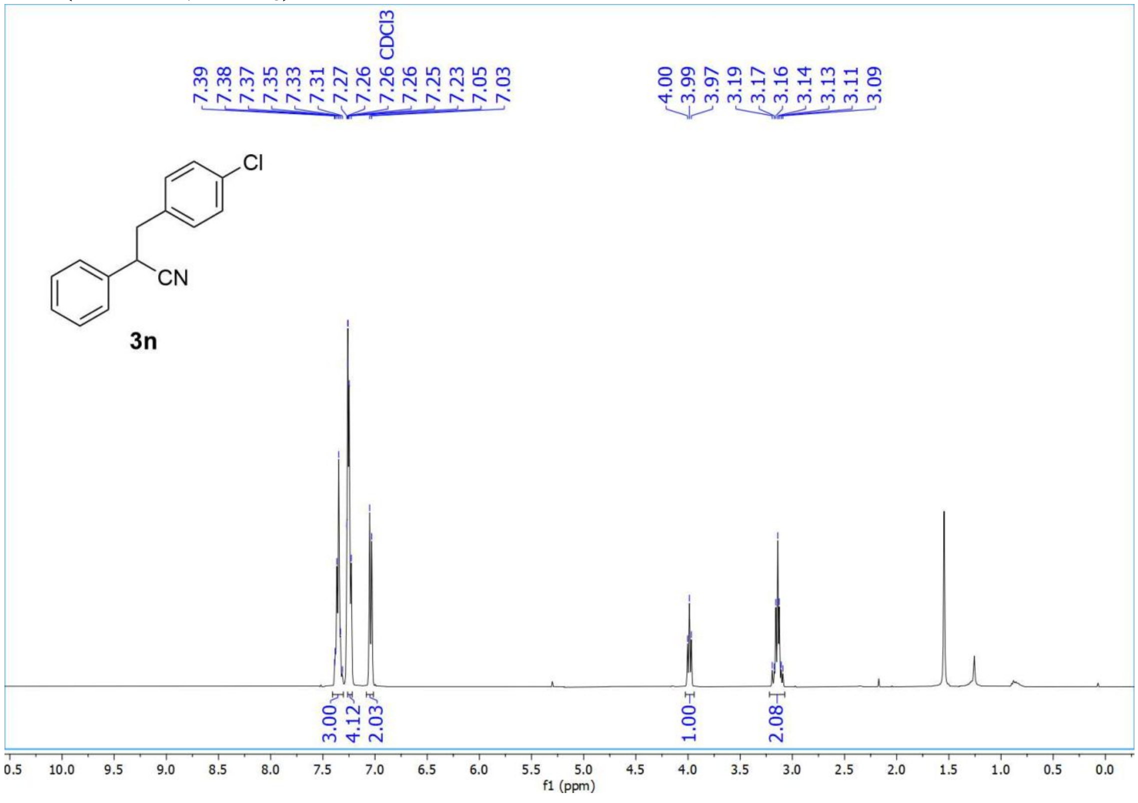

^{13}C{^{1}H} NMR (100 MHz, $CDCl_3$) of 3n:

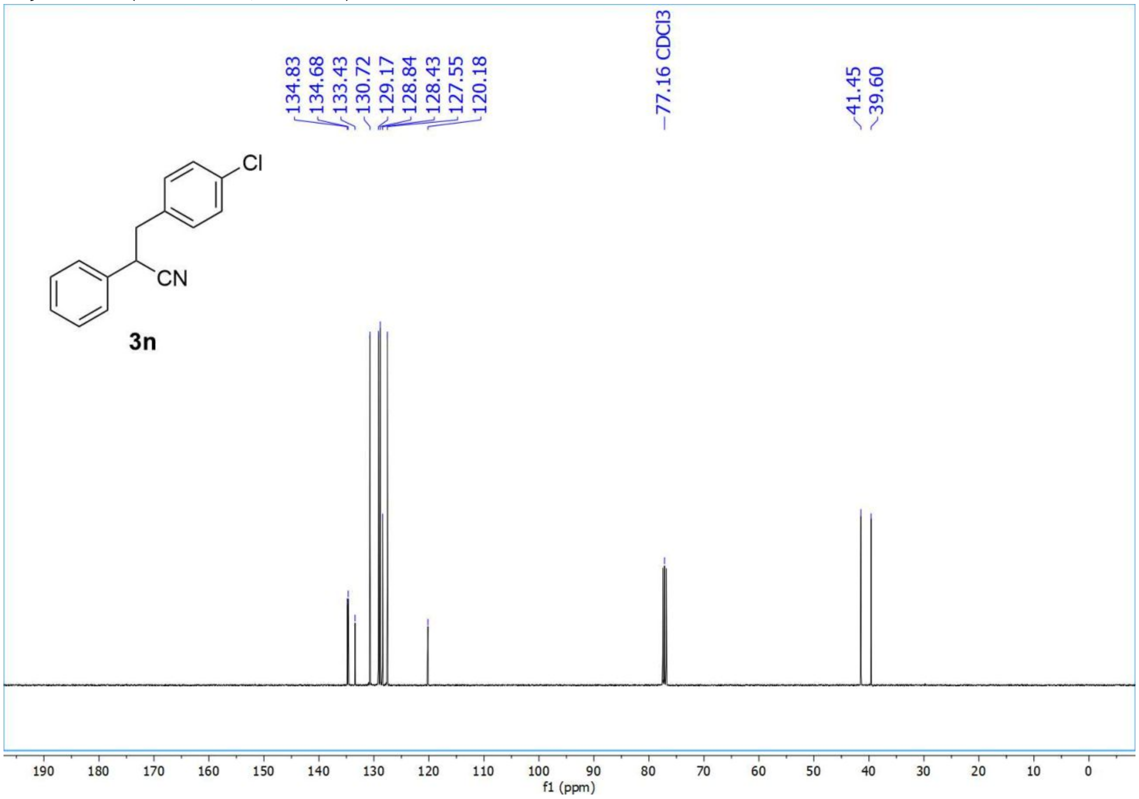

^{1}H NMR (400 MHz, $CDCl_3$) of 3o:

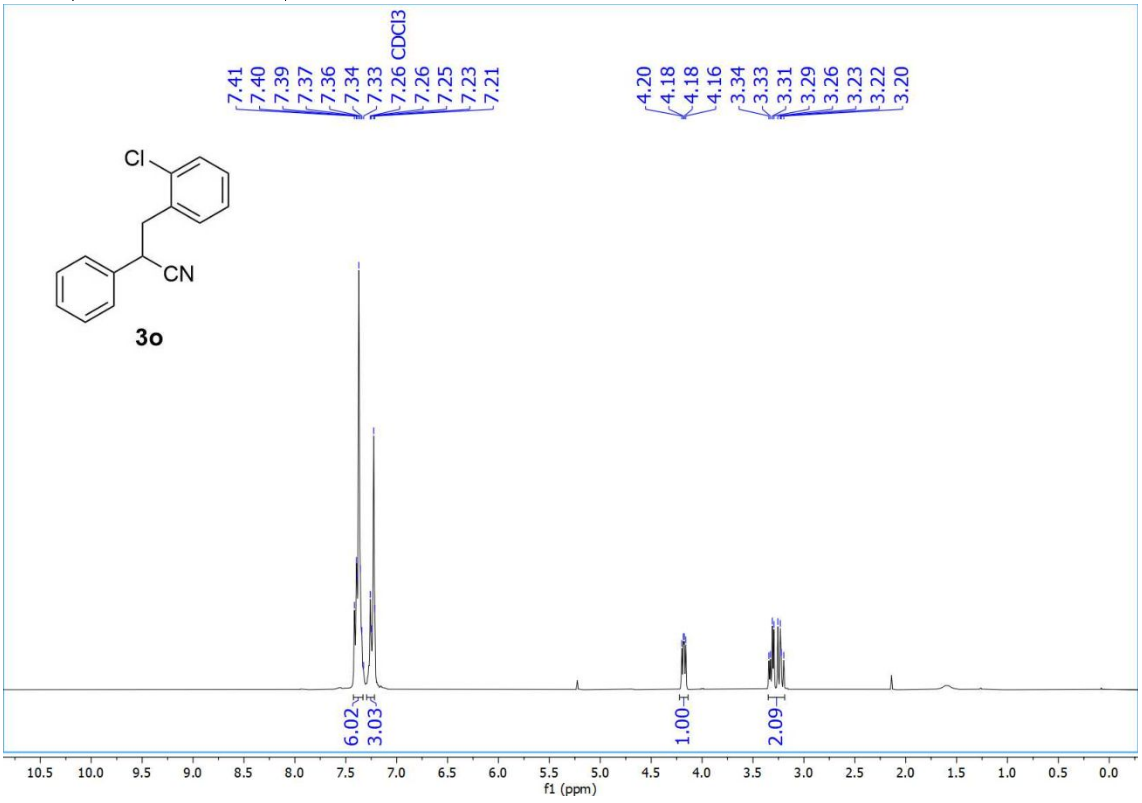

$^{13}C\{^{1}H\}$ NMR (100 MHz, $CDCl_3$) of 3o:

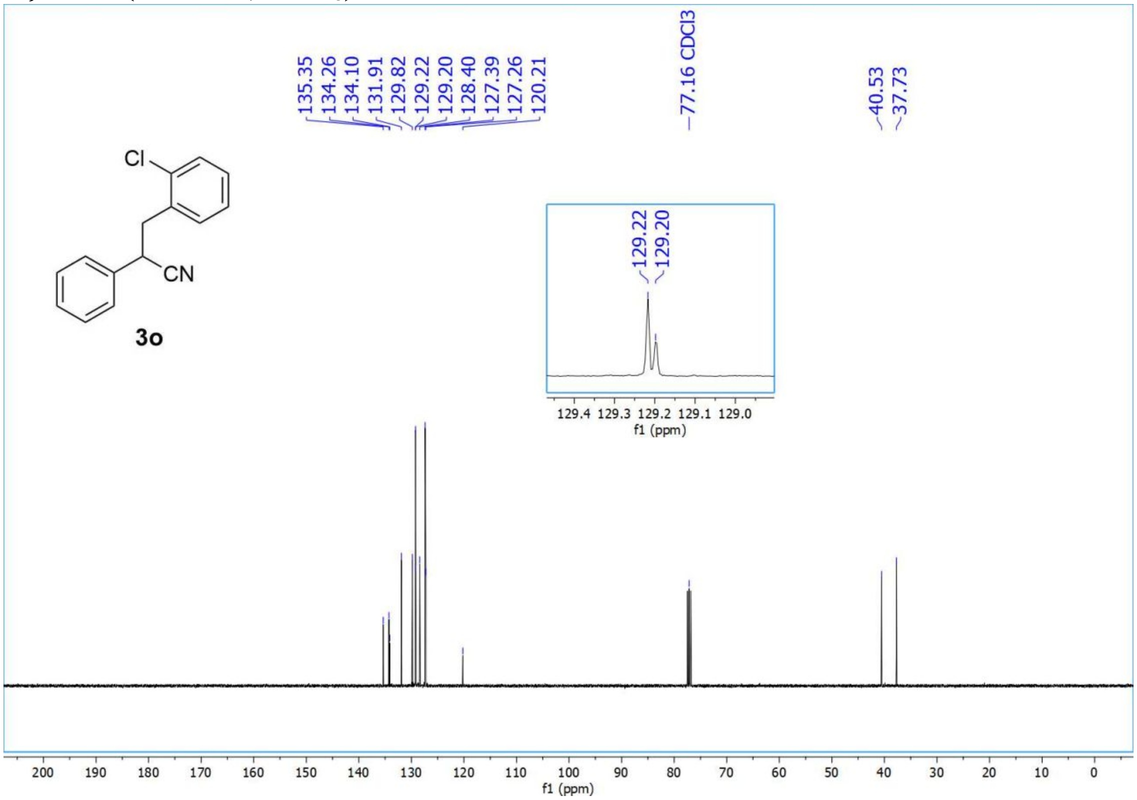

^{1}H NMR (400 MHz, $CDCl_3$) of 3p:

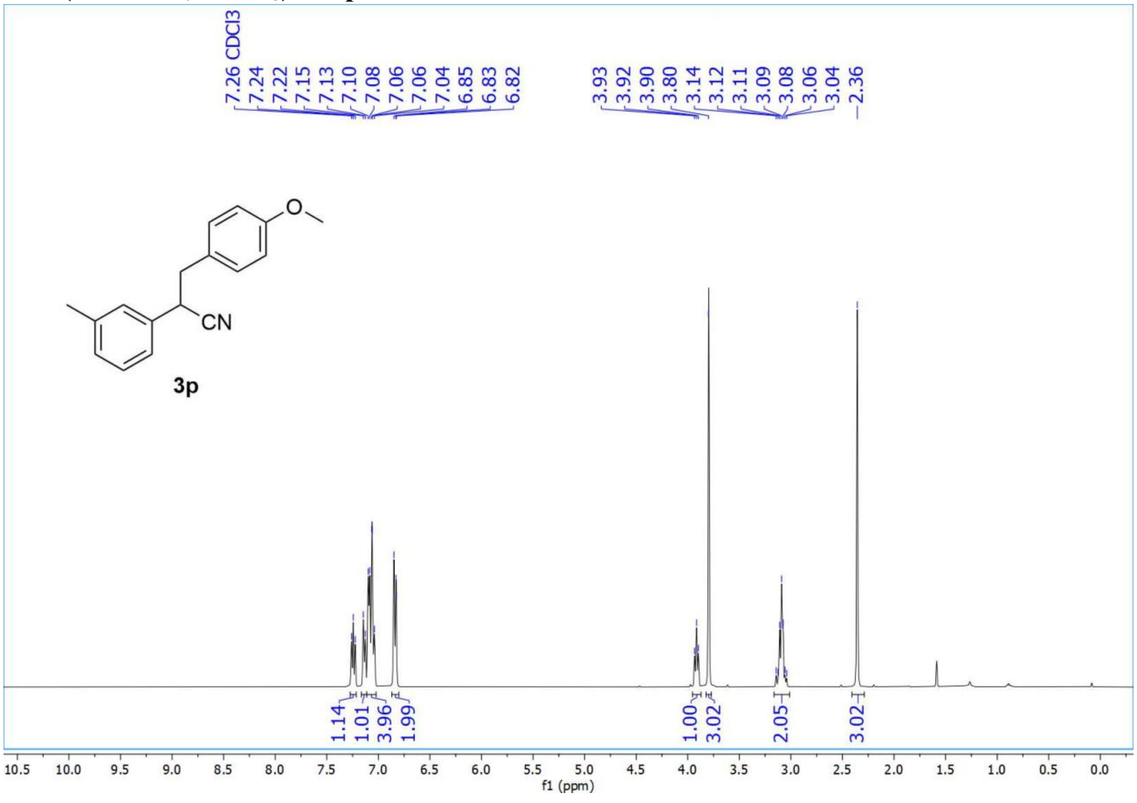

^{13}C{^{1}H} NMR (100 MHz, $CDCl_3$) of 3p:

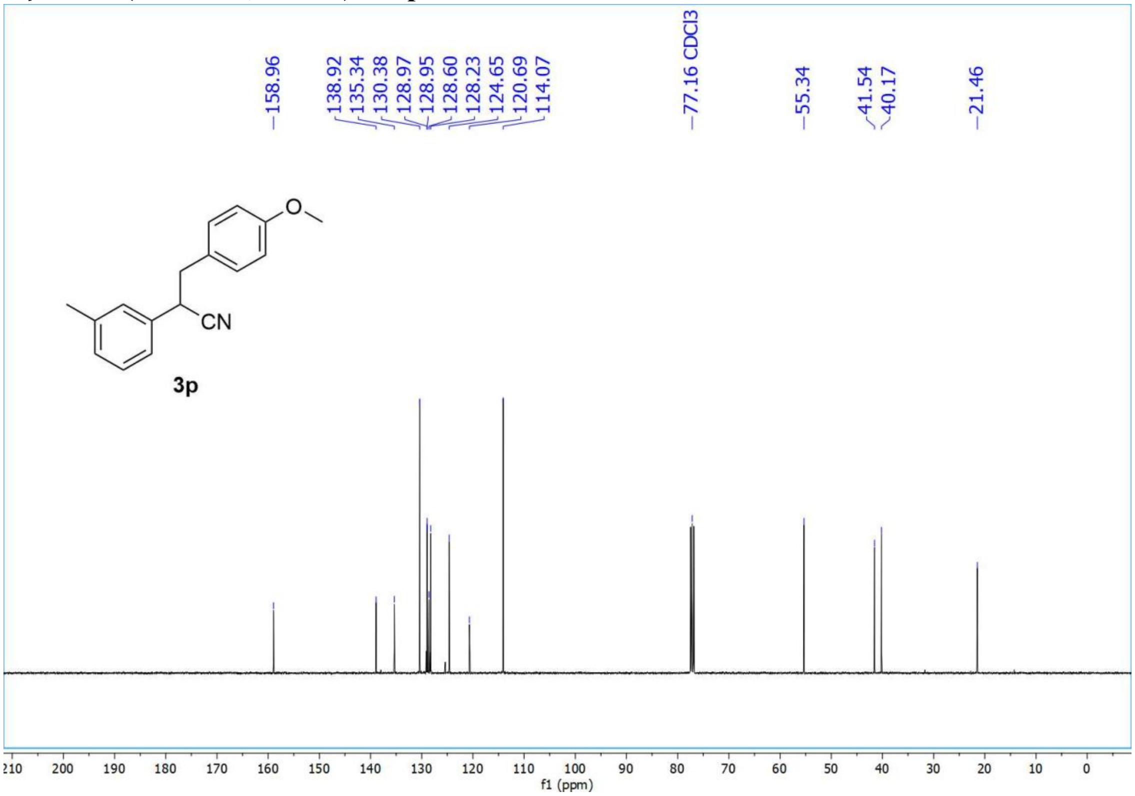

^{1}H NMR (400 MHz, $CDCl_3$) of 3q:

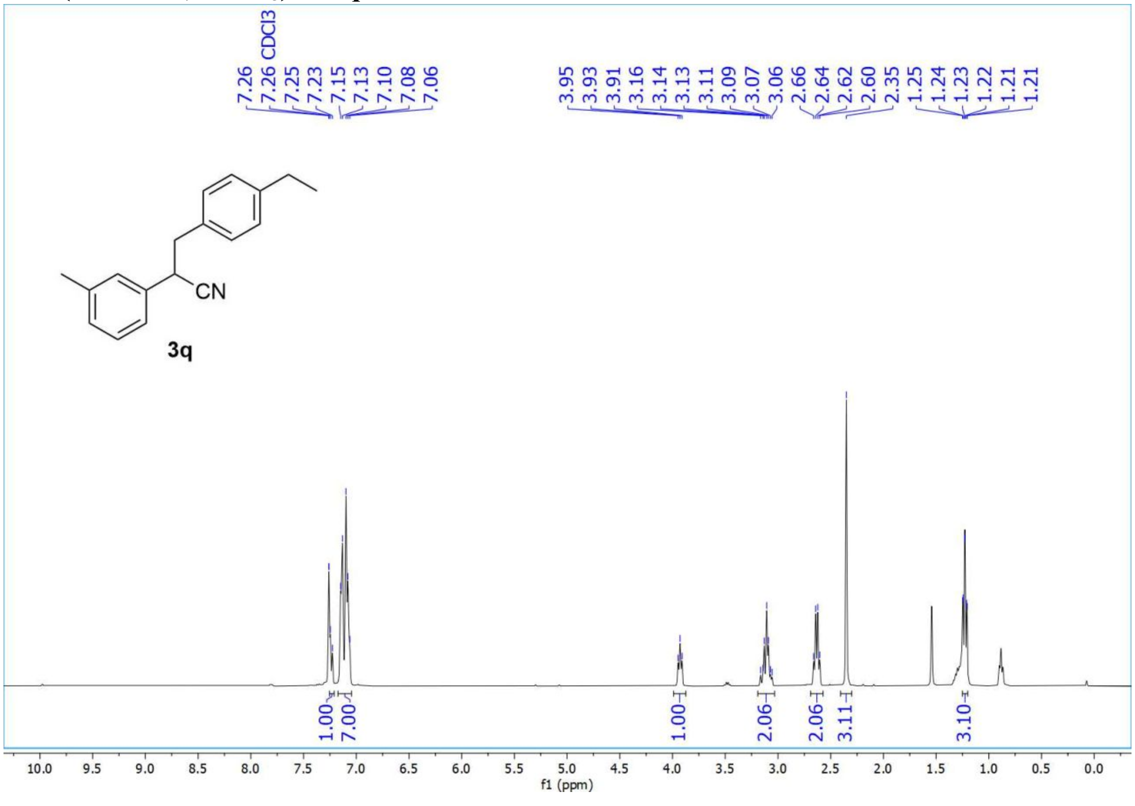

$^{13}C\{^{1}H\}$ NMR (100 MHz, $CDCl_3$) of 3q:

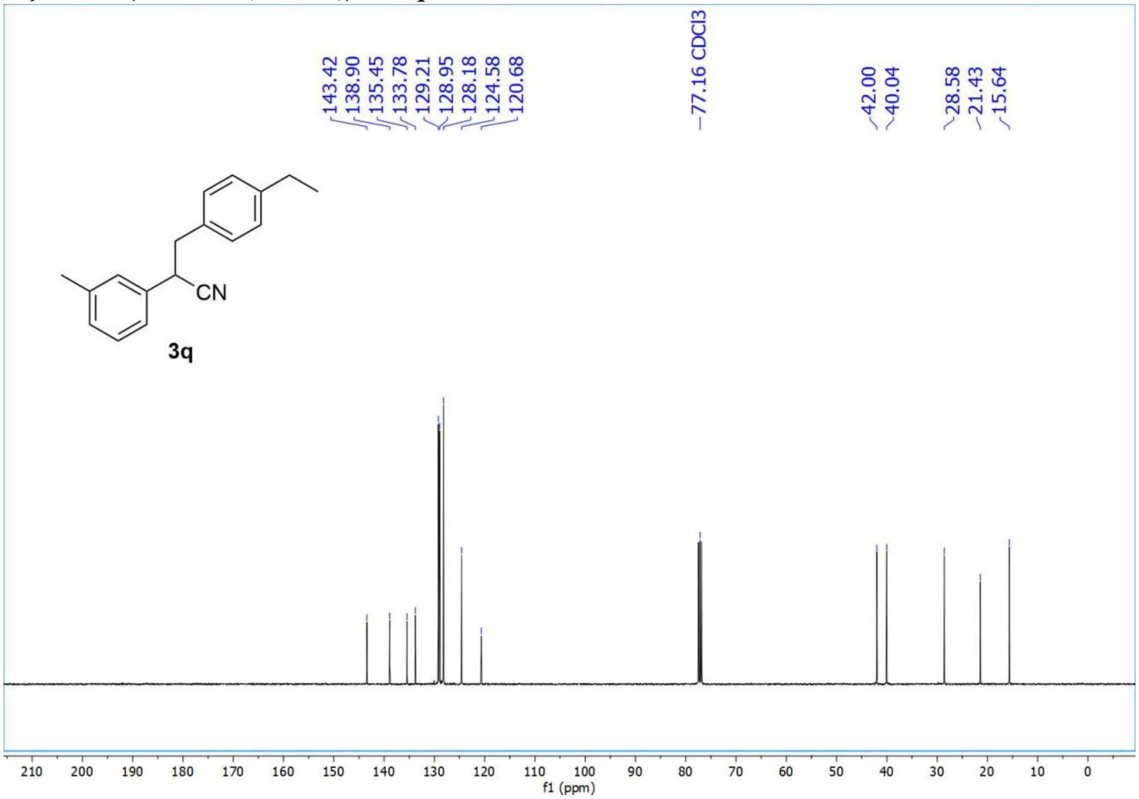

^{1}H NMR (500 MHz, $CDCl_3$) of 3r:

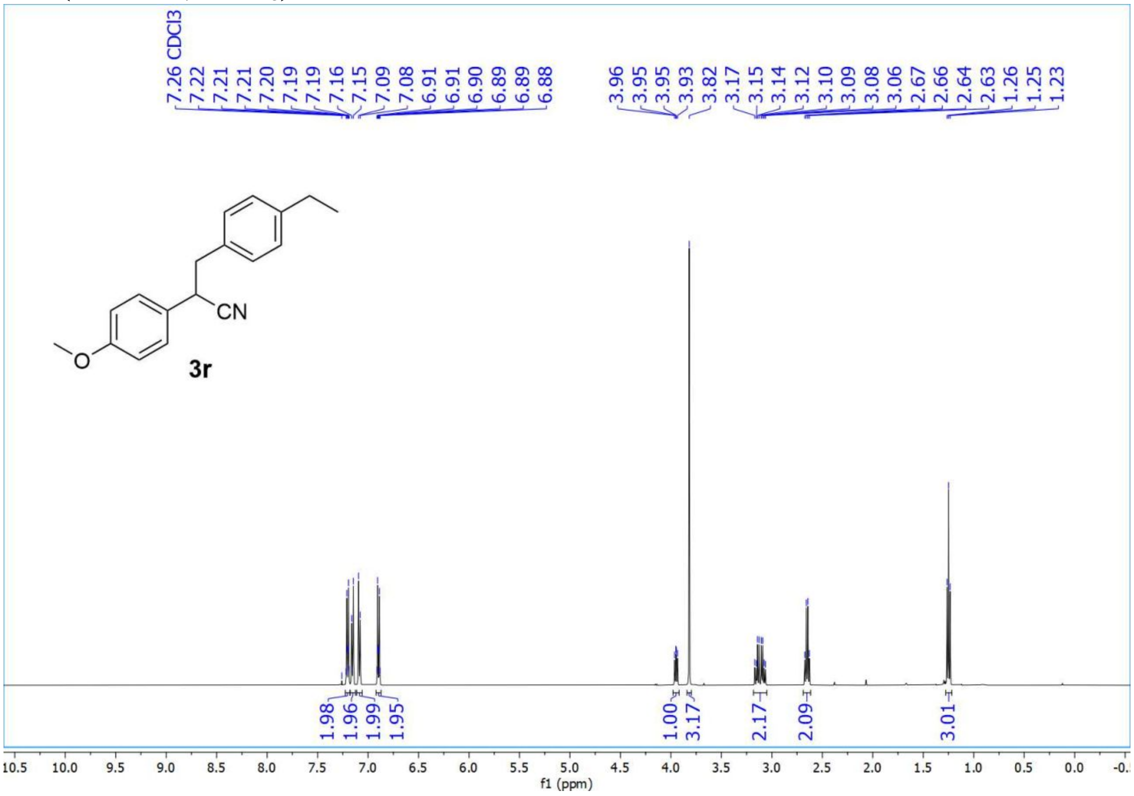

^{13}C{^{1}H} NMR (125 MHz, $CDCl_3$) of 3r:

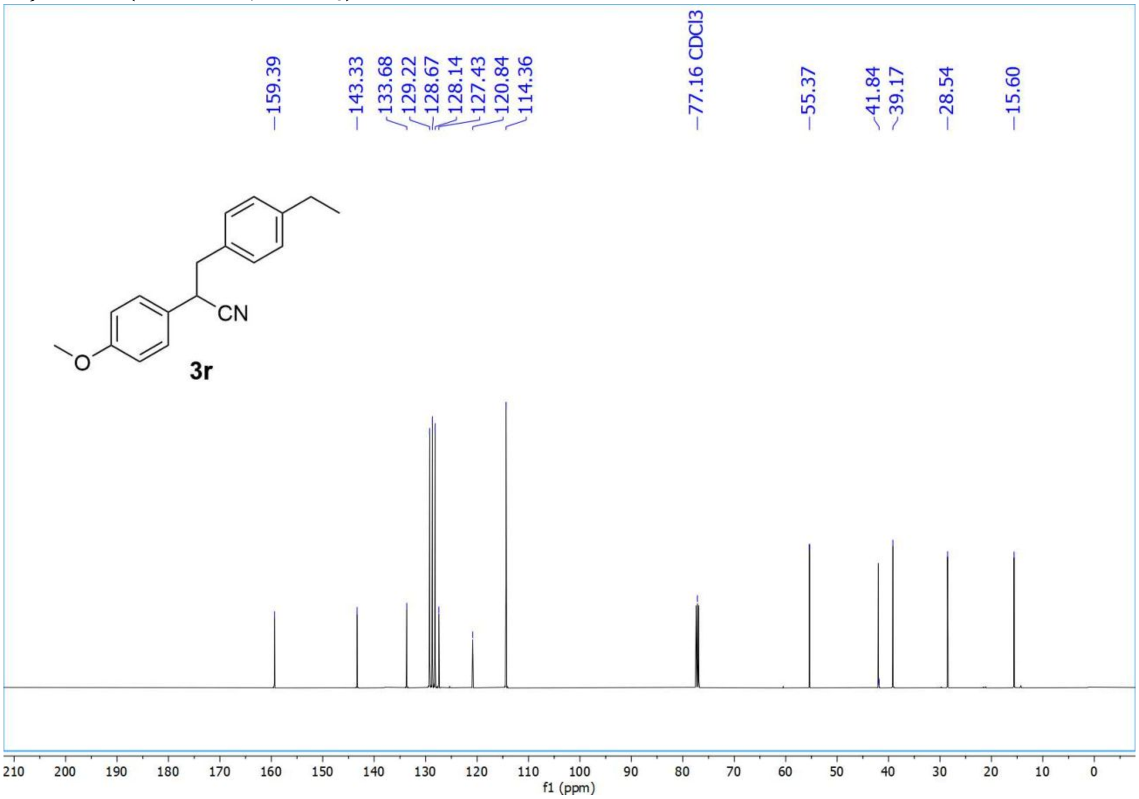

^{1}H NMR (400 MHz, $CDCl_3$) of 3s:

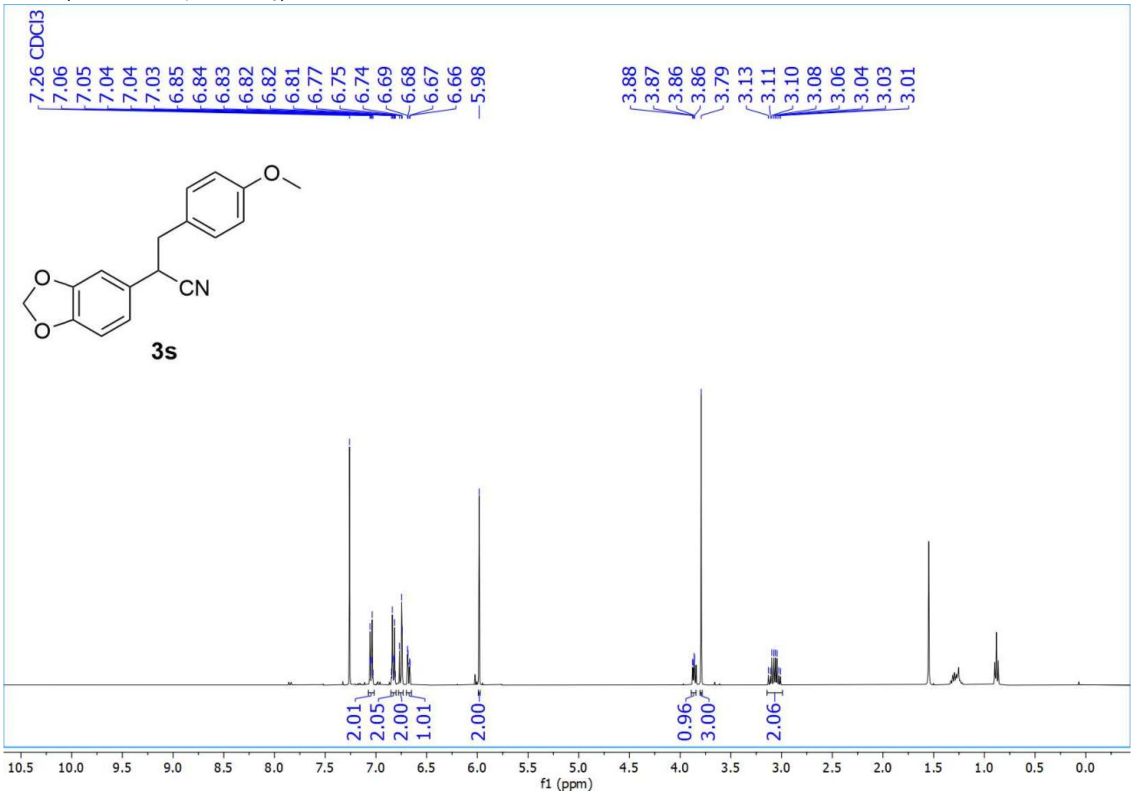

^{13}C{^{1}H} NMR (100 MHz, $CDCl_3$) of 3s:

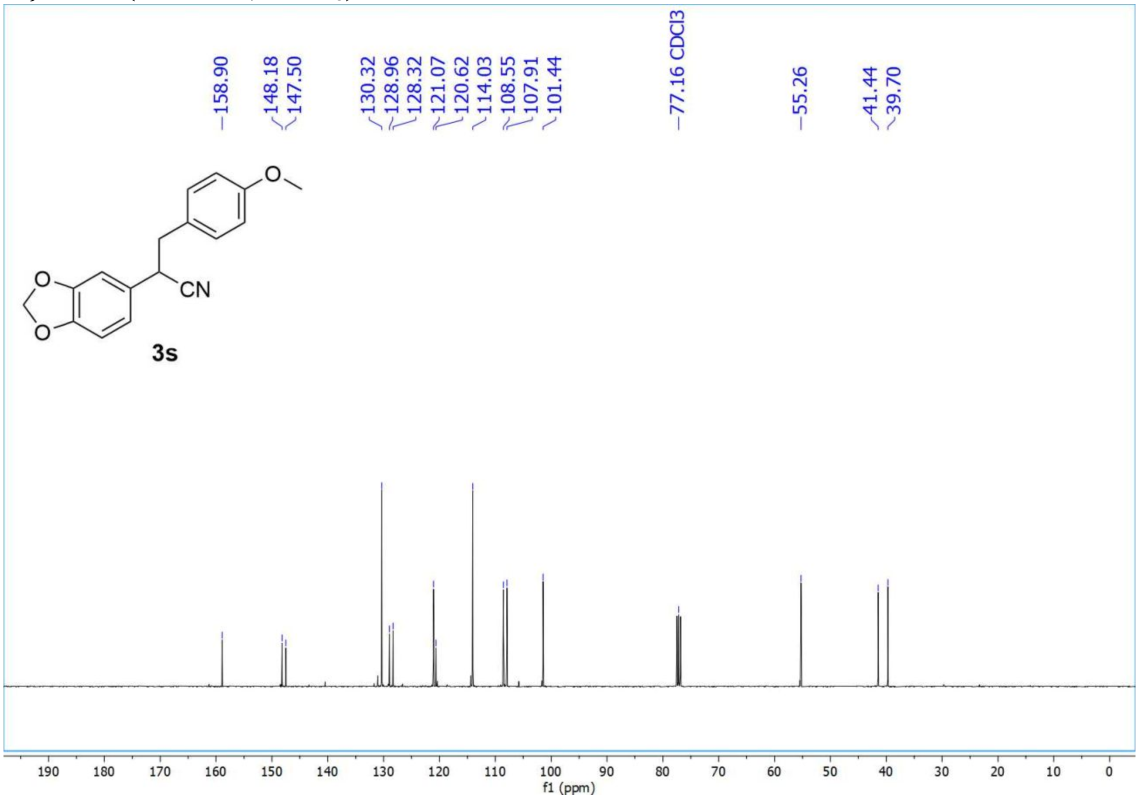

^{1}H NMR (400 MHz, $CDCl_3$) of 3t:

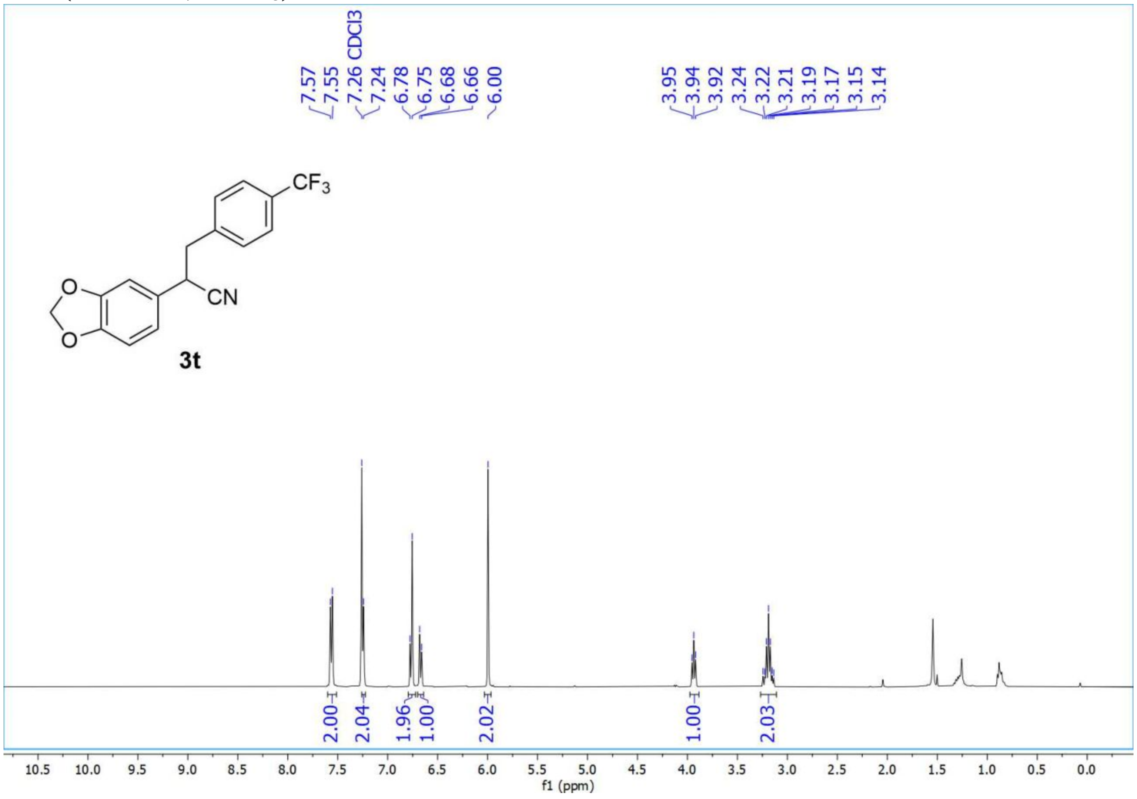

^{13}C{^{1}H} NMR (100 MHz, $CDCl_3$) of 3t:

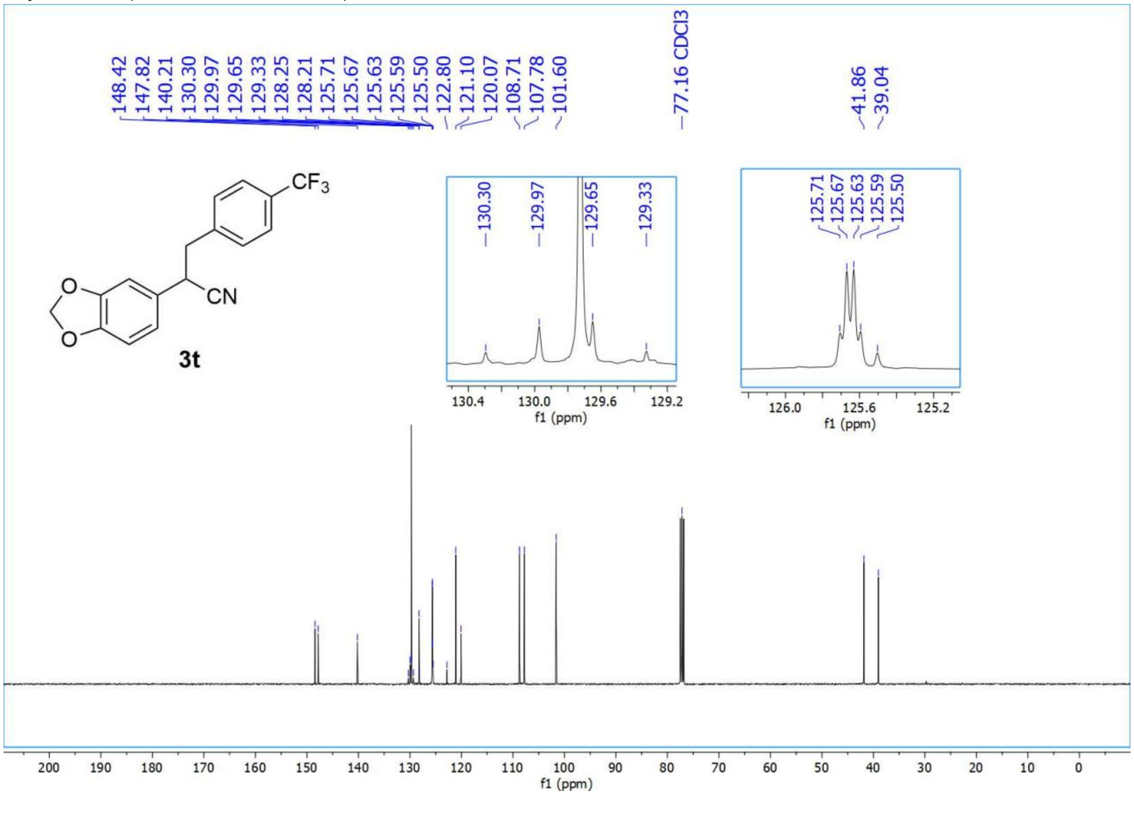

$^{19}F\{^1H\}$ NMR (376 MHz, $CDCl_3$) of 3t:

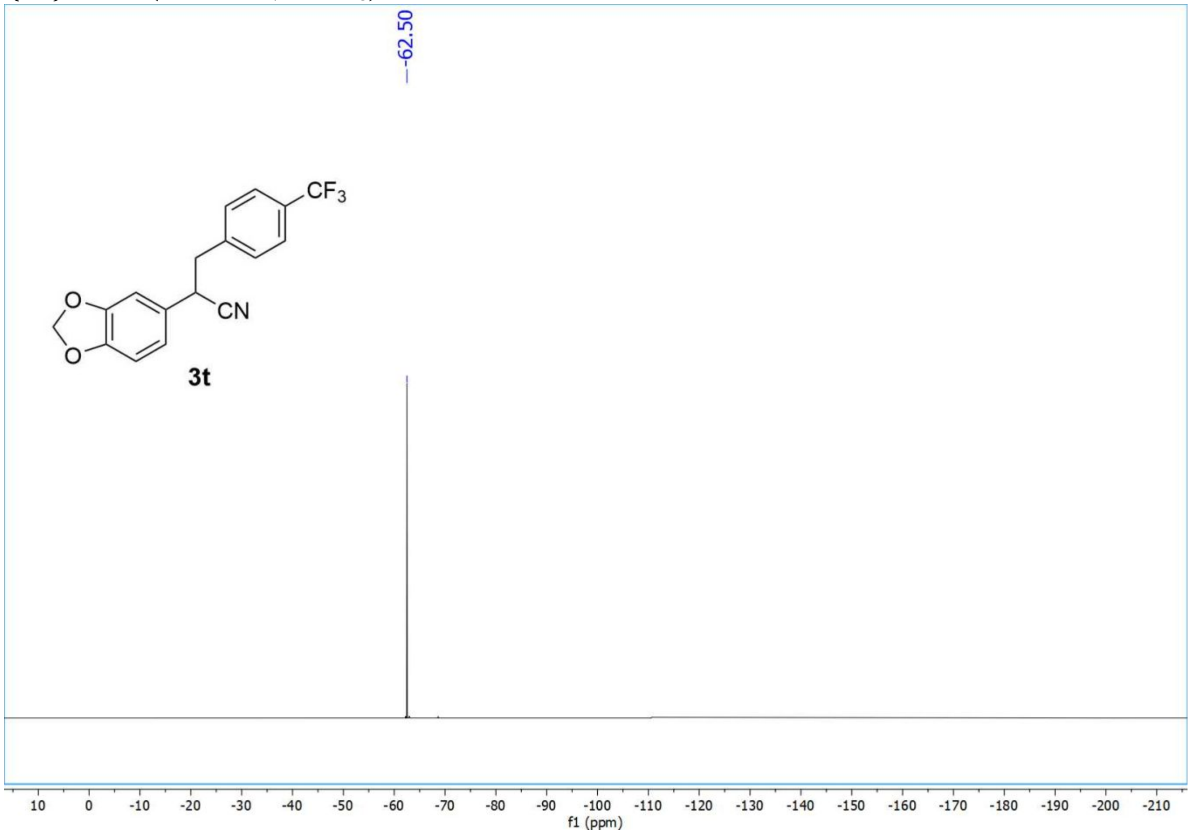

^{1}H NMR (500 MHz, $CDCl_3$) of 3u:

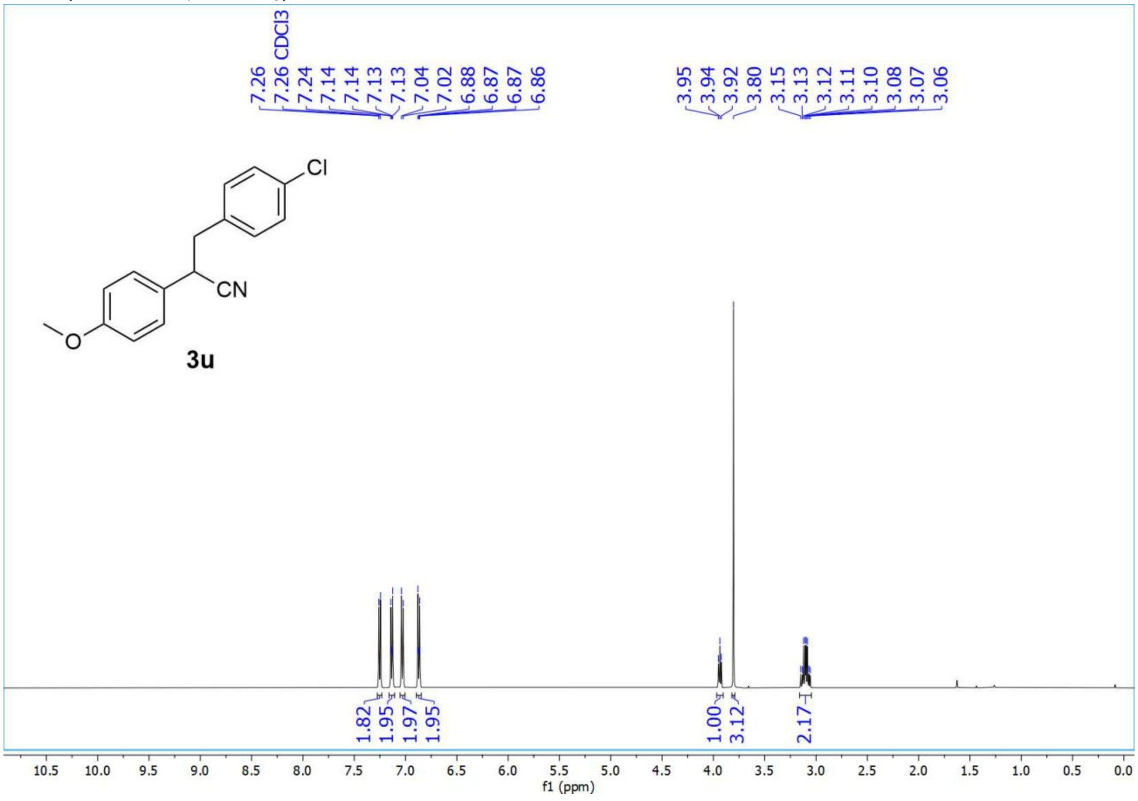

$^{13}C\{^{1}H\}$ NMR (125 MHz, $CDCl_3$) of 3u:

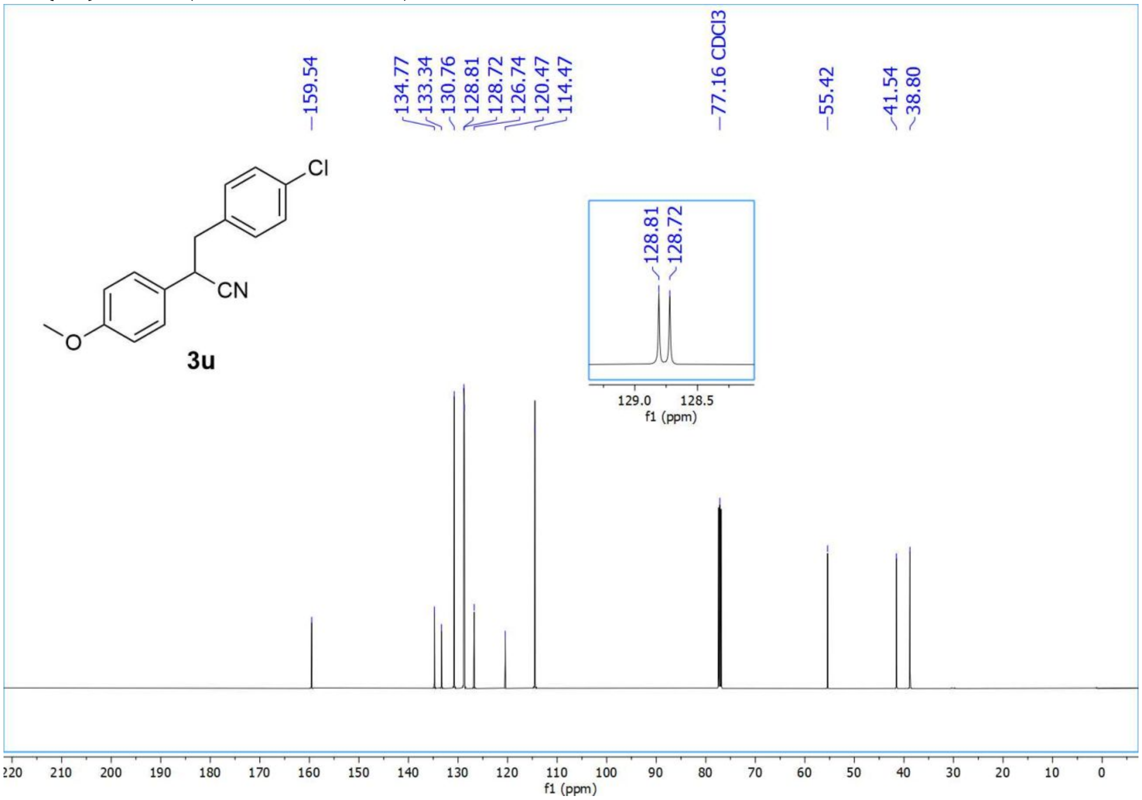

^{1}H NMR (400 MHz, $CDCl_3$) of 3v:

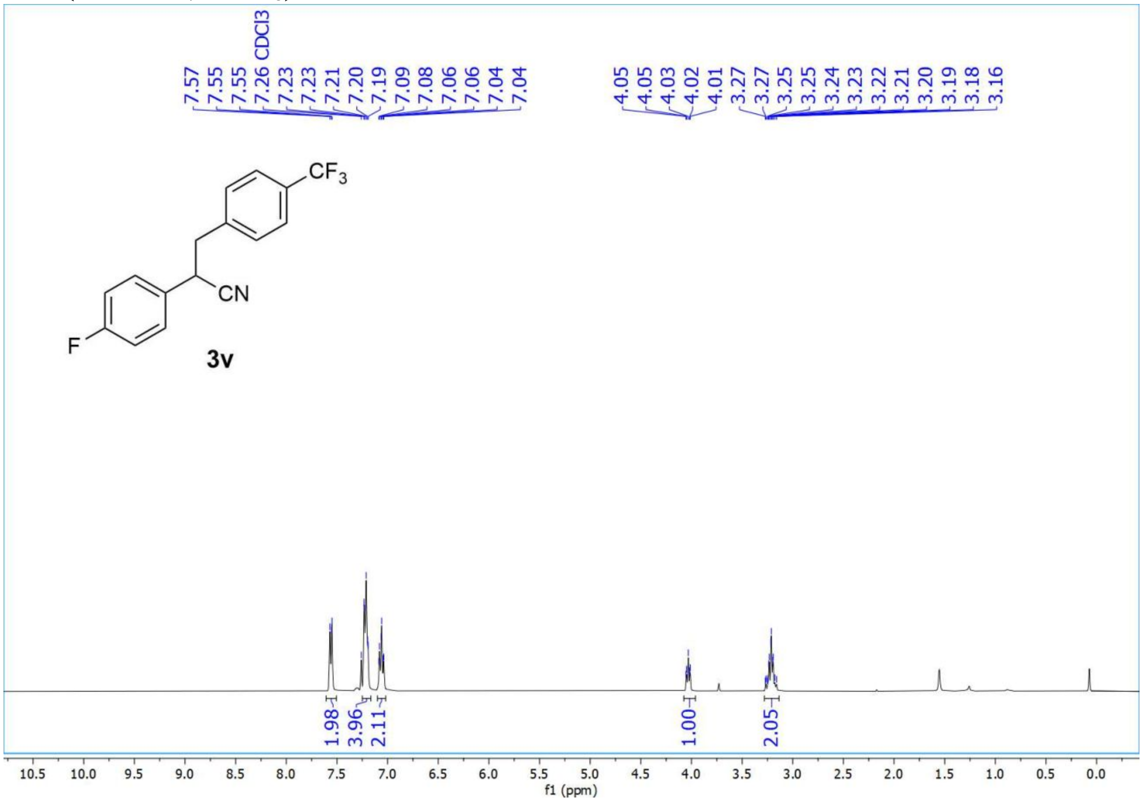

$^{13}C\{^{1}H\}$ NMR (100 MHz, $CDCl_3$) of 3v:

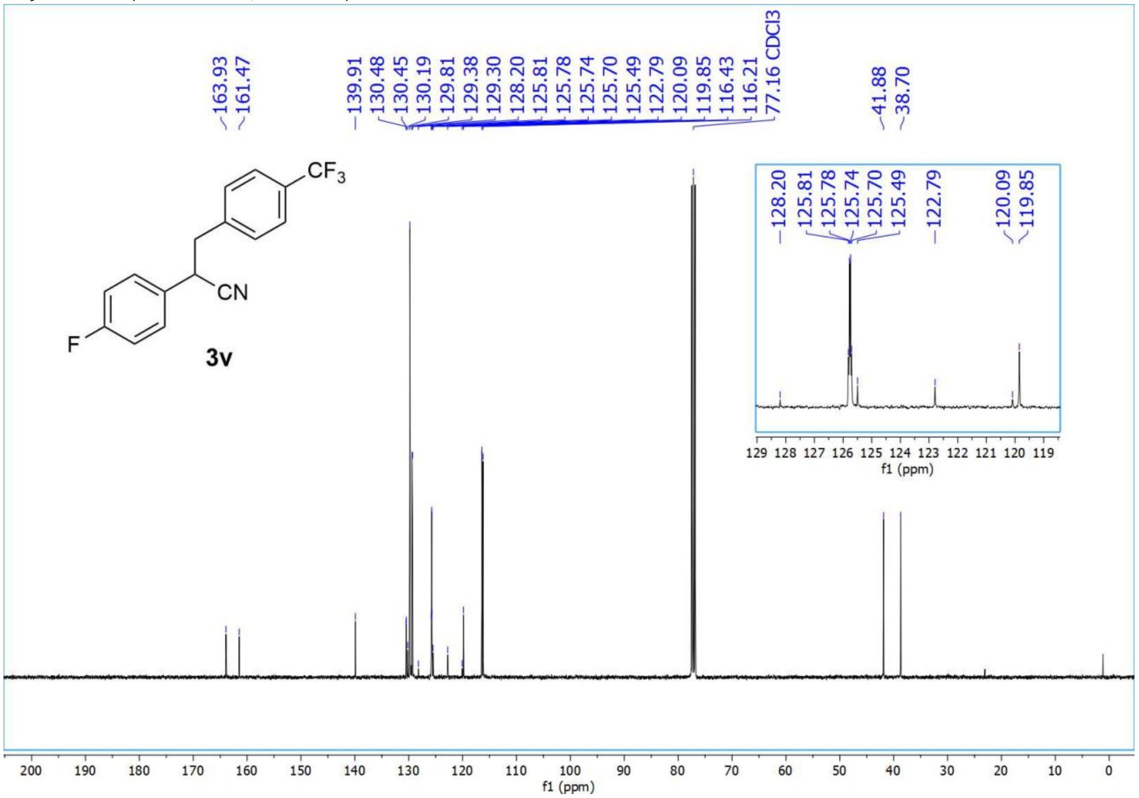

$^{19}F\{^1H\}$ NMR (376 MHz, $CDCl_3$) of 3v:

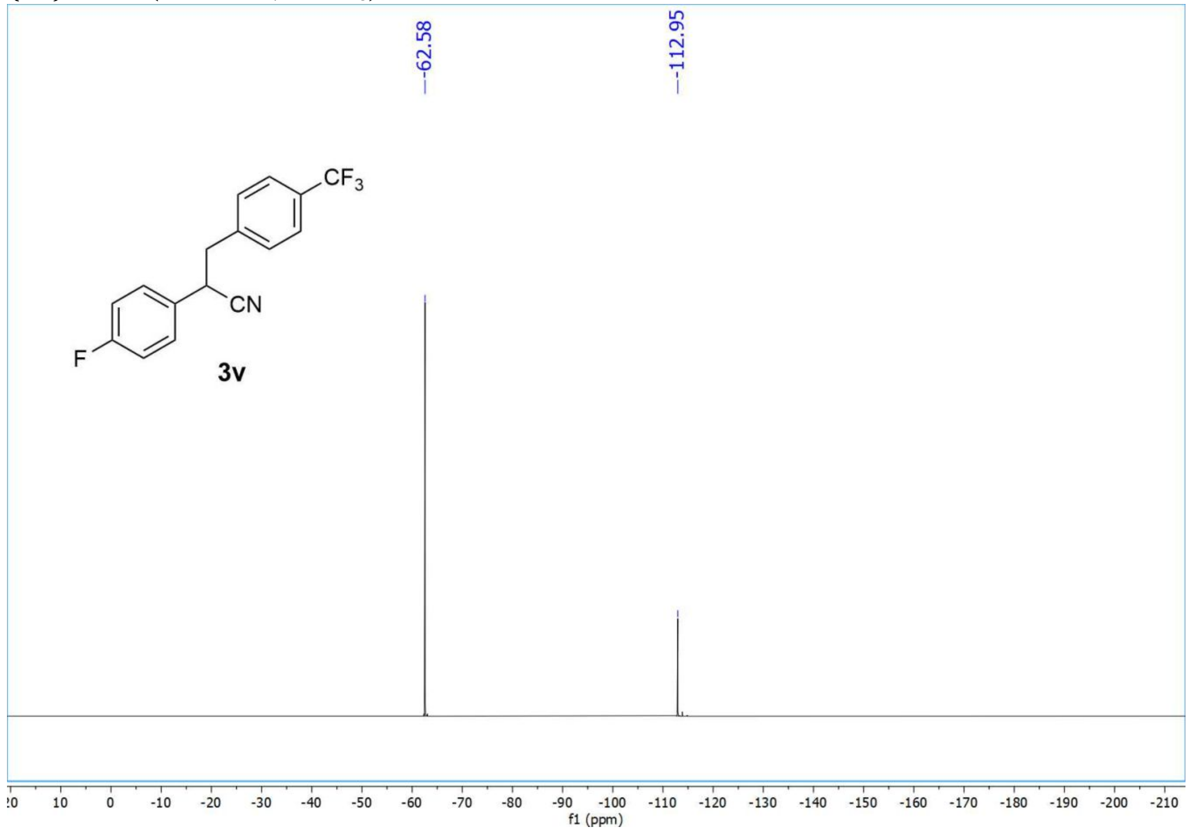

^{1}H NMR (400 MHz, CDCl$_3$) of 4q:

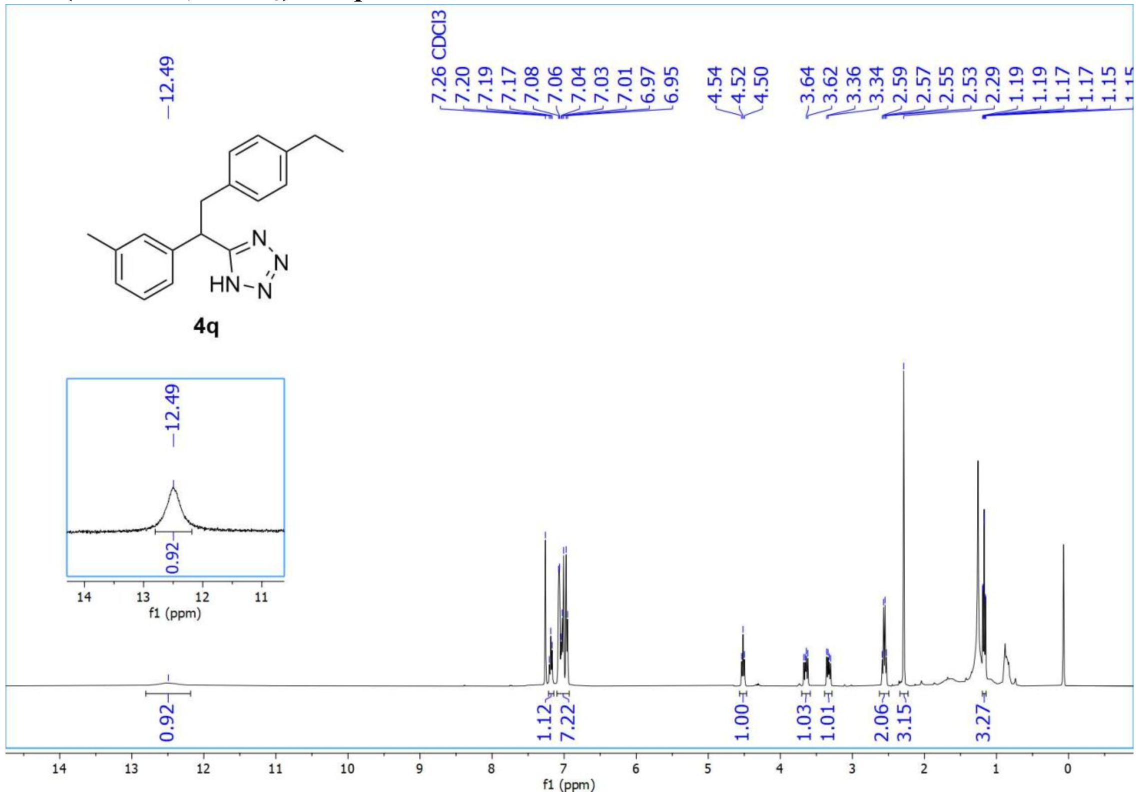

^{13}C{^{1}H} NMR (100 MHz, CDCl$_3$) of 4q:

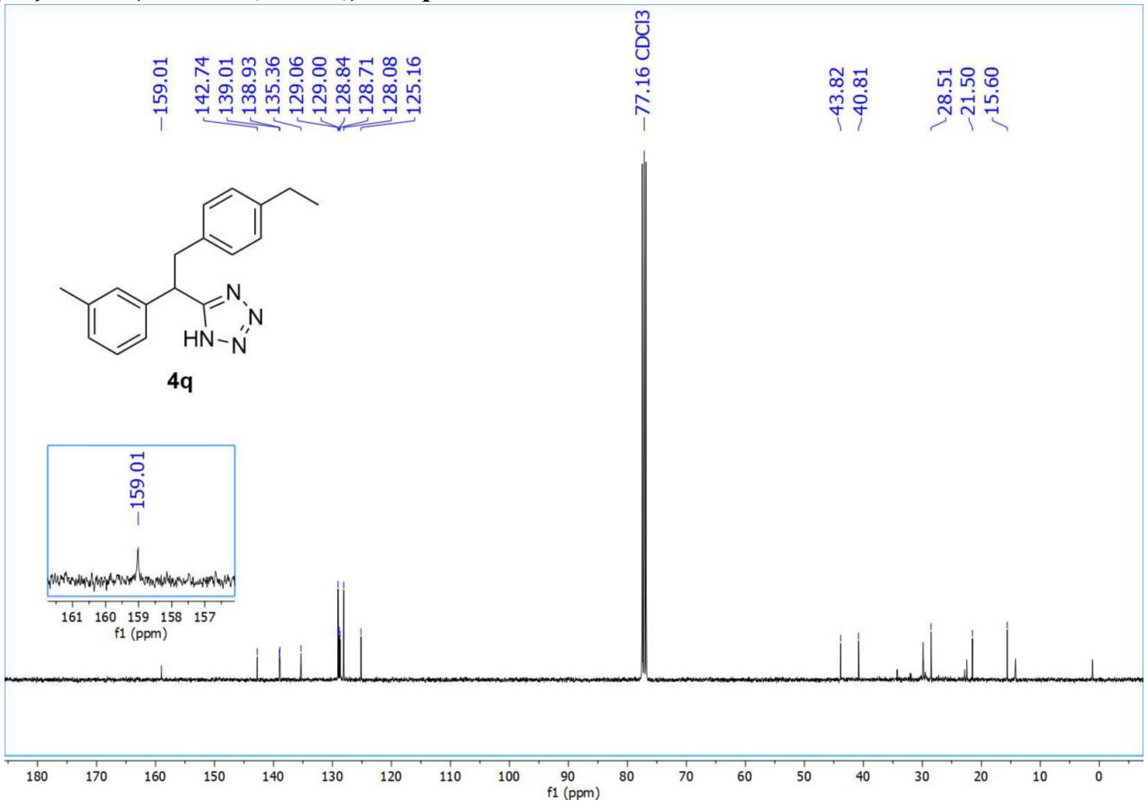

^{1}H NMR (400 MHz, $CDCl_3$) of 4n:

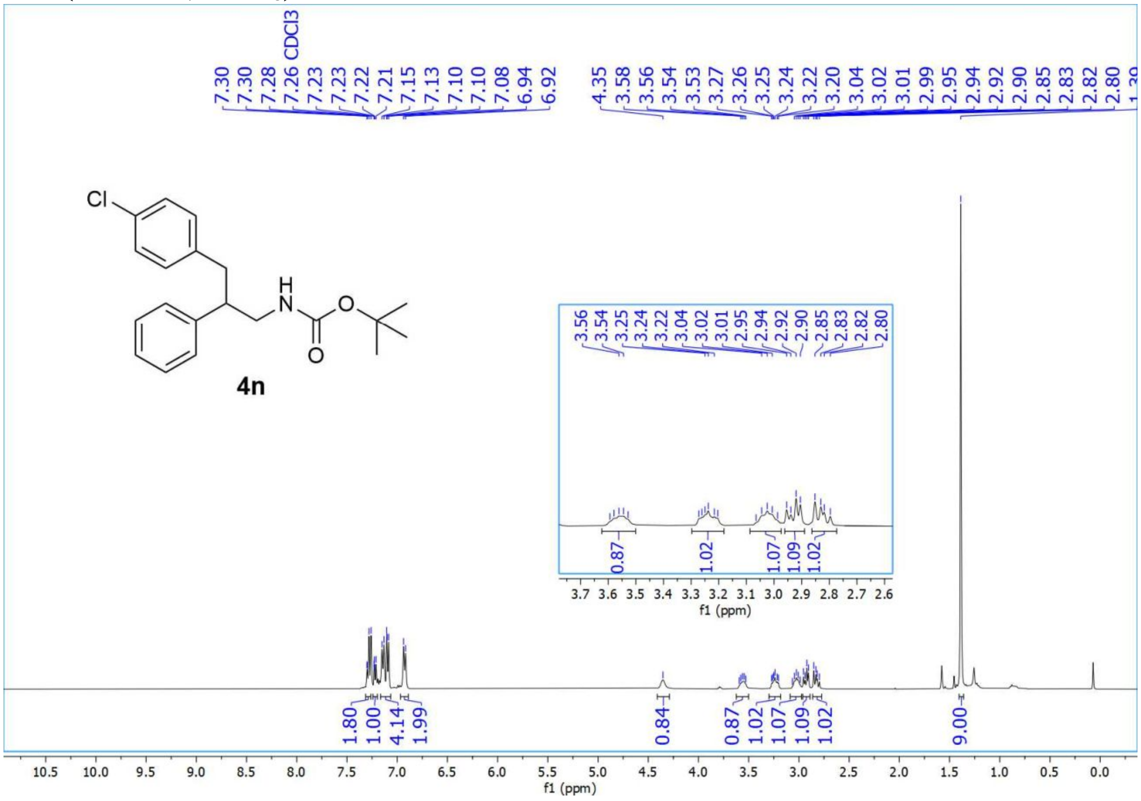

^{13}C{^{1}H} NMR (100 MHz, $CDCl_3$) of 4n:

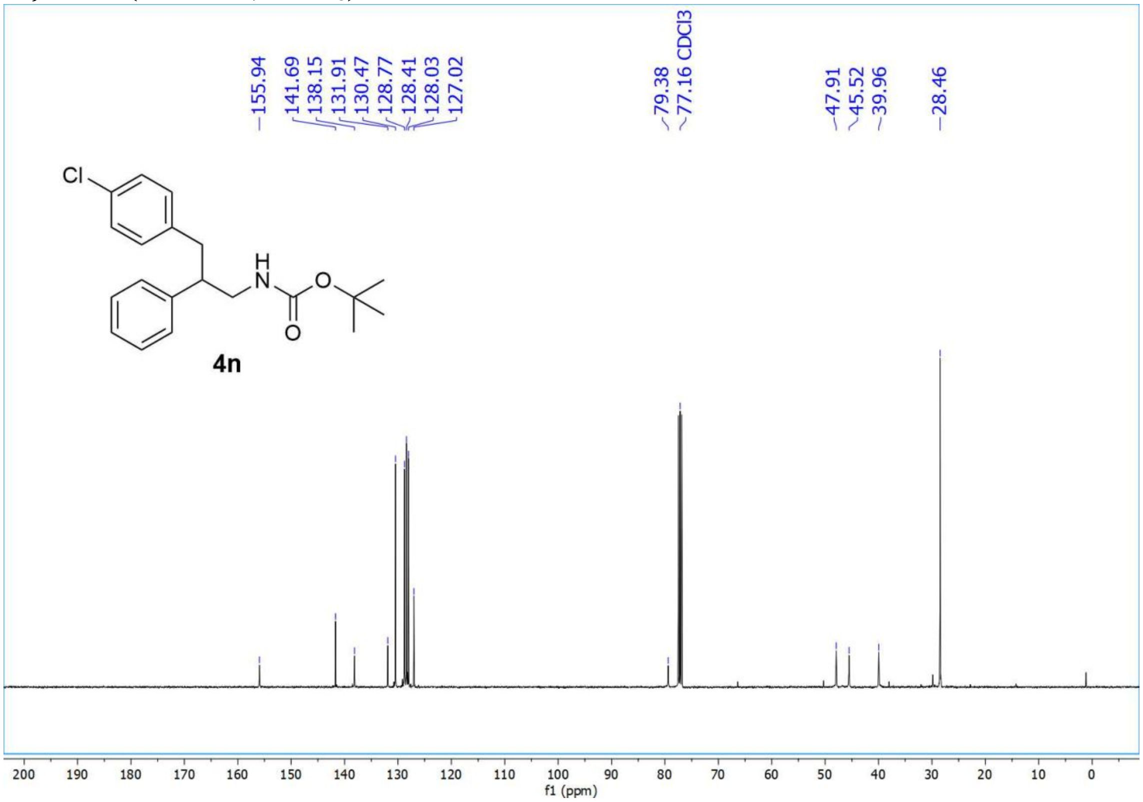

^{1}H NMR (400 MHz, DMSO-*d6*) of 4t:

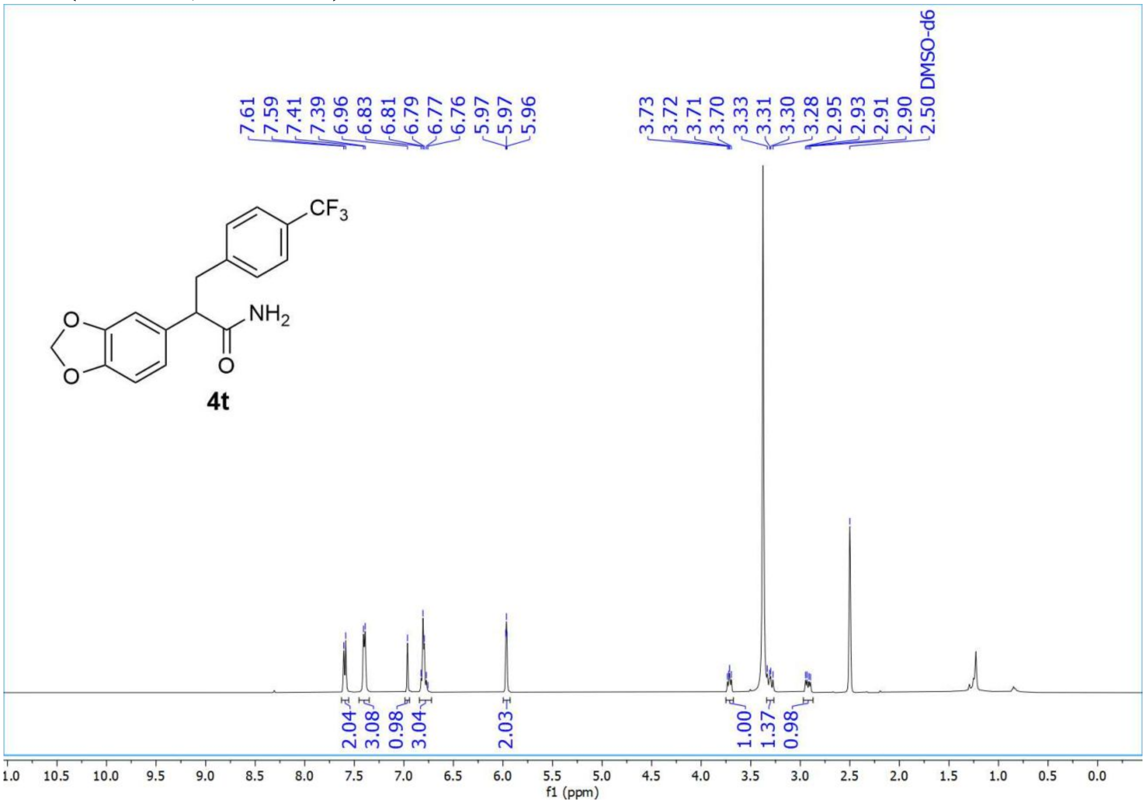

^{13}C{^{1}H} NMR (100 MHz, DMSO-*d6*) of 4t:

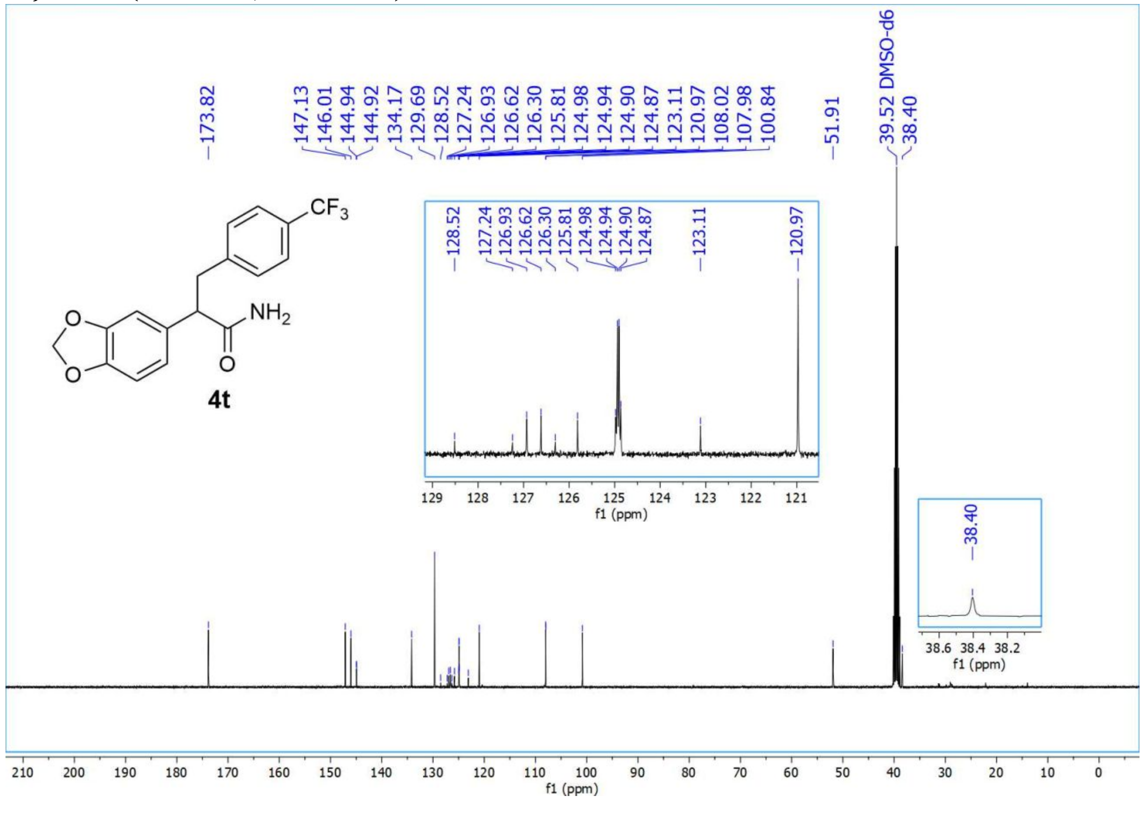

$^{19}F\{^{1}H\}$ NMR (376 MHz, DMSO-*d6*) of 4t:

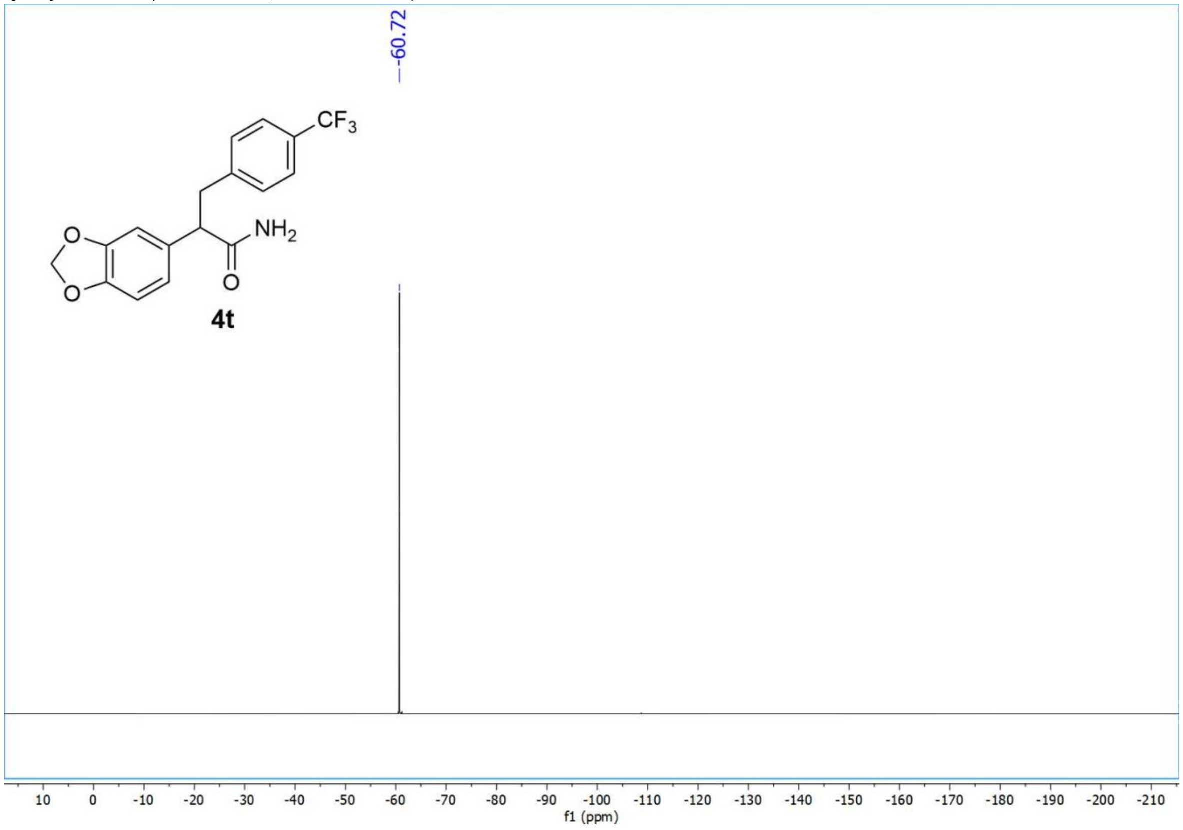

1. Computational Details[7]

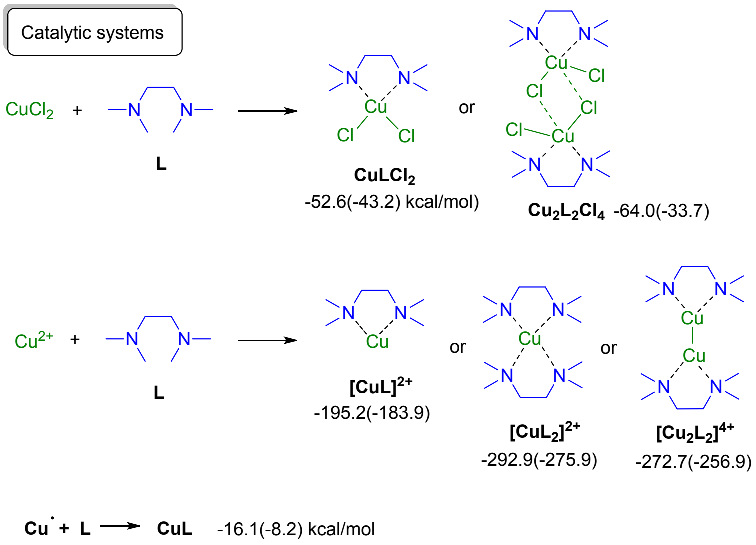

Scheme S1. Calculated catalytic systems and their reaction enthalpies (free reaction energies) of formation.

Table S7. Reaction energy ΔE (kcal/mol), Zero point energy reaction ΔE (kcal/mol), reaction enthalpies ΔH (kcal/mol), and free reaction energies ΔG (kcal/mol) at T = 298.15 K and P=1Atm via wB97XD/6-311G+(d,p) methodology in toluene solvent.

	ΔE	ΔE_0	ΔH	ΔG
$PhCH_2OH \rightarrow PhCH_2O- + H+$	284.49	275.29	276.32	270.08
$PhCH_2OH + tBuOK \rightarrow PhCH_2O^- + tBuOH + K^+$	54.09	52.88	52.89	47.25
$PhCH_2OH + tBuOK \rightarrow PhCH_2OK + tBuOH$	-4.15	-4.34	-4.94	-2.80
$PhCH_2OH + tBuOK \rightarrow PhCH_2OH\ldots tBuOK$	-21.69	-19.25	-19.53	-7.31
$PhCH_2OH\ldots tBuOK \rightarrow ts1$	2.47	-0.02	-0.52	0.37
$ts1 \rightarrow R1OK + tBuOH$	17.54	14.91	14.59	4.51
$PhCH_2OH \rightarrow PhCH_2O^\bullet + H^\bullet$	107.98	98.56	99.30	92.86
$PhCH_2OH \rightarrow PhCH_2O^- + H^+$	284.49	275.29	276.32	270.08
$tBuOH \rightarrow tBuO^\bullet + H^\bullet$	109.90	101.40	102.77	94.34
$tBuOH \rightarrow tBuO^- + H^+$	291.27	282.43	283.53	276.51
$PhCH_2OK \rightarrow PhCH_2O^\bullet + K^\bullet$	85.38	84.13	84.46	76.49
$PhCH_2OK \rightarrow PhCH_2O^- + K^+$	58.24	57.21	57.83	50.05
$tBuOK \rightarrow tBuO^\bullet + K^\bullet$	83.15	82.63	82.99	75.16
$tBuOK \rightarrow tBuO^- + K^+$	60.87	60.01	60.10	53.68
$tBuO^- + PhCH_2OH \rightarrow tBuOH + PhCH_2O^-$	-6.78	-7.14	-7.21	-6.43
$CuCl_2 + L \rightarrow CuLCl_2$	-55.56	-52.30	-52.55	-43.16
$2CuCl_2 + 2L \rightarrow Cu_2L_2Cl_4$	-70.38	-64.53	-64.02	-33.71
$Cu(II) + L \rightarrow [CuL]^{2+}$	-199.11	-193.78	-195.19	-183.90

2Cu(II) +2L $\rightarrow$ $[Cu_2L_2]^{2+}$	-278.16	-270.7	-272.7	-256.9
Cu(II) +2L $\rightarrow$ $[CuL_2]^{2+}$	-298.73	-290.7	-292.9	-275.9
Cu(II) +2L $\rightarrow$ $[CuL_2]^{2+}$ (b)	-281.82	-274.3	-276.3	-260.3
$CuCl_2$ $\rightarrow$ Cu(II) + $2Cl^-$	337.84	336.51	337.74	321.66
$CuCl_2$ $\rightarrow$ $Cu^\bullet$ + $2Cl^\bullet$	141.52	140.19	141.42	124.52
$CuLCl_2$ $\rightarrow$ $[CuL]^{2+}$ + $2Cl^-$	194.29	195.03	195.10	180.92
$CuLCl_2$ $\rightarrow$ CuL + $2Cl^\bullet$	180.06	176.63	177.87	159.52
$Cu^\bullet$ + L $\rightarrow$ CuL	-17.02	-15.87	-16.10	-8.16
2 $Cl^\bullet$ $\rightarrow$ Cl_2	-49.66	-48.85	-49.63	-42.86
CuL(22)+ $2Cl^-$ $\rightarrow$ CuL +Cl_2	-63.89	-67.25	-66.86	-64.26
$Cu_2L_2Cl_4$ $\rightarrow$ 2 $CuLCl_2$	-40.75	-40.07	-41.08	-52.61
$[CuL]^{2+}$ + L $\rightarrow$ $[CuL_2]^{2+}$	-99.63	-97.0	-97.7	-92.0
$[CuL_2]^{2+}$+ H $\rightarrow$ $[CuL_2H]^{2+}$	-62.49	-60.8	-61.3	-57.7
CuL + H$\rightarrow$ CuLH	-86.59	-82.85	-84.44	-75.13
$[CuL]^{2+}$+ H $\rightarrow$ $[CuHL]^{2+}$	-23.51	-19.13	-20.30	-12.27
$[CuL]^{2+}$ + H $\rightarrow$$[CuLH]^{2+}$	-89.46	-80.06	-81.14	-74.16
$CuLCl_2$ $\rightarrow$ CuL + $2Cl_2$	130.40	127.78	128.25	116.66
$PhCH_2O^-$ (**2b**) + $Cu^{II}L$ (**$[CuL]^{2+}$**) $\rightarrow$ **2b-CuL**	-170.1	-168.5	-168.5	-155.4
**2b** + $Cu^{II}LCl_2$ $\rightarrow$ **2b**-$Cu^{II}LCl_2$	-38.1	-36.3	-36.1	-24.7
**2b** + $Cu^{II}L_2$ $\rightarrow$ **2b**-$Cu^{II}L_2$	-89.6	-87.8	-87.5	-76.1
**2a** $\rightarrow$ H_2 + **5**	20.74	12.33	13.87	6.20
**5** + **1a** $\rightarrow$ H_2O + **3a'**	-1.10	-2.26	-0.55	-0.41
**1a** (RCH_2CN) $\rightarrow$ **10** ($RCHCN^-$) + H^+	271.74	262.64	264.60	255.76
**5** +H^+ $\rightarrow$ PhCHOH	-176.76	-167.79	-169.28	-161.38
**13** $\rightarrow$ OH^- + **3a'**	30.32	27.17	28.36	17.60
**13** + H^+ $\rightarrow$ H_2O + **3a'**	-262.59	-257.70	-257.69	-261.87
H^+ + OH^- $\rightarrow$ H_2O	-292.91	-284.87	-286.05	-279.47
**13** + tBuOH $\rightarrow$ $tBuO^-$ +H_2O + **3a'**	28.68	24.73	25.84	14.64
**1a** + $tButO^-$ $\rightarrow$ **10** ($PhCHCN^-$) + tBuOH	-19.53	-19.79	-18.93	-20.75
**5** + tButOH $\rightarrow$ **11** ($PhCHOH^+$) + $tBuO^-$	114.51	114.64	114.25	115.13
**10** + **11** $\rightarrow$ **12a** ([PhCH(OH)CH(CN)Ph])	-105.58	-102.53	-102.69	-90.28
**10** + **11** $\rightarrow$ **12b** ([PhCH(OH)CH(CN)Ph])	-105.31	-102.30	-102.45	-89.87
$tBuO^-$ + **12a** $\rightarrow$ tBuOH + **13**($[PhCH(OH)C(CN)Ph]^-$)	-19.18	-19.30	-19.01	-19.15
**14 + 2b** $\rightarrow$ **3a + 5**	-135.64	-134.18	-134.06	-135.35
**15 + 2b** $\rightarrow$ **3a + 5**	-135.96	-134.35	-134.24	-135.27
**3a'** + tBuOH $\rightarrow$ **14** + $tBuO^-$	128.83	127.63	127.36	128.32
**2a** + **3a'** $\rightarrow$ **14 + 2b**	122.05	120.50	120.15	121.89
**2a** + **3a'** $\rightarrow$ **15 + 2b**	122.37	120.67	120.33	121.81
**3a'** + H^+ $\rightarrow$ **14**	-162.44	-154.80	-156.17	-148.19
**3a'** + H^+ $\rightarrow$ **15**	-162.11	-154.63	-155.98	-148.27
**2a** + **3a'** $\rightarrow$ **5** + **3a**	-13.59	-13.68	-13.91	-13.46

Table S8. Relative reaction energy ΔE (kcal/mol), Zero point energy reaction ΔE (kcal/mol), reaction enthalpies ΔH (kcal/mol), and free reaction energies ΔG (kcal/mol) at T = 298.15 K and P=1Atm via wB97XD/6-311G+(d,p) methodology in toluene solvent for the reaction involved in catalytic cycles **B** and **C** of Scheme 6.

	**ΔE**	**$ΔE_0$**	**ΔH**	**ΔG**
**1a** ($PhCH_2CN$) + $tButO^-$	0.00	0.00	0.00	0.00
**1a**-$tBuO^-$ (dimer)	-16.05	-15.10	-14.40	-4.10
**ts16**	-15.76	-15.87	-15.77	-3.86
**10** ($PhCHCN^-$) + tBuOH	-19.53	-19.79	-18.93	-20.75
**5** ($PhCH_2OH$) + tBuOH	0.00	0.00	0.00	0.00
**5**-tBuOH (dimer)	-5.85	-4.82	-4.30	4.16
**ts17**	-3.56	-2.96	-3.48	8.28
**11** ($R1CHOH^+$) + $tBuO^-$	114.51	114.64	114.25	115.13
**10** + **11**	0.00	0.00	0.00	0.00
**ts18a**	-39.19	-38.34	-39.03	-24.53
**12a** (PhCH(CN)CH(OH)Ph)	-105.58	-102.53	-102.69	-90.28
**ts18b**	-49.28	-48.43	-49.13	-34.62
**12b** (PhCH(CN)CH(OH)Ph)	-105.31	-102.30	-102.45	-89.87
**12a** + $tBuO^-$	0.00	0.00	0.00	0.00
12a- tBuO (dimer)	-20.30	-6.73	-20.09	-7.28
**ts19**	-16.13	-2.56	-15.93	-3.11
**13**-tBuOH (dimer)	-32.03	-30.17	-29.90	-17.04
**13** $[PhC(CN)CH(OH)Ph]^-$ + tBuOH	-19.18	-19.30	-19.01	-19.15
**13**	0.0	0.0	0.0	0.0
**ts20**	20.7	19.0	19.2	18.9
**3a'** [PhC(CN)CHPh] + OH^-	30.3	27.2	28.4	17.6
**2a+3a'**	0.00	0.00	0.00	0.00
**D1** 2a..3a' (dimer)	-6.83	-5.88	-5.92	7.10
**ts22a**	51.99	49.14	48.95	62.98
**ts22b**	53.37	50.69	50.46	64.29
**14 + 2b**	122.05	120.50	120.15	121.89
**3a'** + tBuOH	0.00	0.00	0.00	0.00
**3a'…**tBuOH **(dimer)**	-7.54	-6.52	-5.95	3.91
**ts25**	55.24	52.28	52.24	64.50
**ts26**	71.61	68.27	68.02	80.69

**ts27**	66.60	64.59	64.32	77.42
**14 + tBuO-**	128.83	127.63	127.36	128.32
**14+2b**	0.00	0.00	0.00	0.00
**D2 (14…2b)** (dimer)	-77.22	-76.56	-76.85	-63.79
**3a+5**	-135.64	-134.18	-134.06	-135.35
**D3 (15…2b)** (dimer)	-75.85	-75.41	-75.67	-63.17
**15+2b**	0.00	0.00	0.00	0.00
**ts24**	-92.18	-93.63	-94.03	-80.19
**15+2b** (dimer)	-143.46	-141.35	-141.61	-129.07
**3a+5**	-135.96	-134.35	-134.24	-135.27
**14**	0.00	0.00	0.00	0.00
**ts23** (ts_14-15)	20.28	17.78	17.64	17.91
**15**	0.33	0.17	0.18	-0.08

Table S9. Geometry of the **2a**, **2a'**, **2b** and **5** molecules in toluene solvent and attached or linked at the CuL_xCl_y complexes.

	**$PhCH_2O^-$ (2b)**				**$PhCHO^-$ (2a')**				**PhCHO (5)**			
	C-C[a]	C-O	CCO	Cu-O	C-C[a]	C-O	CCO	Cu-O	C-C[a]	C-O	CCO	Cu-O
toluene	1.541	1.338	116.0		1.466	1.266	126.7		1.479	1.248	120.8	
-CuL	1.519	1.404	108.9	1.822	1.414	1.304	125.6	1.954	1.486	1.207	123.9	3.985[b]
-CuL_2	1.521	1.377	126.7	1.900	1.407	1.343	120.3	3.700				
-$CuLCl_2$	1.513	1.399	126.2	1.835	1.466	1.221	124.8	2.540	1.473	1.213	124.5	3.369
-Cu_2L_2	1.498	1.460	111.8	1.835<br>1.864	1.421	1.270	125.4	1.938				

	**$PhCH_2OH$ (2a)**				**$PhCH_2O^\bullet$**			
	C-C[a]	C-O	CCO	Cu-O	C-C[a]	C-O	CCO	Cu-O
toluene	1.508	1.415	110.2		1.514	1.350	117.5	
-CuL	1.487	1.498	107.3	1.887				

[a] C-C distance between the C of the Ph group and the C of the -CH_2OH, -CH_2O^-, -CHO^- and -CHO groups.
[b] Cu…C = 3.353 Å.

Table S10. Geometries optimized at wB97XD/6-311G+(d,p) methodology in toluene solvent.

$PhCH_2OH$ (2a)

Center Number	Atomic Number	Atomic Type	Coordinates (Angstroms) X	Y	Z
1	6	0	-0.626732	0.427813	0.039196
2	6	0	0.764781	0.438518	0.068668
3	6	0	1.452182	1.643911	0.013903
4	6	0	0.761608	2.853746	-0.062632
5	6	0	-0.629573	2.837811	-0.093771
6	6	0	-1.319058	1.629521	-0.044210
7	1	0	-1.165711	-0.512214	0.076989
8	1	0	1.315188	-0.493997	0.127683
9	1	0	2.538405	1.644937	0.028977
10	1	0	-1.168374	3.774576	-0.162365
11	1	0	-2.403273	1.629790	-0.072425
12	6	0	1.538734	4.146526	-0.079990
13	1	0	1.928811	4.338443	0.929006
14	1	0	2.399856	4.038571	-0.752097
15	8	0	0.706108	5.211873	-0.497817
16	1	0	1.179670	6.034988	-0.375932

$PhCH_2OK$

Center Number	Atomic Number	Atomic Type	Coordinates (Angstroms) X	Y	Z
1	6	0	2.196495	-0.442113	0.063999
2	6	0	1.910577	0.916491	-0.063432
3	6	0	0.592059	1.351235	-0.085318
4	6	0	-0.468922	0.447445	0.018724
5	6	0	-0.171017	-0.904881	0.145174
6	6	0	1.149539	-1.349967	0.167994
7	1	0	3.225372	-0.785336	0.081558
8	1	0	2.719709	1.635236	-0.145600
9	1	0	0.376178	2.412652	-0.184873
10	1	0	-1.000839	-1.598557	0.225344
11	1	0	1.362084	-2.410091	0.267536
12	6	0	-1.917890	0.940541	-0.006740
13	1	0	-1.980315	1.708515	0.797743
14	1	0	-2.011581	1.526007	-0.949813
15	8	0	-2.866713	-0.023205	0.110705
16	19	0	-4.502176	-1.651925	0.334706

$PhCH_2O^-$ (2b)

Center Number	Atomic Number	Atomic Type	Coordinates (Angstroms) X	Y	Z
1	6	0	2.263280	-0.300547	0.000074
2	6	0	1.810445	1.018936	0.000135
3	6	0	0.446607	1.285160	0.000049
4	6	0	-0.494820	0.252050	-0.000113
5	6	0	-0.031003	-1.060100	-0.000136
6	6	0	1.335212	-1.336805	-0.000068
7	1	0	3.327476	-0.514657	0.000114
8	1	0	2.524681	1.837393	0.000252
9	1	0	0.097271	2.316435	0.000139
10	1	0	-0.779395	-1.846286	-0.000305
11	1	0	1.678139	-2.367829	-0.000137
12	6	0	-2.008799	0.538678	-0.000307
13	1	0	-2.148854	1.228287	0.879749
14	1	0	-2.148799	1.226899	-0.881465
15	8	0	-2.809506	-0.533060	0.000481

$PhCH_2O^{\bullet}$

Center Number	Atomic Number	Atomic Type	Coordinates (Angstroms) X	Y	Z
1	6	0	2.259151	0.276677	-0.000099
2	6	0	1.792219	-1.034188	-0.000147
3	6	0	0.425675	-1.284333	-0.000051
4	6	0	-0.485752	-0.229392	0.000103
5	6	0	-0.015644	1.079326	0.000154
6	6	0	1.352718	1.330761	0.000051
7	1	0	3.325079	0.474640	-0.000184
8	1	0	2.493005	-1.861551	-0.000274
9	1	0	0.065052	-2.309230	-0.000101
10	1	0	-0.725754	1.898090	0.000258
11	1	0	1.711209	2.354172	0.000084
12	6	0	-1.968922	-0.530952	0.000263
13	1	0	-2.238575	-1.176933	-0.860432
14	1	0	-2.238591	-1.175635	0.861946
15	8	0	-2.818512	0.518632	-0.000369

$PhCHO^-$ (2a')

Center Number	Atomic Number	Atomic Type	Coordinates (Angstroms) X	Y	Z
1	6	0	-0.633456	0.396932	0.058845
2	6	0	0.774348	0.441699	0.010224

3	6	0	1.442854	1.641172	-0.091524
4	6	0	0.745831	2.888862	-0.152249
5	6	0	-0.685208	2.822051	-0.101454
6	6	0	-1.338299	1.610025	0.000674
7	1	0	-1.157851	-0.548973	0.139120
8	1	0	1.343208	-0.483860	0.053525
9	1	0	2.530329	1.651049	-0.127499
10	1	0	-1.246578	3.749551	-0.145245
11	1	0	-2.425375	1.595479	0.036838
12	6	0	1.446362	4.118799	-0.257050
13	1	0	2.552916	4.018760	-0.285814
14	8	0	0.936721	5.275894	-0.318222

tBuOH

Center Number	Atomic Number	Atomic Type	Coordinates (Angstroms) X	Y	Z
1	6	0	-0.006677	0.000020	0.016690
2	6	0	0.672557	-1.259226	-0.523370
3	1	0	0.197442	-2.152496	-0.110642
4	1	0	0.606718	-1.301203	-1.614083
5	1	0	1.733132	-1.274837	-0.250907
6	6	0	-1.491486	0.002076	-0.324305
7	1	0	-1.977358	-0.883779	0.091803
8	1	0	-1.975080	0.889275	0.091598
9	1	0	-1.635327	0.002142	-1.407713
10	6	0	0.676318	1.256875	-0.524122
11	1	0	0.203082	2.151838	-0.112883
12	1	0	1.736658	1.270067	-0.250596
13	1	0	0.611712	1.297780	-1.614945
14	8	0	0.052492	0.000440	1.449352
15	1	0	0.974818	-0.000778	1.714203

tBuOK

Center Number	Atomic Number	Atomic Type	Coordinates (Angstroms) X	Y	Z
1	6	0	-1.331411	0.828693	-0.183115
2	6	0	-2.855449	1.038303	-0.047680
3	1	0	-3.244704	0.398040	0.751167
4	1	0	-3.121717	2.077706	0.181232
5	1	0	-3.349186	0.752500	-0.982459
6	6	0	-0.664758	1.243822	1.146698
7	1	0	-1.029768	0.602031	1.955808
8	1	0	0.419356	1.109038	1.070241
9	1	0	-0.866359	2.288168	1.415266
10	6	0	-0.811621	1.761389	-1.298971

11	1	0	0.271763	1.640054	-1.404481
12	1	0	-1.277938	1.489232	-2.251935
13	1	0	-1.023167	2.819303	-1.099984
14	8	0	-1.047613	-0.481457	-0.476487
15	19	0	-0.569243	-2.689850	-0.970996

PhCH$_2$OH… tBuOK

Center Number	Atomic Number	Atomic Type	Coordinates (Angstroms) X	Y	Z
1	6	0	-3.349431	0.456580	-0.255542
2	6	0	-3.047482	1.672436	-1.152328
3	1	0	-3.186183	1.400449	-2.203364
4	1	0	-3.694444	2.527518	-0.925798
5	1	0	-2.006858	1.986160	-1.018462
6	6	0	-4.815080	0.038875	-0.455449
7	1	0	-4.979652	-0.249744	-1.498486
8	1	0	-5.044367	-0.823555	0.178935
9	1	0	-5.513467	0.845763	-0.206362
10	6	0	-3.142000	0.860457	1.216442
11	1	0	-3.351075	0.006507	1.868068
12	1	0	-2.102070	1.162660	1.378287
13	1	0	-3.790349	1.692324	1.514451
14	8	0	-2.515134	-0.600281	-0.585330
15	19	0	-1.430716	-2.681670	-1.241990
16	6	0	4.876482	0.106610	0.304412
17	6	0	4.301120	1.367941	0.439461
18	6	0	2.930700	1.527825	0.287313
19	6	0	2.109575	0.435217	0.000888
20	6	0	2.691960	-0.820513	-0.133684
21	6	0	4.067052	-0.984845	0.016814
22	1	0	5.946867	-0.020842	0.421999
23	1	0	4.923405	2.227929	0.662418
24	1	0	2.488831	2.515001	0.392067
25	1	0	2.056444	-1.668506	-0.356923
26	1	0	4.506328	-1.971058	-0.091216
27	6	0	0.619178	0.642435	-0.145030
28	1	0	0.232960	1.019207	0.815871
29	1	0	0.456524	1.446944	-0.880435
30	8	0	-0.044418	-0.523287	-0.522337
31	1	0	-1.081759	-0.377708	-0.493104

2a'-CuLH

Center Number	Atomic Number	Atomic Type	Coordinates (Angstroms) X	Y	Z

1	6	0	-2.341434	1.438483	-0.327642
2	6	0	-1.016908	2.180530	-0.213428
3	1	0	-3.101108	2.165105	-0.008126
4	1	0	-2.562776	1.196574	-1.368094
5	1	0	-0.767734	2.398287	0.825148
6	1	0	-1.136426	3.135079	-0.729451
7	7	0	-2.511262	0.201617	0.477675
8	7	0	0.202369	1.537967	-0.806834
9	6	0	0.034230	1.084677	-2.214722
10	1	0	-0.257198	1.936384	-2.829149
11	1	0	0.984169	0.676241	-2.552686
12	1	0	-0.721024	0.302625	-2.259992
13	6	0	1.371427	2.459342	-0.681266
14	1	0	2.261383	1.927787	-1.012959
15	1	0	1.192125	3.339416	-1.297259
16	1	0	1.486934	2.737226	0.364007
17	6	0	-3.893379	-0.293300	0.242291
18	1	0	-4.050197	-1.211393	0.807327
19	1	0	-4.625794	0.455204	0.565633
20	1	0	-4.036240	-0.500940	-0.818340
21	6	0	-2.353878	0.492093	1.922616
22	1	0	-3.017587	1.311192	2.225515
23	1	0	-2.609458	-0.400334	2.492610
24	1	0	-1.321355	0.754776	2.152788
25	29	0	-1.279372	-1.213924	-0.066055
26	1	0	0.452152	0.712229	-0.233741
27	6	0	3.529909	0.666299	1.432892
28	6	0	4.043278	0.127303	0.239891
29	6	0	3.304647	-0.755306	-0.515174
30	6	0	1.995125	-1.156890	-0.117263
31	6	0	1.501816	-0.616363	1.109245
32	6	0	2.264634	0.274411	1.858235
33	1	0	4.123706	1.351013	2.025782
34	1	0	5.038740	0.405253	-0.089071
35	1	0	3.722207	-1.168864	-1.428086
36	1	0	0.573850	-0.996743	1.526111
37	1	0	1.874963	0.640871	2.802860
38	6	0	1.242681	-2.035553	-0.930156
39	1	0	1.743011	-2.509051	-1.777802
40	8	0	-0.009398	-2.353197	-0.749554

2a'_HCuL

Center Number	Atomic Number	Atomic Type	Coordinates (Angstroms) X	Y	Z
1	6	0	-2.397228	1.388356	-0.318998
2	6	0	-1.100901	2.187423	-0.298569
3	1	0	-3.169030	2.096916	0.012399

4	1	0	-2.661919	1.096032	-1.335993
5	1	0	-0.843410	2.497311	0.714906
6	1	0	-1.266311	3.090937	-0.888097
7	7	0	-2.475377	0.173173	0.531261
8	7	0	0.126600	1.535064	-0.865335
9	6	0	-0.015343	1.063979	-2.287262
10	1	0	0.685416	1.612875	-2.910967
11	1	0	0.211719	0.000126	-2.328666
12	1	0	-1.026470	1.243061	-2.642308
13	6	0	1.306401	2.439696	-0.707304
14	1	0	2.194775	1.895581	-1.023962
15	1	0	1.152673	3.326999	-1.320258
16	1	0	1.405081	2.708870	0.341802
17	6	0	-3.861704	-0.352935	0.420901
18	1	0	-3.953141	-1.263575	1.011515
19	1	0	-4.580028	0.387593	0.791794
20	1	0	-4.086778	-0.581370	-0.620731
21	6	0	-2.210367	0.507363	1.949971
22	1	0	-2.861711	1.326376	2.278555
23	1	0	-2.407229	-0.370191	2.564469
24	1	0	-1.167989	0.792464	2.092157
25	29	0	-1.253555	-1.222600	-0.091853
26	1	0	0.351384	0.713643	-0.279577
27	6	0	3.492515	0.707851	1.471615
28	6	0	4.041813	0.165688	0.296581
29	6	0	3.330361	-0.727819	-0.471653
30	6	0	2.012476	-1.134303	-0.108941
31	6	0	1.483413	-0.592027	1.101078
32	6	0	2.220293	0.306935	1.865402
33	1	0	4.064207	1.400866	2.076489
34	1	0	5.043668	0.449358	-0.006979
35	1	0	3.775001	-1.142257	-1.371169
36	1	0	0.550339	-0.979136	1.500999
37	1	0	1.803574	0.674032	2.798309
38	6	0	1.289182	-2.020114	-0.941673
39	1	0	1.820236	-2.502312	-1.764975
40	8	0	0.026877	-2.327127	-0.814294

2b-CuL

Center Number	Atomic Number	Atomic Type	Coordinates (Angstroms) X	Y	Z
1	6	0	2.665032	0.842330	0.837930
2	6	0	2.102090	1.857298	-0.143219
3	1	0	3.756813	0.914880	0.873924
4	1	0	2.294251	1.035496	1.845502
5	1	0	2.538645	1.707128	-1.133183
6	1	0	2.343802	2.877007	0.174493
7	7	0	2.241929	-0.519357	0.443512

8	7	0	0.628378	1.695781	-0.271103
9	6	0	-0.064871	2.277850	0.899978
10	1	0	0.175939	3.342754	0.987395
11	1	0	-1.139437	2.160985	0.773121
12	1	0	0.228054	1.768636	1.818026
13	6	0	0.150282	2.375126	-1.496199
14	1	0	-0.930300	2.255000	-1.574389
15	1	0	0.390898	3.443102	-1.459638
16	1	0	0.623071	1.933383	-2.374064
17	6	0	2.154692	-1.429102	1.600801
18	1	0	1.814154	-2.402648	1.250564
19	1	0	3.132464	-1.531740	2.084625
20	1	0	1.433979	-1.038558	2.321456
21	6	0	3.131702	-1.097134	-0.583009
22	1	0	4.124579	-1.284181	-0.159174
23	1	0	2.695897	-2.030115	-0.938012
24	1	0	3.228148	-0.411505	-1.425491
25	29	0	0.382341	-0.293147	-0.390560
26	6	0	-3.412094	0.900083	0.826591
27	6	0	-3.289573	0.735905	-0.549036
28	6	0	-2.552342	-0.327648	-1.059580
29	6	0	-1.931884	-1.243346	-0.198305
30	6	0	-2.031255	-1.040983	1.187038
31	6	0	-2.773744	0.018098	1.694190
32	1	0	-4.007074	1.714276	1.224064
33	1	0	-3.789042	1.420680	-1.224715
34	1	0	-2.487503	-0.480313	-2.132417
35	1	0	-1.558444	-1.747969	1.861740
36	1	0	-2.869843	0.146035	2.766205
37	6	0	-1.142331	-2.418453	-0.747519
38	1	0	-1.220416	-3.268532	-0.057559
39	1	0	-1.566492	-2.727467	-1.711133
40	8	0	0.196697	-2.028772	-0.911241

CuLH

Center Number	Atomic Number	Atomic Type	Coordinates (Angstroms) X	Y	Z
1	6	0	2.309998	0.278122	0.263363
2	6	0	1.724526	-0.884815	1.049489
3	1	0	3.155319	0.631180	0.865262
4	1	0	2.724242	-0.048431	-0.692109
5	1	0	1.216867	-0.552673	1.953813
6	1	0	2.546556	-1.528607	1.363693
7	7	0	1.429131	1.455026	-0.001545
8	7	0	0.768396	-1.770245	0.294395
9	6	0	1.471966	-2.674626	-0.667955
10	1	0	2.060481	-3.385887	-0.089848
11	1	0	0.730027	-3.204211	-1.261213

12	1	0	2.124388	-2.093328	-1.314085
13	6	0	-0.098603	-2.548932	1.235456
14	1	0	-0.741417	-3.210114	0.657981
15	1	0	0.546130	-3.131113	1.891311
16	1	0	-0.701950	-1.847490	1.809712
17	6	0	2.319151	2.525327	-0.552820
18	1	0	1.735217	3.422385	-0.760765
19	1	0	3.096006	2.774374	0.177600
20	1	0	2.792508	2.176171	-1.472216
21	6	0	0.808319	1.949114	1.257546
22	1	0	1.577405	2.138140	2.014491
23	1	0	0.279593	2.879340	1.054617
24	1	0	0.091796	1.219606	1.635370
25	29	0	0.011165	1.342977	-1.374094
26	1	0	0.142459	-1.170578	-0.242175

CuHL

Center Number	Atomic Number	Atomic Type	Coordinates (Angstroms) X	Y	Z
1	6	0	2.425335	0.226200	0.518121
2	6	0	1.803525	-0.986002	1.177219
3	1	0	3.101107	0.749644	1.199691
4	1	0	2.986073	-0.043016	-0.377066
5	1	0	1.285222	-0.719012	2.098406
6	1	0	2.585181	-1.702292	1.443923
7	7	0	1.374883	1.233213	0.089400
8	7	0	0.813440	-1.598641	0.244182
9	6	0	1.486220	-2.492887	-0.741549
10	1	0	1.899943	-3.365428	-0.230643
11	1	0	0.762178	-2.825163	-1.485312
12	1	0	2.294454	-1.964168	-1.246385
13	6	0	-0.216004	-2.364445	1.000670
14	1	0	-0.907695	-2.829580	0.297711
15	1	0	0.261447	-3.145096	1.598719
16	1	0	-0.765469	-1.692164	1.663023
17	6	0	1.999255	2.241435	-0.828226
18	1	0	1.271446	3.012167	-1.066657
19	1	0	2.846759	2.681817	-0.297407
20	1	0	2.351019	1.756157	-1.735403
21	6	0	0.742938	1.922728	1.257689
22	1	0	1.543065	2.405432	1.824394
23	1	0	0.042758	2.668983	0.892991
24	1	0	0.229261	1.211008	1.899967
25	29	0	0.089275	0.010071	-0.655182
26	1	0	-0.257699	1.307350	-1.194965

CuLH

Center Number	Atomic Number	Atomic Type	Coordinates (Angstroms) X	Y	Z
1	6	0	2.455641	0.245588	0.649304
2	6	0	1.745086	-0.973915	1.221217
3	1	0	3.137415	0.661565	1.407225
4	1	0	3.072775	-0.050874	-0.201923
5	1	0	1.183676	-0.690101	2.114315
6	1	0	2.490994	-1.719595	1.536940
7	7	0	1.499317	1.255216	0.181346
8	7	0	0.796134	-1.547308	0.258486
9	6	0	1.487195	-2.328485	-0.771878
10	1	0	2.034243	-3.176782	-0.330982
11	1	0	0.756370	-2.709565	-1.485947
12	1	0	2.193188	-1.702774	-1.319532
13	6	0	-0.185071	-2.392829	0.943032
14	1	0	-0.891558	-2.790266	0.213669
15	1	0	0.298777	-3.233106	1.465603
16	1	0	-0.742389	-1.797640	1.668330
17	6	0	2.132561	2.168596	-0.771647
18	1	0	1.396035	2.892016	-1.122990
19	1	0	2.975930	2.711304	-0.315844
20	1	0	2.496655	1.607374	-1.633954
21	6	0	0.934949	2.017052	1.298436
22	1	0	1.717216	2.569261	1.843208
23	1	0	0.200017	2.726382	0.916207
24	1	0	0.424373	1.352844	1.997198
25	29	0	-0.181028	0.144629	-0.759157
26	1	0	-1.380584	0.459722	-1.653353

CuL_2H

Center Number	Atomic Number	Atomic Type	Coordinates (Angstroms) X	Y	Z
1	6	0	-2.635589	1.361535	0.028040
2	6	0	-3.032661	-0.037888	0.467878
3	1	0	-3.494710	1.857732	-0.437896
4	1	0	-2.355263	1.964894	0.893450
5	1	0	-3.381143	-0.613442	-0.393344
6	1	0	-3.872627	0.023442	1.171440
7	7	0	-1.484403	1.323227	-0.908625
8	7	0	-1.895393	-0.767555	1.066476
9	6	0	-1.608488	-0.269999	2.417203
10	1	0	-2.464267	-0.418839	3.087611
11	1	0	-0.746676	-0.802108	2.827490

12	1	0	-1.373551	0.795019	2.379031
13	6	0	-2.191088	-2.210228	1.131511
14	1	0	-1.332451	-2.721668	1.569398
15	1	0	-3.076735	-2.409050	1.747370
16	1	0	-2.365108	-2.609011	0.129641
17	6	0	-0.820239	2.640469	-0.931433
18	1	0	0.031482	2.615235	-1.614764
19	1	0	-1.507315	3.427244	-1.268277
20	1	0	-0.483726	2.891558	0.080109
21	6	0	-1.936904	0.994169	-2.280059
22	1	0	-2.589395	1.781125	-2.676450
23	1	0	-1.070842	0.889565	-2.935951
24	1	0	-2.488718	0.053705	-2.280961
25	6	0	2.476211	-1.324627	0.154290
26	6	0	3.138800	0.036286	0.253182
27	1	0	3.246726	-2.034620	-0.169076
28	1	0	2.126455	-1.657810	1.133551
29	1	0	3.498616	0.375919	-0.718249
30	1	0	3.996195	-0.033264	0.923250
31	7	0	1.331697	-1.392405	-0.788234
32	7	0	2.223071	1.099020	0.793243
33	6	0	2.034255	1.003507	2.268410
34	1	0	2.992617	1.166741	2.762299
35	1	0	1.320875	1.767046	2.576089
36	1	0	1.646239	0.015554	2.506121
37	6	0	2.639592	2.470189	0.378970
38	1	0	1.874598	3.182162	0.686858
39	1	0	3.591261	2.700165	0.855821
40	1	0	2.745572	2.506253	-0.703126
41	6	0	0.842472	-2.797151	-0.758691
42	1	0	-0.020567	-2.906055	-1.414091
43	1	0	1.632267	-3.471622	-1.108289
44	1	0	0.556089	-3.075583	0.254427
45	6	0	1.788229	-1.101093	-2.171014
46	1	0	2.643242	-1.734998	-2.433229
47	1	0	0.971523	-1.309996	-2.862443
48	1	0	2.061397	-0.052029	-2.286120
49	29	0	-0.310264	-0.328295	-0.329027
50	1	0	1.293263	0.930257	0.370371

LHCuCl$_2$

Center Number	Atomic Number	Atomic Type	Coordinates (Angstroms) X	Y	Z
1	6	0	-0.162889	1.297374	1.901566
2	6	0	-0.095330	-0.224013	1.877135
3	1	0	0.398514	1.733523	2.728835
4	1	0	-0.526494	-0.588253	2.827484
5	1	0	-0.747807	-0.579921	1.074480

6	1	0	-1.203749	1.602945	2.007740
7	6	0	1.200280	-2.118614	1.172728
8	1	0	2.213787	-2.439382	0.924244
9	1	0	0.792769	-2.800054	1.939637
10	1	0	0.588308	-2.195596	0.271436
11	6	0	2.129690	-0.595351	2.766485
12	1	0	1.814902	-1.215960	3.623287
13	1	0	3.136255	-0.891219	2.464951
14	1	0	2.178480	0.443022	3.103598
15	6	0	1.792049	2.316421	0.743464
16	1	0	2.122551	2.682351	-0.227475
17	1	0	1.869680	3.105645	1.491767
18	1	0	2.385274	1.448039	1.020357
19	6	0	-0.481439	3.017433	0.144669
20	1	0	-0.062387	3.394219	-0.786672
21	1	0	-1.487239	2.641438	-0.034988
22	1	0	-0.499584	3.805823	0.897116
23	7	0	1.235886	-0.736235	1.628786
24	7	0	0.367044	1.901079	0.631098
25	29	0	2.406746	-0.324002	-1.273321
26	17	0	4.397171	-0.572095	-0.513923
27	17	0	0.365299	0.080198	-1.907772
28	1	0	0.339639	1.169294	-0.113947

CuL

Center Number	Atomic Number	Atomic Type	Coordinates (Angstroms) X	Y	Z
1	6	0	2.418809	0.246197	0.609737
2	6	0	1.713974	-0.968365	1.201651
3	1	0	3.127106	0.645736	1.353451
4	1	0	3.011497	-0.057574	-0.256204
5	1	0	1.141879	-0.669084	2.082880
6	1	0	2.473137	-1.688708	1.547050
7	7	0	1.480250	1.282641	0.168503
8	7	0	0.785175	-1.596536	0.257097
9	6	0	1.492676	-2.376818	-0.757824
10	1	0	2.070575	-3.198301	-0.304148
11	1	0	0.769451	-2.796386	-1.458540
12	1	0	2.172752	-1.740991	-1.326480
13	6	0	-0.173172	-2.446234	0.962345
14	1	0	-0.870645	-2.879273	0.243959
15	1	0	0.328716	-3.262321	1.506927
16	1	0	-0.745862	-1.845823	1.671738
17	6	0	2.126583	2.199583	-0.769281
18	1	0	1.403156	2.943955	-1.104880
19	1	0	2.982574	2.718293	-0.308153
20	1	0	2.475467	1.647057	-1.643751

21	6	0	0.930489	2.029967	1.299381
22	1	0	1.722318	2.552796	1.859955
23	1	0	0.212407	2.764038	0.931144
24	1	0	0.400413	1.362214	1.980014
25	29	0	-0.235615	0.142153	-0.801322

3a'-H-tBuO

Center	Atomic	Atomic	Coordinates (Angstroms)		
Number	Number	Type	X	Y	Z
1	6	0	0.249232	-1.536877	1.954632
2	6	0	-0.612571	-0.389298	2.206357
3	1	0	-0.224202	-2.468893	2.278182
4	6	0	-0.082432	0.761068	2.873274
5	7	0	0.333293	1.673441	3.441486
6	6	0	-1.887455	-0.319594	1.643886
7	6	0	-2.365179	-1.434993	0.884658
8	6	0	-2.643029	0.896287	1.641922
9	6	0	-3.578019	-1.319162	0.187654
10	1	0	-1.949233	-2.417943	1.039009
11	6	0	-3.794300	0.990149	0.923413
12	1	0	-2.270670	1.750055	2.196389
13	6	0	-4.262568	-0.131316	0.190950
14	1	0	-3.963371	-2.173231	-0.353272
15	1	0	-4.357009	1.915104	0.907874
16	1	0	-5.195733	-0.049599	-0.355730
17	6	0	1.692820	-1.455826	2.333551
18	6	0	2.544685	-0.606017	1.625653
19	6	0	2.218575	-2.236369	3.361845
20	6	0	3.892391	-0.525985	1.951838
21	1	0	2.145984	-0.011421	0.809549
22	6	0	3.567896	-2.160373	3.686957
23	1	0	1.566716	-2.906289	3.913731
24	6	0	4.407150	-1.302850	2.984140
25	1	0	4.542544	0.139684	1.395531
26	1	0	3.963826	-2.770579	4.490998
27	1	0	5.459463	-1.242834	3.237817
28	1	0	0.088961	-1.564040	0.787950
29	6	0	-0.326679	-2.305152	-1.511960
30	6	0	0.641927	-3.332258	-0.900928
31	1	0	0.179719	-3.836006	-0.045071
32	1	0	0.922159	-4.099504	-1.628952
33	1	0	1.554770	-2.837654	-0.554684
34	6	0	-1.595373	-3.025852	-1.992662
35	1	0	-2.074540	-3.557982	-1.164234
36	1	0	-2.307440	-2.300425	-2.395119
37	1	0	-1.366100	-3.759851	-2.771549
38	6	0	0.352728	-1.622242	-2.712250

39	1	0	-0.323734	-0.885808	-3.152963
40	1	0	1.257596	-1.105104	-2.382675
41	1	0	0.626090	-2.354256	-3.479689
42	8	0	-0.636827	-1.285460	-0.607463

3a'…CuLH$^+$

Center Number	Atomic Number	Atomic Type	Coordinates (Angstroms) X	Y	Z
1	6	0	-0.319382	1.234637	-0.655063
2	6	0	-1.233899	0.843803	0.414033
3	1	0	-0.826658	1.627645	-1.533738
4	6	0	-0.703685	0.245082	1.577624
5	7	0	-0.238655	-0.305148	2.491318
6	6	0	-2.694929	0.905754	0.300425
7	6	0	-3.314875	1.722072	-0.658560
8	6	0	-3.516349	0.139543	1.145624
9	6	0	-4.697271	1.758565	-0.772743
10	1	0	-2.718140	2.357987	-1.300934
11	6	0	-4.896297	0.184014	1.030479
12	1	0	-3.066141	-0.494299	1.902667
13	6	0	-5.496380	0.989572	0.066424
14	1	0	-5.153542	2.404118	-1.514550
15	1	0	-5.508244	-0.413441	1.696636
16	1	0	-6.575714	1.025270	-0.022302
17	6	0	0.983994	1.913026	-0.426233
18	6	0	1.788782	2.215159	-1.539462
19	6	0	1.441660	2.324744	0.833714
20	6	0	2.982445	2.903754	-1.403876
21	1	0	1.448753	1.916760	-2.526731
22	6	0	2.650149	3.005962	0.969207
23	1	0	0.836428	2.158822	1.716256
24	6	0	3.427616	3.300479	-0.142926
25	1	0	3.568882	3.138919	-2.285122
26	1	0	2.968049	3.329577	1.954423
27	1	0	4.358166	3.845092	-0.035015
28	6	0	1.044956	-2.965469	0.437205
29	6	0	2.426045	-2.390375	0.151494
30	1	0	1.144800	-4.054115	0.524640
31	1	0	0.672332	-2.598597	1.395971
32	1	0	2.644535	-2.384858	-0.916202
33	1	0	3.190328	-2.993516	0.642850
34	7	0	0.011082	-2.666251	-0.587717
35	7	0	2.624826	-0.982570	0.642878
36	6	0	2.851019	-0.908819	2.115723
37	1	0	3.794215	-1.404030	2.341231
38	1	0	2.891028	0.141829	2.396482
39	1	0	2.022268	-1.380609	2.636062

40	6	0	3.729480	-0.315180	-0.103199
41	1	0	3.864742	0.686821	0.298421
42	1	0	4.636695	-0.905368	0.020997
43	1	0	3.451035	-0.252806	-1.153178
44	6	0	-1.300923	-3.088173	-0.030586
45	1	0	-2.084929	-2.896418	-0.762267
46	1	0	-1.288729	-4.158639	0.206898
47	1	0	-1.513716	-2.520387	0.876101
48	6	0	0.267251	-3.430285	-1.829313
49	1	0	0.300010	-4.505843	-1.618068
50	1	0	-0.532180	-3.231970	-2.542876
51	1	0	1.208843	-3.124427	-2.283851
52	29	0	-0.165585	-0.714458	-0.940053
53	1	0	1.771643	-0.443645	0.441438

10 (3a'-H+)

Center Number	Atomic Number	Atomic Type	Coordinates (Angstroms) X	Y	Z
1	6	0	0.919784	0.512555	-0.898712
2	6	0	-0.291886	0.551615	-1.753703
3	1	0	1.363355	1.500579	-0.797737
4	6	0	-0.821035	-0.700364	-2.285695
5	7	0	-1.258929	-1.663481	-2.732599
6	6	0	-1.270311	1.266432	-0.798334
7	6	0	-1.475518	2.639003	-0.932844
8	6	0	-1.904945	0.549543	0.214969
9	6	0	-2.344729	3.286923	-0.067059
10	1	0	-0.977104	3.195159	-1.719496
11	6	0	-2.777277	1.204068	1.072356
12	1	0	-1.739691	-0.517029	0.325797
13	6	0	-2.995010	2.570712	0.933084
14	1	0	-2.520472	4.349824	-0.178519
15	1	0	-3.286510	0.646691	1.848757
16	1	0	-3.675600	3.079444	1.605167
17	6	0	1.506418	-0.494839	-0.148683
18	6	0	2.592320	-0.105650	0.695609
19	6	0	1.104703	-1.864687	-0.172912
20	6	0	3.235598	-1.034236	1.476552
21	1	0	2.897874	0.934207	0.715733
22	6	0	1.759663	-2.779503	0.612851
23	1	0	0.297881	-2.191766	-0.813027
24	6	0	2.817328	-2.366741	1.433333
25	1	0	4.056107	-0.740763	2.117860
26	1	0	1.463327	-3.820094	0.597163
27	1	0	3.325668	-3.101079	2.048042
28	1	0	-0.096384	1.213211	-2.602264

3a'-CuLH

Center Number	Atomic Number	Atomic Type	Coordinates (Angstroms) X	Y	Z
1	6	0	-0.144643	-1.082148	-1.471386
2	6	0	1.254925	-1.337820	-1.121971
3	6	0	2.131244	-1.481574	-2.228830
4	7	0	2.831554	-1.573879	-3.146849
5	6	0	1.874921	-1.312131	0.204740
6	6	0	1.147999	-0.983301	1.366188
7	6	0	3.257498	-1.533144	0.351417
8	6	0	1.772280	-0.867441	2.598948
9	1	0	0.077475	-0.845040	1.305138
10	6	0	3.876354	-1.415660	1.585427
11	1	0	3.852151	-1.793898	-0.516715
12	6	0	3.142431	-1.076135	2.718964
13	1	0	1.184618	-0.624010	3.477763
14	1	0	4.943084	-1.591798	1.662714
15	1	0	3.629021	-0.989230	3.683013
16	6	0	-1.322216	-1.677037	-0.774893
17	6	0	-2.608122	-1.255045	-1.148638
18	6	0	-1.227969	-2.686667	0.189273
19	6	0	-3.745867	-1.789734	-0.560949
20	1	0	-2.708129	-0.507579	-1.932127
21	6	0	-2.366985	-3.206404	0.795948
22	1	0	-0.257153	-3.084303	0.458616
23	6	0	-3.631571	-2.756595	0.435111
24	1	0	-4.726998	-1.460165	-0.887576
25	1	0	-2.261996	-3.985565	1.542587
26	1	0	-4.516991	-3.173223	0.900402
27	1	0	-0.289986	-1.114680	-2.552133
28	6	0	0.059104	2.803448	1.010720
29	6	0	-1.453948	2.891311	0.869595
30	1	0	0.387396	3.769227	1.417461
31	1	0	0.338953	2.043969	1.743843
32	1	0	-1.738390	3.533858	0.037110
33	1	0	-1.870025	3.330138	1.777478
34	7	0	0.817746	2.523318	-0.233322
35	7	0	-2.167460	1.582793	0.666502
36	6	0	-2.231297	0.743704	1.897630
37	1	0	-2.909013	1.220125	2.604641
38	1	0	-2.598918	-0.244392	1.619654
39	1	0	-1.238383	0.661149	2.332531
40	6	0	-3.531574	1.815603	0.111274
41	1	0	-4.044056	0.859478	0.044459
42	1	0	-4.068041	2.487786	0.780861
43	1	0	-3.437393	2.260420	-0.878273
44	6	0	2.249408	2.370244	0.143736
45	1	0	2.838830	2.168287	-0.750271

46	1	0	2.618588	3.288844	0.614978
47	1	0	2.362380	1.535522	0.837423
48	6	0	0.696982	3.641348	-1.196480
49	1	0	1.002498	4.586379	-0.731713
50	1	0	1.343101	3.445309	-2.051518
51	1	0	-0.325287	3.731515	-1.562322
52	29	0	0.292467	0.789641	-1.055139
53	1	0	-1.635484	1.037734	-0.038259

1a

Center Number	Atomic Number	Atomic Type	Coordinates (Angstroms) X	Y	Z
1	6	0	1.576746	0.013303	0.917679
2	6	0	0.132025	0.045797	0.448201
3	6	0	-0.560452	1.230534	0.223085
4	6	0	-0.515953	-1.172108	0.240899
5	6	0	-1.884772	1.198313	-0.204746
6	1	0	-0.071265	2.185864	0.379840
7	6	0	-1.836373	-1.203905	-0.185648
8	1	0	0.016587	-2.102171	0.414484
9	6	0	-2.525757	-0.015950	-0.410190
10	1	0	-2.413371	2.128914	-0.376932
11	1	0	-2.327670	-2.157358	-0.343143
12	1	0	-3.556853	-0.038964	-0.743655
13	1	0	1.649516	-0.516707	1.872092
14	1	0	2.191327	-0.541277	0.202382
15	6	0	2.186354	1.326461	1.096359
16	7	0	2.671217	2.359380	1.239068

3a

Center Number	Atomic Number	Atomic Type	Coordinates (Angstroms) X	Y	Z
1	6	0	-0.403059	0.287331	-0.757256
2	6	0	0.432700	-0.096033	0.494648
3	1	0	-0.060135	1.276593	-1.070472
4	1	0	-0.160369	-0.408837	-1.564309
5	1	0	0.147930	0.573804	1.312166
6	6	0	0.099365	-1.453318	0.937289
7	7	0	-0.165141	-2.524778	1.261828
8	6	0	1.920844	0.056301	0.227712
9	6	0	2.554373	1.249407	0.566977
10	6	0	2.648801	-0.943756	-0.412912
11	6	0	3.898656	1.441776	0.270400
12	1	0	1.995238	2.032981	1.068962

13	6	0	3.992980	-0.752607	-0.708733
14	1	0	2.168195	-1.881595	-0.672719
15	6	0	4.620797	0.440681	-0.368893
16	1	0	4.382297	2.372660	0.543789
17	1	0	4.551269	-1.539801	-1.202388
18	1	0	5.670181	0.587771	-0.597255
19	6	0	-1.886761	0.307761	-0.493844
20	6	0	-2.485420	1.437655	0.064702
21	6	0	-2.679124	-0.806338	-0.766058
22	6	0	-3.846002	1.455187	0.345646
23	1	0	-1.880694	2.315013	0.275872
24	6	0	-4.040886	-0.792697	-0.485917
25	1	0	-2.225960	-1.693553	-1.196341
26	6	0	-4.627535	0.337708	0.070929
27	1	0	-4.297248	2.342296	0.775892
28	1	0	-4.643541	-1.667470	-0.702570
29	1	0	-5.689546	0.349238	0.288430

3a'

Center Number	Atomic Number	Atomic Type	Coordinates (Angstroms) X	Y	Z
1	6	0	0.473171	0.573375	0.013729
2	6	0	-0.498303	-0.359114	-0.030247
3	1	0	0.132456	1.600503	0.114308
4	6	0	-0.200044	-1.761908	-0.019952
5	7	0	-0.013759	-2.899322	0.004747
6	6	0	-1.944028	-0.009182	-0.032900
7	6	0	-2.395031	1.138712	-0.689581
8	6	0	-2.872044	-0.818342	0.627149
9	6	0	-3.739369	1.485178	-0.659439
10	1	0	-1.696347	1.751530	-1.248178
11	6	0	-4.216059	-0.471054	0.653747
12	1	0	-2.540913	-1.721417	1.128544
13	6	0	-4.653821	0.683709	0.014876
14	1	0	-4.074898	2.376111	-1.177893
15	1	0	-4.922813	-1.105793	1.175760
16	1	0	-5.703943	0.951707	0.032649
17	6	0	1.929993	0.415355	-0.002318
18	6	0	2.697996	1.421032	0.599377
19	6	0	2.590659	-0.662385	-0.603739
20	6	0	4.081869	1.335601	0.634694
21	1	0	2.199415	2.272451	1.051298
22	6	0	3.975979	-0.741005	-0.575759
23	1	0	2.028858	-1.435077	-1.112111
24	6	0	4.725097	0.250709	0.048436
25	1	0	4.658671	2.117859	1.114439
26	1	0	4.472636	-1.579842	-1.049459
27	1	0	5.806816	0.183131	0.068972

$CuLCl_2$

Center Number	Atomic Number	Atomic Type	Coordinates (Angstroms) X	Y	Z
1	6	0	0.216865	1.638434	0.551490
2	6	0	-0.906112	1.709293	-0.463896
3	1	0	0.831116	2.545756	0.516501
4	1	0	-0.191415	1.562721	1.561105
5	1	0	-0.498718	1.799190	-1.472720
6	1	0	-1.533822	2.590530	-0.286802
7	7	0	1.040529	0.439504	0.294800
8	7	0	-1.711388	0.472341	-0.401312
9	6	0	-2.667856	0.539390	0.720740
10	1	0	-3.341405	1.395370	0.587942
11	1	0	-3.243722	-0.382332	0.755182
12	1	0	-2.137995	0.643469	1.667642
13	6	0	-2.454880	0.271111	-1.658734
14	1	0	-3.023331	-0.655363	-1.587632
15	1	0	-3.138102	1.109896	-1.841762
16	1	0	-1.755125	0.190703	-2.492352
17	6	0	1.772249	0.036847	1.510656
18	1	0	2.352492	-0.857419	1.288212
19	1	0	2.441101	0.840438	1.842734
20	1	0	1.063304	-0.192022	2.308135
21	6	0	2.008504	0.703391	-0.788262
22	1	0	2.678060	1.523210	-0.500314
23	1	0	2.583646	-0.199724	-0.977529
24	1	0	1.487061	0.971783	-1.707028
25	29	0	-0.319549	-1.107877	-0.184814
26	17	0	1.301487	-2.514077	-0.827260
27	17	0	-1.900084	-2.635806	0.235011

$Cu^{II}L_2H$

Center Number	Atomic Number	Atomic Type	Coordinates (Angstroms) X	Y	Z
1	6	0	-3.022143	0.961201	-0.257341
2	6	0	-2.131145	2.000301	0.396544
3	1	0	-4.054824	1.109881	0.081950
4	1	0	-3.029353	1.097448	-1.340122
5	1	0	-2.158364	1.901176	1.484043
6	1	0	-2.495347	3.006114	0.153819
7	7	0	-2.561726	-0.410689	0.037605
8	7	0	-0.710028	1.867471	-0.026584
9	6	0	-0.555396	2.291498	-1.436561
10	1	0	-0.936068	3.309058	-1.583152

11	1	0	0.501482	2.274852	-1.705524
12	1	0	-1.088523	1.612389	-2.102180
13	6	0	0.098623	2.755902	0.832744
14	1	0	1.128455	2.790073	0.473132
15	1	0	-0.289517	3.780257	0.808354
16	1	0	0.085133	2.393310	1.860925
17	6	0	-3.060230	-1.331164	-0.996841
18	1	0	-2.741475	-2.349462	-0.769999
19	1	0	-4.156353	-1.323362	-1.048550
20	1	0	-2.658606	-1.044904	-1.971308
21	6	0	-3.065488	-0.836767	1.355874
22	1	0	-4.162900	-0.826498	1.378274
23	1	0	-2.728227	-1.849114	1.576941
24	1	0	-2.695405	-0.174532	2.139408
25	29	0	-0.355734	-0.143775	0.057977
26	6	0	3.050979	-0.876479	-0.341047
27	6	0	2.148036	-1.858699	0.380238
28	1	0	4.092579	-1.096786	-0.104518
29	1	0	2.939569	-0.935862	-1.423792
30	1	0	2.188881	-1.703413	1.459425
31	1	0	2.563218	-2.856779	0.195812
32	7	0	2.802840	0.554386	0.041223
33	7	0	0.721311	-1.854601	-0.026369
34	6	0	0.598386	-2.337935	-1.424635
35	1	0	1.134483	-3.285632	-1.553364
36	1	0	-0.452561	-2.497603	-1.660296
37	1	0	0.993252	-1.599450	-2.123580
38	6	0	0.032307	-2.821048	0.867170
39	1	0	-1.008772	-2.914827	0.567002
40	1	0	0.505179	-3.807357	0.804780
41	1	0	0.078990	-2.466932	1.897677
42	6	0	3.388831	1.483196	-0.967628
43	1	0	3.176213	2.509374	-0.675222
44	1	0	4.466458	1.327755	-1.003651
45	1	0	2.953722	1.263046	-1.940466
46	6	0	3.265949	0.881406	1.422358
47	1	0	4.342250	0.724950	1.477965
48	1	0	3.032353	1.923387	1.631187
49	1	0	2.755766	0.244581	2.140341
50	1	0	1.775589	0.669891	0.026979

1a-CuL

Center Number	Atomic Number	Atomic Type	Coordinates (Angstroms) X	Y	Z
1	6	0	-3.509742	1.569208	0.165331
2	6	0	-4.069180	0.337279	-0.514023

3	1	0	-3.943220	2.480585	-0.253377
4	1	0	-3.726572	1.566826	1.233687
5	1	0	-3.959719	0.405617	-1.598100
6	1	0	-5.133027	0.212100	-0.297135
7	7	0	-2.026432	1.595488	-0.002984
8	7	0	-3.307863	-0.860564	-0.057407
9	6	0	-3.716099	-1.297772	1.304654
10	1	0	-4.758703	-1.627417	1.286378
11	1	0	-3.089442	-2.133372	1.620395
12	1	0	-3.612029	-0.483725	2.020344
13	6	0	-3.473360	-1.987405	-1.008018
14	1	0	-2.934153	-2.859861	-0.637342
15	1	0	-4.534201	-2.238484	-1.092068
16	1	0	-3.095164	-1.709138	-1.991654
17	6	0	-1.378813	2.353234	1.094217
18	1	0	-0.299998	2.381240	0.938869
19	1	0	-1.760474	3.378833	1.101379
20	1	0	-1.605171	1.880040	2.050406
21	6	0	-1.631551	2.144042	-1.325307
22	1	0	-1.919161	3.197054	-1.383642
23	1	0	-0.551602	2.062312	-1.445541
24	1	0	-2.127047	1.593939	-2.124607
25	29	0	-1.422968	-0.302279	0.012386
26	6	0	2.789068	-1.920840	0.322676
27	6	0	3.836714	-0.833449	0.131907
28	6	0	4.353746	-0.591602	-1.137840
29	6	0	4.275941	-0.087183	1.222013
30	6	0	5.313650	0.397009	-1.315719
31	1	0	4.020271	-1.184054	-1.984007
32	6	0	5.235145	0.901272	1.039153
33	1	0	3.884577	-0.283733	2.215051
34	6	0	5.752893	1.144101	-0.228349
35	1	0	5.725592	0.576807	-2.301349
36	1	0	5.586792	1.473458	1.889328
37	1	0	6.507968	1.908552	-0.367324
38	1	0	2.887953	-2.710911	-0.427237
39	1	0	2.855836	-2.390545	1.308206
40	6	0	1.450943	-1.371363	0.196786
41	7	0	0.401531	-0.916243	0.088286

CuLH-PhCHCN

Center Number	Atomic Number	Atomic Type	Coordinates (Angstroms) X	Y	Z
1	6	0	-2.536202	1.919837	0.379811
2	6	0	-3.674019	1.121229	-0.238681
3	1	0	-2.605938	2.972123	0.074391
4	1	0	-2.615907	1.894141	1.469082

5	1	0	-3.621309	1.177002	-1.328299
6	1	0	-4.641642	1.546416	0.060204
7	7	0	-1.234175	1.342643	0.001524
8	7	0	-3.579787	-0.299871	0.146191
9	6	0	-4.082183	-0.517569	1.510921
10	1	0	-5.144450	-0.247621	1.579688
11	1	0	-3.951884	-1.566545	1.772384
12	1	0	-3.515850	0.078232	2.226333
13	6	0	-4.329668	-1.148779	-0.790401
14	1	0	-4.206980	-2.192605	-0.500937
15	1	0	-5.398385	-0.894519	-0.784753
16	1	0	-3.938023	-1.020602	-1.800844
17	6	0	-0.188964	1.715992	0.965227
18	1	0	0.750446	1.242463	0.678791
19	1	0	-0.045312	2.805030	0.992515
20	1	0	-0.457125	1.369569	1.964116
21	6	0	-0.830297	1.781339	-1.341775
22	1	0	-0.664965	2.867278	-1.364571
23	1	0	0.085435	1.264571	-1.625681
24	1	0	-1.597017	1.524208	-2.073616
25	29	0	-1.520819	-0.807629	0.053515
26	6	0	2.484689	-1.814966	-0.211678
27	6	0	3.526898	-0.815572	-0.071781
28	6	0	3.357564	0.522477	-0.474701
29	6	0	4.774860	-1.164159	0.477044
30	6	0	4.370077	1.457989	-0.318313
31	1	0	2.422567	0.823240	-0.938029
32	6	0	5.786570	-0.227021	0.626341
33	1	0	4.942140	-2.189584	0.792256
34	6	0	5.595178	1.095905	0.235386
35	1	0	4.204636	2.480575	-0.643068
36	1	0	6.735696	-0.532822	1.054869
37	1	0	6.386450	1.827336	0.352987
38	1	0	2.711085	-2.854955	-0.009742
39	6	0	1.221700	-1.509387	-0.568589
40	7	0	0.119022	-1.223525	-0.905494
41	1	0	-1.722563	-2.190315	0.731566

$CuLHCl_2$

Center Number	Atomic Number	Atomic Type	Coordinates (Angstroms) X	Y	Z
1	6	0	-0.162889	1.297374	1.901566
2	6	0	-0.095330	-0.224013	1.877135
3	1	0	0.398514	1.733523	2.728835
4	1	0	-0.526494	-0.588253	2.827484
5	1	0	-0.747807	-0.579921	1.074480
6	1	0	-1.203749	1.602945	2.007740

7	6	0	1.200280	-2.118614	1.172728
8	1	0	2.213787	-2.439382	0.924244
9	1	0	0.792769	-2.800054	1.939637
10	1	0	0.588308	-2.195596	0.271436
11	6	0	2.129690	-0.595351	2.766485
12	1	0	1.814902	-1.215960	3.623287
13	1	0	3.136255	-0.891219	2.464951
14	1	0	2.178480	0.443022	3.103598
15	6	0	1.792049	2.316421	0.743464
16	1	0	2.122551	2.682351	-0.227475
17	1	0	1.869680	3.105645	1.491767
18	1	0	2.385274	1.448039	1.020357
19	6	0	-0.481439	3.017433	0.144669
20	1	0	-0.062387	3.394219	-0.786672
21	1	0	-1.487239	2.641438	-0.034988
22	1	0	-0.499584	3.805823	0.897116
23	7	0	1.235886	-0.736235	1.628786
24	7	0	0.367044	1.901079	0.631098
25	29	0	2.406746	-0.324002	-1.273321
26	17	0	4.397171	-0.572095	-0.513923
27	17	0	0.365299	0.080198	-1.907772
28	1	0	0.339639	1.169294	-0.113947

14

Center Number	Atomic Number	Atomic Type	Coordinates (Angstroms) X	Y	Z
1	6	0	0.928441	0.720311	1.410340
2	6	0	-0.500539	0.377937	1.194194
3	1	0	1.175813	1.726794	1.083110
4	6	0	-0.948187	-0.775540	1.916482
5	7	0	-1.286778	-1.681167	2.541370
6	6	0	-1.393966	1.017282	0.329841
7	6	0	-1.026677	2.217739	-0.349027
8	6	0	-2.699198	0.476206	0.121838
9	6	0	-1.921515	2.834060	-1.186278
10	1	0	-0.049384	2.656357	-0.202288
11	6	0	-3.582190	1.105612	-0.720269
12	1	0	-2.990451	-0.436249	0.629051
13	6	0	-3.193952	2.279529	-1.372320
14	1	0	-1.649331	3.747589	-1.698189
15	1	0	-4.570843	0.696403	-0.880290
16	1	0	-3.894533	2.773976	-2.035675
17	6	0	1.663291	-0.345431	0.593187
18	6	0	2.274011	-1.422626	1.235521
19	6	0	1.698608	-0.251969	-0.798504
20	6	0	2.951349	-2.375949	0.489208
21	1	0	2.233855	-1.507252	2.315886

22	6	0	2.382644	-1.204853	-1.540588
23	1	0	1.204303	0.568656	-1.308517
24	6	0	3.007086	-2.267666	-0.897324
25	1	0	3.439703	-3.202575	0.990767
26	1	0	2.423495	-1.119924	-2.619658
27	1	0	3.536163	-3.014757	-1.476886
28	1	0	1.182865	0.621393	2.466434

1a-CuLH

Center Number	Atomic Number	Atomic Type	Coordinates (Angstroms) X	Y	Z
1	6	0	-1.078746	0.434074	-0.439187
2	6	0	0.186937	1.006355	0.154702
3	1	0	-1.935687	1.071395	-0.205483
4	1	0	-1.015327	0.364253	-1.526338
5	1	0	0.086779	1.155652	1.231135
6	1	0	0.444615	1.969006	-0.293775
7	7	0	-1.284598	-0.939720	0.104524
8	7	0	1.335694	0.055043	-0.053904
9	6	0	1.815532	0.104459	-1.467051
10	1	0	2.128518	1.127100	-1.690306
11	1	0	2.663084	-0.566438	-1.586661
12	1	0	1.018760	-0.186829	-2.147740
13	6	0	2.448490	0.448311	0.859486
14	1	0	3.326362	-0.158578	0.651995
15	1	0	2.680633	1.501682	0.686933
16	1	0	2.136090	0.309615	1.891674
17	6	0	-2.107208	-1.745171	-0.833578
18	1	0	-2.340518	-2.708678	-0.382525
19	1	0	-3.042309	-1.223889	-1.053073
20	1	0	-1.552841	-1.903852	-1.761186
21	6	0	-1.943804	-0.888163	1.435984
22	1	0	-2.934146	-0.435241	1.340721
23	1	0	-2.056838	-1.897340	1.829635
24	1	0	-1.350522	-0.300216	2.137812
25	29	0	0.572674	-1.664387	0.282264
26	6	0	-1.072595	-5.719991	0.871932
27	6	0	-2.509780	-5.409485	0.470694
28	6	0	-3.410531	-4.940199	1.423845
29	6	0	-2.900566	-5.532754	-0.860457
30	6	0	-4.701588	-4.593807	1.043217
31	1	0	-3.112871	-4.859229	2.464390
32	6	0	-4.192663	-5.183645	-1.236797
33	1	0	-2.204903	-5.913743	-1.600686
34	6	0	-5.091429	-4.711314	-0.286568
35	1	0	-5.406097	-4.242133	1.787361

36	1	0	-4.498123	-5.288222	-2.270847
37	1	0	-6.099997	-4.446289	-0.580401
38	1	0	-0.988410	-6.053776	1.909576
39	1	0	-0.606896	-6.478547	0.238875
40	6	0	-0.325240	-4.481142	0.735776
41	7	0	0.126075	-3.436004	0.601117
42	1	0	1.943528	-2.093717	0.366403

CuLH--PhCHCN

Center Number	Atomic Number	Atomic Type	Coordinates (Angstroms) X	Y	Z
1	6	0	-0.680236	1.259576	0.071079
2	6	0	0.785255	1.291621	0.432234
3	1	0	-1.215817	2.071550	0.568918
4	1	0	-0.820580	1.385934	-1.002545
5	1	0	0.928065	1.262147	1.513365
6	1	0	1.272480	2.194168	0.055735
7	7	0	-1.245177	-0.061320	0.467755
8	7	0	1.489019	0.092926	-0.142156
9	6	0	1.687954	0.245181	-1.614860
10	1	0	2.233631	1.172528	-1.801398
11	1	0	2.262102	-0.599593	-1.989886
12	1	0	0.731236	0.284176	-2.134616
13	6	0	2.816850	-0.034970	0.530768
14	1	0	3.389940	-0.835868	0.069815
15	1	0	3.355943	0.910104	0.424192
16	1	0	2.670523	-0.249002	1.588719
17	6	0	-2.454548	-0.364724	-0.335401
18	1	0	-2.888680	-1.304044	0.003281
19	1	0	-3.193839	0.431376	-0.210106
20	1	0	-2.190853	-0.447273	-1.390962
21	6	0	-1.595504	-0.075973	1.911992
22	1	0	-2.409621	0.629337	2.100808
23	1	0	-1.913463	-1.078313	2.196709
24	1	0	-0.735533	0.207607	2.519499
25	29	0	0.261788	-1.349039	0.164506
26	6	0	-1.428381	-5.363570	0.493818
27	6	0	-2.722620	-5.763868	0.097413
28	6	0	-3.679709	-4.849080	-0.416239
29	6	0	-3.083296	-7.131649	0.221970
30	6	0	-4.935338	-5.283506	-0.771735
31	1	0	-3.417534	-3.803768	-0.535875
32	6	0	-4.344090	-7.552614	-0.139469
33	1	0	-2.360447	-7.840448	0.609384
34	6	0	-5.274093	-6.634900	-0.634641
35	1	0	-5.661056	-4.581492	-1.162856
36	1	0	-4.614502	-8.596121	-0.038753

37	1	0	-6.264242	-6.971722	-0.916589
38	1	0	-0.717600	-6.080643	0.890189
39	6	0	-0.980645	-4.056883	0.440047
40	7	0	-0.601782	-2.959277	0.438801
41	1	0	1.398424	-2.191717	-0.079717

$PhCHOH^+$ (11)

Center Number	Atomic Number	Atomic Type	Coordinates (Angstroms) X	Y	Z
1	6	0	1.917478	0.489016	0.000278
2	8	0	2.791078	-0.449205	-0.000190
3	1	0	2.270192	1.520103	0.000266
4	6	0	0.545333	0.209936	0.000137
5	6	0	-0.347235	1.306391	0.000010
6	6	0	0.055261	-1.118000	0.000106
7	6	0	-1.706871	1.075652	-0.000082
8	1	0	0.037585	2.320125	0.000009
9	6	0	-1.303506	-1.329949	-0.000001
10	1	0	0.748449	-1.949729	0.000188
11	6	0	-2.177900	-0.236781	-0.000091
12	1	0	-2.402435	1.904526	-0.000160
13	1	0	-1.698398	-2.337589	-0.000012
14	1	0	-3.247020	-0.415931	-0.000181
15	1	0	3.702268	-0.125332	-0.000270

$PhCHCN^-$ (11)

Center Number	Atomic Number	Atomic Type	Coordinates (Angstroms) X	Y	Z
1	6	0	1.608578	0.474923	0.401037
2	6	0	0.214663	0.280051	0.178710
3	6	0	-0.710207	1.347035	0.335067
4	6	0	-0.344470	-0.967397	-0.207376
5	6	0	-2.068084	1.178266	0.124182
6	1	0	-0.332433	2.322664	0.629558
7	6	0	-1.705029	-1.123239	-0.415231
8	1	0	0.318491	-1.816822	-0.341243
9	6	0	-2.593590	-0.058811	-0.254859
10	1	0	-2.732891	2.027926	0.257480
11	1	0	-2.082941	-2.098898	-0.709704
12	1	0	-3.657623	-0.188245	-0.419284
13	1	0	1.974757	1.451712	0.693837
14	6	0	2.540608	-0.542333	0.258321
15	7	0	3.321527	-1.407206	0.135580

13

Center Number	Atomic Number	Atomic Type	Coordinates (Angstroms) X	Y	Z
1	6	0	-0.440833	0.494583	-0.986496
2	6	0	0.494170	-0.433395	-0.290635
3	1	0	-0.130546	1.527425	-0.765379
4	6	0	-0.000710	-1.606375	0.276295
5	7	0	-0.392013	-2.591582	0.769395
6	6	0	1.900606	-0.146018	-0.207019
7	6	0	2.483476	0.972376	-0.855618
8	6	0	2.794596	-0.957339	0.539716
9	6	0	3.832804	1.271041	-0.730195
10	1	0	1.869648	1.597672	-1.493765
11	6	0	4.140590	-0.654326	0.649939
12	1	0	2.406879	-1.838551	1.040572
13	6	0	4.685093	0.468840	0.024650
14	1	0	4.228405	2.140969	-1.247791
15	1	0	4.780135	-1.308766	1.236067
16	1	0	5.740613	0.701458	0.112877
17	6	0	-1.876802	0.360035	-0.511386
18	6	0	-2.294814	1.058664	0.621013
19	6	0	-2.788341	-0.468505	-1.160532
20	6	0	-3.595525	0.939910	1.092269
21	1	0	-1.583594	1.692648	1.142295
22	6	0	-4.092997	-0.590354	-0.691647
23	1	0	-2.464082	-1.026712	-2.030866
24	6	0	-4.503194	0.114868	0.433308
25	1	0	-3.901919	1.488893	1.976572
26	1	0	-4.789093	-1.246152	-1.204184
27	1	0	-5.519526	0.018213	0.799959
28	8	0	-0.356584	0.331302	-2.426610
29	1	0	-0.888614	1.024770	-2.826945

5…tBuOH (dimer)

Center Number	Atomic Number	Atomic Type	Coordinates (Angstroms) X	Y	Z
1	6	0	-0.653210	-0.380436	0.705630
2	8	0	0.030532	-1.317753	1.061459
3	1	0	-0.204669	0.627637	0.619671
4	6	0	-2.081172	-0.470847	0.360392
5	6	0	-2.754727	0.683831	-0.038856
6	6	0	-2.758559	-1.691425	0.428422

7	6	0	-4.102082	0.622525	-0.370141
8	1	0	-2.220965	1.627995	-0.088036
9	6	0	-4.102316	-1.749992	0.098044
10	1	0	-2.218830	-2.577736	0.741425
11	6	0	-4.772442	-0.593794	-0.300951
12	1	0	-4.627944	1.517624	-0.680319
13	1	0	-4.633640	-2.693089	0.149337
14	1	0	-5.824418	-0.643973	-0.558644
15	1	0	1.790077	-0.626842	1.438577
16	6	0	3.409022	-0.276724	0.389432
17	6	0	2.678164	0.170793	-0.879911
18	1	0	2.373084	1.216883	-0.789120
19	1	0	3.321414	0.072230	-1.759118
20	1	0	1.785916	-0.440466	-1.045704
21	6	0	4.648791	0.578742	0.621494
22	1	0	4.371416	1.632296	0.711297
23	1	0	5.149627	0.274870	1.544055
24	1	0	5.352265	0.473310	-0.208733
25	6	0	3.782872	-1.757678	0.294278
26	1	0	4.294449	-2.076852	1.205773
27	1	0	2.885465	-2.371757	0.170019
28	1	0	4.442093	-1.942247	-0.558946
29	8	0	2.573369	-0.064944	1.527569

1a-tBuO- (dimer)

Center Number	Atomic Number	Atomic Type	Coordinates (Angstroms) X	Y	Z
1	6	0	2.123329	2.628764	0.443540
2	6	0	0.701185	3.154524	0.420888
3	6	0	0.366427	4.447619	0.820228
4	6	0	-0.307592	2.283475	0.007498
5	6	0	-0.960522	4.865068	0.817145
6	1	0	1.147289	5.136888	1.128203
7	6	0	-1.633770	2.701963	0.008726
8	1	0	-0.007773	1.294528	-0.337143
9	6	0	-1.965417	3.991305	0.414202
10	1	0	-1.208747	5.874897	1.126831
11	1	0	-2.411437	2.019845	-0.319165
12	1	0	-3.000318	4.317401	0.408978
13	1	0	2.386243	2.289867	1.451919
14	1	0	2.175540	1.713923	-0.223299
15	6	0	3.113674	3.607148	0.034275
16	7	0	3.905195	4.377476	-0.298637
17	6	0	2.063849	0.208413	-2.420213
18	6	0	0.979866	1.033242	-3.160799
19	1	0	0.981915	2.062803	-2.786668
20	1	0	1.129175	1.058690	-4.248894

21	1	0	-0.007929	0.605352	-2.955684
22	6	0	3.442724	0.830975	-2.757147
23	1	0	3.487002	1.861926	-2.389217
24	1	0	4.230377	0.259915	-2.254420
25	1	0	3.654250	0.843969	-3.835082
26	6	0	2.040891	-1.230105	-2.990455
27	1	0	2.818098	-1.826740	-2.501195
28	1	0	1.072565	-1.693601	-2.771909
29	1	0	2.207781	-1.263074	-4.076242
30	8	0	1.840672	0.203576	-1.071476

1a-tBuTO- (ts)

Center Number	Atomic Number	Atomic Type	Coordinates (Angstroms) X	Y	Z
1	6	0	2.071092	2.530628	0.487516
2	6	0	0.636559	2.971538	0.421748
3	6	0	0.238808	4.267296	0.094261
4	6	0	-0.355075	2.014245	0.664001
5	6	0	-1.110645	4.603439	0.023385
6	1	0	0.990391	5.023311	-0.109860
7	6	0	-1.699747	2.350169	0.602933
8	1	0	-0.051482	0.993460	0.872682
9	6	0	-2.086761	3.649925	0.282600
10	1	0	-1.395896	5.618210	-0.234577
11	1	0	-2.451644	1.591084	0.792803
12	1	0	-3.138010	3.911880	0.228911
13	1	0	2.313920	2.139163	1.480268
14	1	0	2.213029	1.581660	-0.246038
15	6	0	3.033331	3.546731	0.145476
16	7	0	3.814633	4.339598	-0.166055
17	6	0	2.244820	0.431383	-2.385792
18	6	0	1.421468	1.664628	-2.829894
19	1	0	1.878342	2.584644	-2.451402
20	1	0	1.349426	1.747313	-3.921810
21	1	0	0.411054	1.599181	-2.413949
22	6	0	3.682230	0.582815	-2.935583
23	1	0	4.157947	1.464315	-2.491850
24	1	0	4.272686	-0.295623	-2.653393
25	1	0	3.710292	0.685996	-4.028854
26	6	0	1.609728	-0.822609	-3.024114
27	1	0	2.170259	-1.711634	-2.715937
28	1	0	0.580789	-0.931099	-2.665434
29	1	0	1.594707	-0.778857	-4.121431
30	8	0	2.258292	0.307238	-1.015822

12b

Center Number	Atomic Number	Atomic Type	Coordinates (Angstroms) X	Y	Z
1	6	0	2.340348	-0.361271	0.899807
2	6	0	0.922014	-0.278309	0.366591
3	6	0	0.498562	0.906176	-0.232365
4	6	0	0.014355	-1.320688	0.535097
5	6	0	-0.817788	1.050577	-0.653721
6	1	0	1.201191	1.722044	-0.372983
7	6	0	-1.300278	-1.178608	0.109578
8	1	0	0.331847	-2.243643	1.008209
9	6	0	-1.720466	0.007110	-0.482454
10	1	0	-1.135546	1.975887	-1.120567
11	1	0	-1.998332	-1.996537	0.245414
12	1	0	-2.746923	0.116391	-0.813420
13	1	0	2.990025	0.238545	0.252093
14	6	0	2.874547	-1.723405	0.864790
15	7	0	3.294212	-2.793572	0.833491
16	6	0	2.459377	0.246000	2.340165
17	8	0	3.800461	0.227796	2.774911
18	1	0	2.093873	1.276293	2.252643
19	6	0	1.606503	-0.486268	3.346381
20	6	0	0.300783	-0.062653	3.584504
21	6	0	2.090672	-1.613911	4.006428
22	6	0	-0.517442	-0.761176	4.463465
23	1	0	-0.083068	0.813057	3.070646
24	6	0	1.272830	-2.311013	4.887743
25	1	0	3.108388	-1.940760	3.831494
26	6	0	-0.032580	-1.889149	5.115883
27	1	0	-1.532422	-0.423096	4.639056
28	1	0	1.657421	-3.187353	5.397150
29	1	0	-0.668561	-2.435364	5.803545
30	1	0	4.334941	0.760953	2.182983

12a

Center Number	Atomic Number	Atomic Type	Coordinates (Angstroms) X	Y	Z
1	6	0	0.416366	0.198601	-0.554350
2	6	0	1.909671	0.073820	-0.307709
3	6	0	2.612310	-0.999389	-0.851416
4	6	0	2.577241	0.980027	0.516488
5	6	0	3.965465	-1.166212	-0.574867

6	1	0	2.099795	-1.707292	-1.494305
7	6	0	3.928146	0.814042	0.790784
8	1	0	2.042563	1.826631	0.935536
9	6	0	4.624939	-0.260349	0.246346
10	1	0	4.502956	-2.002974	-1.005867
11	1	0	4.438801	1.527275	1.427401
12	1	0	5.680068	-0.387539	0.459322
13	1	0	0.132169	-0.430436	-1.403455
14	6	0	0.045905	1.574560	-0.886118
15	7	0	-0.237810	2.664603	-1.119088
16	6	0	-0.405519	-0.301222	0.682678
17	8	0	-0.031832	-1.624507	0.987880
18	1	0	-0.178696	0.367106	1.523318
19	6	0	-1.888178	-0.268188	0.404653
20	6	0	-2.657893	0.809786	0.832141
21	6	0	-2.491383	-1.294815	-0.320467
22	6	0	-4.014940	0.867185	0.534901
23	1	0	-2.195265	1.612651	1.396915
24	6	0	-3.847982	-1.240451	-0.612914
25	1	0	-1.897429	-2.143327	-0.640410
26	6	0	-4.612385	-0.158075	-0.187963
27	1	0	-4.604454	1.712419	0.871126
28	1	0	-4.310880	-2.045584	-1.172456
29	1	0	-5.671104	-0.116399	-0.417675
30	1	0	0.910216	-1.644609	1.178004

----

tBuOH…13

Center Number	Atomic Number	Atomic Type	Coordinates (Angstroms) X	Y	Z
1	6	0	-0.452071	-1.343084	-1.125871
2	6	0	0.440987	-0.921894	-0.000505
3	1	0	-0.227454	-0.722312	-2.006673
4	1	0	0.252503	1.127051	-0.541551
5	6	0	-0.055901	-0.932992	1.305082
6	7	0	-0.447217	-0.890627	2.404827
7	6	0	1.870844	-0.791220	-0.196692
8	6	0	2.463164	-0.864912	-1.478069
9	6	0	2.753519	-0.539762	0.880181
10	6	0	3.821691	-0.657371	-1.667143
11	1	0	1.848895	-1.102164	-2.338638
12	6	0	4.109361	-0.336846	0.682660
13	1	0	2.353790	-0.502663	1.888714
14	6	0	4.666588	-0.385288	-0.594404
15	1	0	4.227987	-0.717800	-2.672890
16	1	0	4.743630	-0.141105	1.542631
17	1	0	5.728689	-0.227541	-0.746486
18	6	0	-1.928622	-1.159522	-0.818769

19	6	0	-2.555926	0.056215	-1.087620
20	6	0	-2.673225	-2.189989	-0.249660
21	6	0	-3.900786	0.239004	-0.788697
22	1	0	-1.973402	0.871546	-1.505351
23	6	0	-4.019190	-2.009772	0.049631
24	1	0	-2.182802	-3.132295	-0.035426
25	6	0	-4.638673	-0.794515	-0.219835
26	1	0	-4.373458	1.193295	-0.997441
27	1	0	-4.582558	-2.818929	0.502470
28	1	0	-5.687941	-0.651711	0.016114
29	8	0	-0.176699	-2.715600	-1.494147
30	1	0	-0.675522	-2.907226	-2.292757
31	6	0	0.047636	2.910092	0.237461
32	6	0	-1.251640	2.642953	1.002617
33	1	0	-1.274033	1.613314	1.370947
34	1	0	-1.343613	3.316377	1.860881
35	1	0	-2.114981	2.793363	0.348289
36	6	0	1.253447	2.695669	1.153982
37	1	0	1.229229	1.691345	1.583348
38	1	0	2.183289	2.799541	0.588033
39	1	0	1.253382	3.418588	1.976023
40	6	0	0.052714	4.320663	-0.341632
41	1	0	0.979068	4.499797	-0.894400
42	1	0	-0.788392	4.449912	-1.028895
43	1	0	-0.031012	5.066260	0.454390
44	8	0	0.136903	2.032985	-0.884050

----

ts23 (14_15)

Center Number	Atomic Number	Atomic Type	Coordinates (Angstroms) X	Y	Z
1	6	0	-0.504081	-0.458648	0.334353
2	6	0	0.575376	-0.236806	-0.558332
3	6	0	0.344788	-0.077373	-1.974167
4	7	0	0.225815	0.054710	-3.109572
5	6	0	1.963885	-0.081287	-0.102532
6	6	0	2.232506	0.467506	1.158611
7	6	0	3.016397	-0.466572	-0.943887
8	6	0	3.544637	0.610996	1.576060
9	1	0	1.428746	0.823240	1.792505
10	6	0	4.323425	-0.328868	-0.511087
11	1	0	2.813566	-0.878985	-1.925925
12	6	0	4.586861	0.207502	0.746330
13	1	0	3.756855	1.052001	2.541804
14	1	0	5.138453	-0.633602	-1.155093
15	1	0	5.612048	0.324458	1.076556
16	6	0	-1.939107	-0.397493	0.114410
17	6	0	-2.747999	-1.019157	1.080931

18	6	0	-2.535136	0.273952	-0.964136
19	6	0	-4.124983	-0.995547	0.954977
20	1	0	-2.291293	-1.524018	1.924961
21	6	0	-3.913779	0.302565	-1.074679
22	1	0	-1.937581	0.793900	-1.700208
23	6	0	-4.706667	-0.336277	-0.124171
24	1	0	-4.744785	-1.481571	1.697715
25	1	0	-4.373922	0.830245	-1.900428
26	1	0	-5.785586	-0.310581	-0.220821
27	1	0	-0.184564	-0.646491	1.358056
28	1	0	0.087703	-1.462499	-0.263006

ts24 (14-2b)

Center Number	Atomic Number	Atomic Type	Coordinates (Angstroms) X	Y	Z
1	6	0	-1.079789	-0.840044	0.674330
2	6	0	-0.571303	0.560486	0.974988
3	1	0	-0.312148	-1.359298	0.094667
4	6	0	-1.001014	1.123815	2.226081
5	7	0	-1.363780	1.538564	3.239060
6	6	0	-0.053387	1.419167	-0.000244
7	6	0	0.015659	1.061848	-1.383887
8	6	0	0.694341	2.555592	0.446568
9	6	0	0.620764	1.890853	-2.274469
10	1	0	-0.468184	0.157008	-1.727629
11	6	0	1.238204	3.451666	-0.528264
12	1	0	0.489514	2.950779	1.433306
13	6	0	1.221234	3.112188	-1.841940
14	1	0	0.632410	1.637153	-3.327950
15	1	0	1.689236	4.377102	-0.193619
16	1	0	1.653584	3.777142	-2.581269
17	6	0	-2.401383	-0.880751	-0.069776
18	6	0	-2.523723	-1.653005	-1.223041
19	6	0	-3.512968	-0.172143	0.386962
20	6	0	-3.730587	-1.715986	-1.911486
21	1	0	-1.666748	-2.211517	-1.587570
22	6	0	-4.718403	-0.231487	-0.300740
23	1	0	-3.443957	0.430315	1.286774
24	6	0	-4.831116	-1.002791	-1.452980
25	1	0	-3.808048	-2.321379	-2.807627
26	1	0	-5.572847	0.325958	0.066047
27	1	0	-5.772224	-1.047353	-1.989096
28	1	0	-1.173758	-1.381985	1.619303
29	6	0	3.236211	-3.011920	-0.692123
30	6	0	2.866684	-3.145686	0.644048
31	6	0	2.516272	-2.023295	1.382781
32	6	0	2.537810	-0.757218	0.796668

33	6	0	2.909851	-0.627239	-0.537703
34	6	0	3.258622	-1.751461	-1.278942
35	1	0	3.509388	-3.887535	-1.270397
36	1	0	2.852429	-4.125403	1.108656
37	1	0	2.224008	-2.128309	2.424076
38	1	0	2.923938	0.361815	-0.980454
39	1	0	3.551291	-1.642394	-2.317631
40	6	0	2.082914	0.445897	1.587410
41	1	0	0.906438	0.234921	1.618796
42	1	0	2.316380	0.391090	2.663298
43	8	0	2.276293	1.631584	1.045764

ts25 (15-2b)

Center Number	Atomic Number	Atomic Type	Coordinates (Angstroms) X	Y	Z
1	6	0	-0.674734	0.739776	-0.383691
2	6	0	-1.551747	0.151376	0.609833
3	1	0	-1.198877	1.020534	-1.296542
4	6	0	-1.370597	0.499661	1.987536
5	7	0	-1.249760	0.744666	3.108745
6	6	0	-2.960838	-0.162062	0.200583
7	6	0	-3.216210	-0.673386	-1.076085
8	6	0	-4.035737	0.014499	1.073623
9	6	0	-4.510979	-0.975746	-1.474881
10	1	0	-2.399438	-0.855681	-1.766398
11	6	0	-5.330698	-0.291976	0.673411
12	1	0	-3.862832	0.399474	2.072411
13	6	0	-5.576494	-0.784257	-0.602484
14	1	0	-4.685415	-1.372551	-2.468730
15	1	0	-6.151256	-0.141157	1.365808
16	1	0	-6.587222	-1.022990	-0.912576
17	6	0	0.478774	1.638793	-0.104633
18	6	0	0.776903	2.633915	-1.039641
19	6	0	1.331603	1.466237	0.987980
20	6	0	1.889139	3.448736	-0.880239
21	1	0	0.129667	2.770972	-1.900147
22	6	0	2.448978	2.274429	1.141640
23	1	0	1.139097	0.692218	1.718321
24	6	0	2.731368	3.267429	0.211024
25	1	0	2.100129	4.221489	-1.610593
26	1	0	3.104362	2.120980	1.990932
27	1	0	3.605379	3.896775	0.334610
28	1	0	-0.984113	-1.169257	0.573370
29	6	0	4.509299	-0.766743	-0.005856
30	6	0	3.952989	-0.444865	-1.241050
31	6	0	2.623279	-0.740728	-1.498433
32	6	0	1.837216	-1.349334	-0.518544

33	6	0	2.397655	-1.672398	0.715924
34	6	0	3.733234	-1.387302	0.966947
35	1	0	5.548698	-0.534052	0.196299
36	1	0	4.555650	0.042234	-1.998771
37	1	0	2.183247	-0.477882	-2.455916
38	1	0	1.777381	-2.141386	1.470755
39	1	0	4.167551	-1.642632	1.926793
40	6	0	0.393542	-1.580712	-0.774523
41	1	0	-0.047595	-0.281413	-0.873122
42	1	0	0.121585	-1.849611	-1.803542
43	8	0	-0.366731	-2.044354	0.172770

D1 (3a'…2a)

Center Number	Atomic Number	Atomic Type	Coordinates (Angstroms) X	Y	Z
1	6	0	0.937174	1.234040	0.117047
2	6	0	1.263338	0.298815	1.029369
3	1	0	1.228985	0.996580	-0.901475
4	6	0	1.042861	0.475723	2.432881
5	7	0	0.894640	0.573537	3.572391
6	6	0	1.855711	-1.005514	0.631292
7	6	0	1.451909	-1.613577	-0.561347
8	6	0	2.797654	-1.646222	1.439098
9	6	0	2.007977	-2.825352	-0.950458
10	1	0	0.678437	-1.148703	-1.164899
11	6	0	3.350066	-2.857749	1.045669
12	1	0	3.104580	-1.193060	2.375852
13	6	0	2.960522	-3.448667	-0.151780
14	1	0	1.684001	-3.290339	-1.874832
15	1	0	4.086395	-3.341684	1.677075
16	1	0	3.389688	-4.396837	-0.455088
17	6	0	0.222249	2.505282	0.242201
18	6	0	-0.517380	2.898292	1.366616
19	6	0	0.235530	3.354197	-0.874819
20	6	0	-1.201460	4.105267	1.371638
21	1	0	-0.572563	2.262771	2.239879
22	6	0	-0.446232	4.562372	-0.865622
23	1	0	0.790433	3.057623	-1.759182
24	6	0	-1.166827	4.942412	0.260858
25	1	0	-1.769496	4.391396	2.249266
26	1	0	-0.417886	5.204182	-1.738468
27	1	0	-1.704446	5.883590	0.272043
28	1	0	-1.427158	1.030148	-1.320025
29	6	0	-2.458544	-0.590434	-0.926913
30	8	0	-1.423327	0.119234	-1.626578
31	6	0	-2.136075	-0.635885	0.567187
32	1	0	-2.061604	0.375946	0.978600

33	1	0	-2.919876	-1.165484	1.115438
34	1	0	-1.187881	-1.150039	0.738741
35	6	0	-3.797298	0.105778	-1.169240
36	1	0	-4.008910	0.160649	-2.239930
37	1	0	-4.611883	-0.434358	-0.679316
38	1	0	-3.779863	1.124146	-0.766399
39	6	0	-2.456077	-1.992990	-1.520706
40	1	0	-3.221990	-2.610196	-1.044698
41	1	0	-2.657172	-1.952709	-2.593997
42	1	0	-1.484730	-2.469481	-1.365113

3a'…tBuOH

Center Number	Atomic Number	Atomic Type	Coordinates (Angstroms) X	Y	Z
1	6	0	1.082271	-0.120112	-0.769880
2	6	0	0.318261	0.902282	-0.125314
3	1	0	0.472055	-0.903460	-1.211281
4	6	0	0.865484	1.598975	1.008882
5	7	0	1.261524	2.134236	1.948032
6	6	0	-1.134127	1.053216	-0.344511
7	6	0	-1.700387	0.661894	-1.562922
8	6	0	-1.952604	1.604709	0.642641
9	6	0	-3.059401	0.812874	-1.780919
10	1	0	-1.069573	0.276423	-2.354447
11	6	0	-3.317209	1.744489	0.423361
12	1	0	-1.526513	1.924961	1.586422
13	6	0	-3.873133	1.351969	-0.787027
14	1	0	-3.486082	0.518934	-2.732790
15	1	0	-3.943181	2.168406	1.199632
16	1	0	-4.936317	1.470823	-0.961304
17	6	0	2.397879	-0.583526	-0.237505
18	6	0	3.462641	0.300988	-0.048283
19	6	0	2.588545	-1.936067	0.050219
20	6	0	4.679734	-0.155512	0.440787
21	1	0	3.334248	1.348779	-0.297576
22	6	0	3.808890	-2.393834	0.532647
23	1	0	1.771878	-2.635000	-0.100612
24	6	0	4.857584	-1.503456	0.733795
25	1	0	5.495152	0.544236	0.585907
26	1	0	3.938709	-3.447286	0.754483
27	1	0	5.809517	-1.858566	1.112061
28	1	0	1.309036	0.799409	-1.671633
29	6	0	1.004500	3.362335	-2.211622
30	8	0	1.097009	2.107571	-1.560254
31	6	0	-0.010539	4.198983	-1.426832
32	1	0	-0.991476	3.716484	-1.432197
33	1	0	0.314116	4.315829	-0.389365

34	1	0	-0.109405	5.192760	-1.872158
35	6	0	0.514749	3.135025	-3.643683
36	1	0	0.428249	4.082801	-4.183532
37	1	0	1.215738	2.493690	-4.185635
38	1	0	-0.464870	2.649759	-3.631805
39	6	0	2.380202	4.031273	-2.200638
40	1	0	3.107757	3.402901	-2.722282
41	1	0	2.351723	5.008211	-2.693335
42	1	0	2.720155	4.170348	-1.170936

ts22a (2a-3a')

Center Number	Atomic Number	Atomic Type	Coordinates (Angstroms) X	Y	Z
1	6	0	1.236486	-0.295900	-0.522486
2	6	0	0.799271	0.879113	0.163220
3	1	0	0.436263	-0.828068	-1.029276
4	6	0	1.524032	1.326441	1.322472
5	7	0	2.056673	1.665495	2.285097
6	6	0	-0.495401	1.525072	-0.086863
7	6	0	-1.214113	1.247674	-1.257316
8	6	0	-1.019873	2.440132	0.830601
9	6	0	-2.436016	1.854603	-1.487076
10	1	0	-0.806165	0.579878	-2.005545
11	6	0	-2.249544	3.038895	0.599823
12	1	0	-0.465438	2.688303	1.727550
13	6	0	-2.961214	2.746670	-0.555330
14	1	0	-2.979216	1.638584	-2.399629
15	1	0	-2.642902	3.746159	1.319915
16	1	0	-3.918468	3.221182	-0.738853
17	6	0	2.300469	-1.184768	0.037761
18	6	0	3.596580	-0.718261	0.265170
19	6	0	2.005659	-2.519687	0.318264
20	6	0	4.568282	-1.564217	0.784286
21	1	0	3.839760	0.310801	0.024181
22	6	0	2.979789	-3.367781	0.831434
23	1	0	1.003630	-2.896082	0.138007
24	6	0	4.263771	-2.890774	1.069821
25	1	0	5.569483	-1.186741	0.959246
26	1	0	2.733818	-4.401478	1.047530
27	1	0	5.024460	-3.550595	1.471521
28	1	0	1.773795	0.514002	-1.364238
29	6	0	-0.313214	5.989273	-0.098678
30	6	0	-0.630507	5.441061	-1.335793
31	6	0	0.217225	4.508288	-1.922896
32	6	0	1.392710	4.117419	-1.286329
33	6	0	1.698265	4.665049	-0.040073
34	6	0	0.852103	5.595496	0.551264

35	1	0	-0.973124	6.717451	0.360078
36	1	0	-1.542978	5.734903	-1.843126
37	1	0	-0.037699	4.076847	-2.885786
38	1	0	2.605022	4.355776	0.469941
39	1	0	1.103724	6.014698	1.519346
40	6	0	2.295440	3.061174	-1.884694
41	1	0	2.093264	2.970990	-2.959974
42	1	0	3.346708	3.357314	-1.761084
43	8	0	2.042925	1.837428	-1.243728

ts22b (2a-3a')

Center Number	Atomic Number	Atomic Type	Coordinates (Angstroms) X	Y	Z
1	6	0	-0.946890	0.904490	0.845435
2	6	0	-1.403273	0.050747	-0.208758
3	1	0	-1.735835	1.202563	1.530014
4	6	0	-0.714518	0.046302	-1.470678
5	7	0	-0.207876	0.053728	-2.504379
6	6	0	-2.720245	-0.607732	-0.185931
7	6	0	-3.379013	-0.831650	1.029849
8	6	0	-3.334341	-1.009247	-1.375971
9	6	0	-4.627077	-1.429603	1.047500
10	1	0	-2.900365	-0.570694	1.965125
11	6	0	-4.591535	-1.598356	-1.353267
12	1	0	-2.836018	-0.852403	-2.325540
13	6	0	-5.240377	-1.810025	-0.144080
14	1	0	-5.122383	-1.608033	1.994656
15	1	0	-5.060085	-1.895290	-2.284022
16	1	0	-6.218740	-2.276238	-0.126103
17	6	0	0.096880	1.955215	0.633787
18	6	0	1.365122	1.651579	0.133894
19	6	0	-0.187800	3.276153	0.985749
20	6	0	2.316836	2.650294	-0.027005
21	1	0	1.619118	0.630595	-0.122460
22	6	0	0.765515	4.274515	0.826813
23	1	0	-1.166432	3.524828	1.384183
24	6	0	2.021571	3.964706	0.316606
25	1	0	3.295289	2.391049	-0.415698
26	1	0	0.525344	5.295767	1.101037
27	1	0	2.766536	4.742617	0.192539
28	1	0	-0.356223	-0.134664	1.300739
29	6	0	4.520507	-0.742886	-0.377343
30	6	0	4.149675	-0.944549	0.948476
31	6	0	2.929335	-1.539664	1.245522
32	6	0	2.063090	-1.936179	0.226900
33	6	0	2.450473	-1.743715	-1.097993
34	6	0	3.670222	-1.148011	-1.400743

35	1	0	5.469888	-0.274080	-0.611732
36	1	0	4.809307	-0.632523	1.750697
37	1	0	2.635091	-1.682122	2.281061
38	1	0	1.780356	-2.036050	-1.899647
39	1	0	3.952546	-0.993339	-2.436168
40	6	0	0.679698	-2.440403	0.565580
41	1	0	0.297002	-3.075500	-0.245873
42	1	0	0.706164	-3.062472	1.471270
43	8	0	-0.219611	-1.380142	0.747415

D3 (15…2b)

Center Number	Atomic Number	Atomic Type	Coordinates (Angstroms) X	Y	Z
1	6	0	-0.945224	-0.798632	0.855940
2	6	0	-0.428878	0.629501	0.914511
3	1	0	-0.274766	-1.378830	0.212123
4	6	0	-0.025366	1.336412	-0.205760
5	6	0	-0.062064	0.829839	-1.546701
6	6	0	0.757695	2.511763	0.055352
7	6	0	0.449367	1.561307	-2.569556
8	1	0	-0.559566	-0.110564	-1.751311
9	6	0	1.184266	3.312072	-1.059703
10	1	0	0.599600	3.017114	1.000908
11	6	0	1.057987	2.832310	-2.321121
12	1	0	0.377636	1.197214	-3.587751
13	1	0	1.640807	4.275424	-0.869896
14	1	0	1.406194	3.420760	-3.162983
15	6	0	-2.367618	-0.907033	0.311354
16	6	0	-2.637179	-1.766826	-0.748020
17	6	0	-3.397734	-0.148333	0.862291
18	6	0	-3.926316	-1.863215	-1.260829
19	1	0	-1.838842	-2.366076	-1.174487
20	6	0	-4.684461	-0.245402	0.350674
21	1	0	-3.198365	0.518871	1.694369
22	6	0	-4.950877	-1.101458	-0.713528
23	1	0	-4.127440	-2.534723	-2.087528
24	1	0	-5.481369	0.347901	0.783889
25	1	0	-5.955827	-1.174867	-1.112895
26	6	0	3.109644	-3.135401	-0.572374
27	6	0	2.855592	-3.085678	0.796374
28	6	0	2.599694	-1.867371	1.411527
29	6	0	2.597891	-0.686454	0.667053
30	6	0	2.852015	-0.740435	-0.699874
31	6	0	3.109085	-1.961007	-1.316690
32	1	0	3.309654	-4.086332	-1.053684
33	1	0	2.854598	-3.997407	1.383374
34	1	0	2.389805	-1.832194	2.476786
35	1	0	2.844202	0.182983	-1.266858

36	1	0	3.310234	-1.994191	-2.382110
37	6	0	2.226446	0.619599	1.326592
38	1	0	1.053709	0.437412	1.499618
39	1	0	2.575277	0.707297	2.367020
40	8	0	2.364585	1.719316	0.604661
41	6	0	-0.854633	-1.409013	2.187879
42	7	0	-0.781485	-1.870442	3.238756
43	1	0	-0.658031	1.186489	1.819065

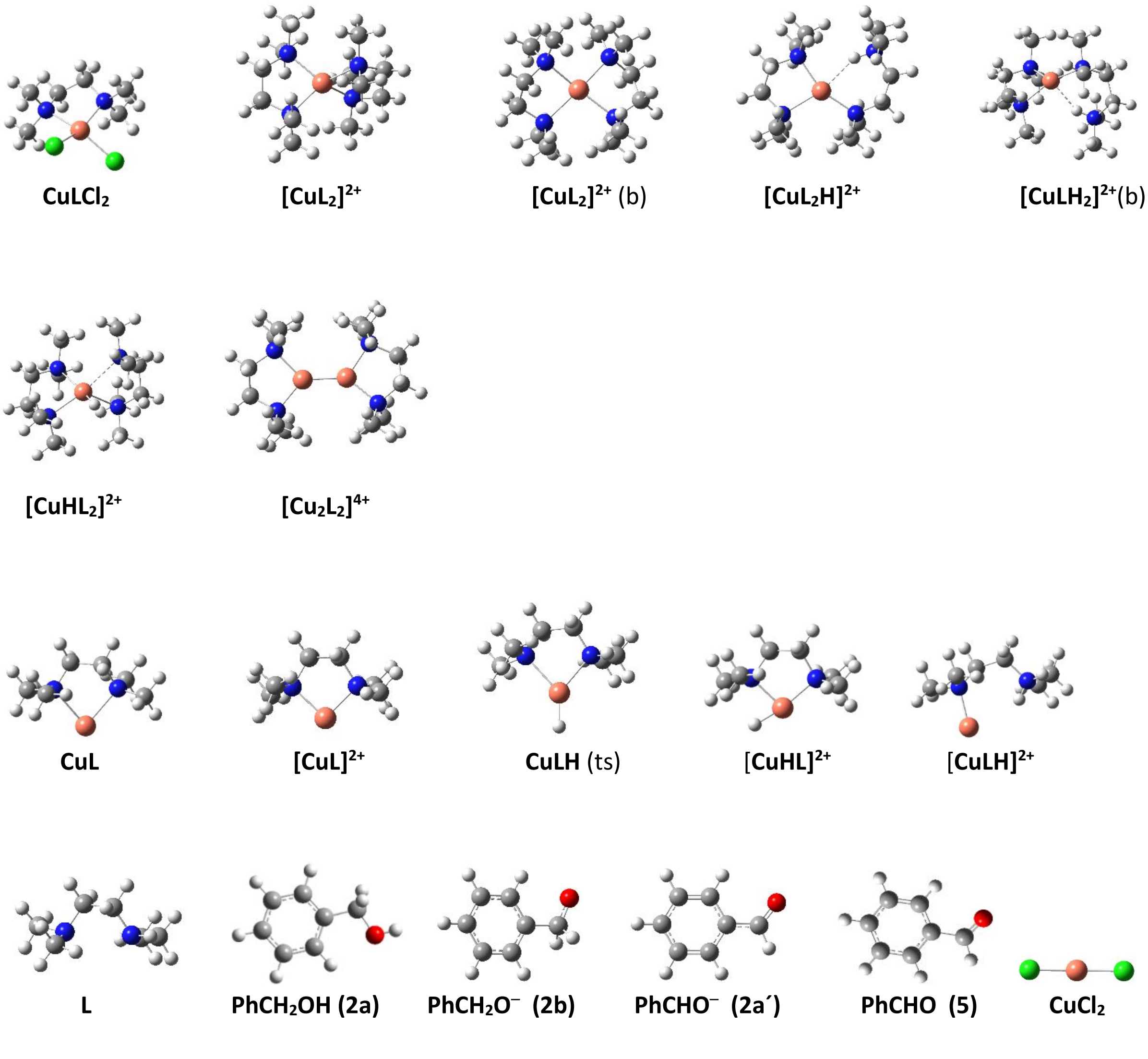

Figure S2. Calculated minimum and transition states (ts) structures.

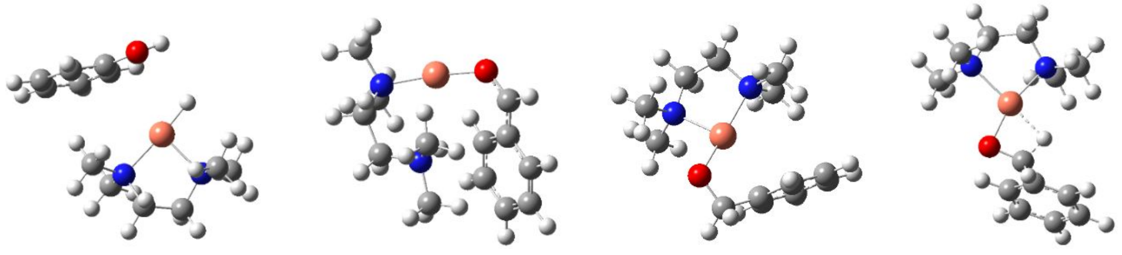

2a´…HCu^{III}L **2a´-HCu^{II}L** **2b-CuL** **2b-CuL (ts)**

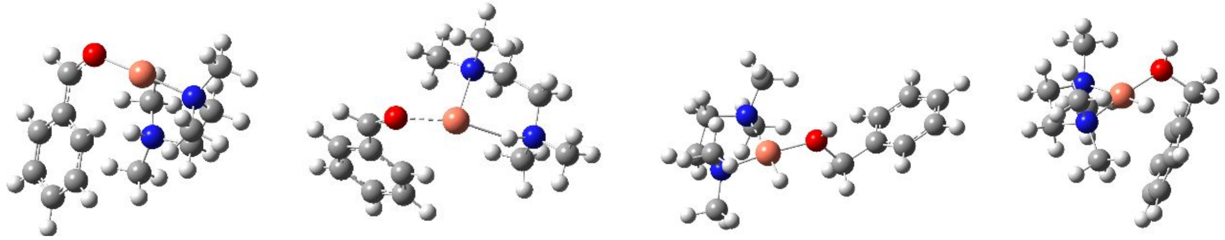

2a´-Cu^{II}LH **5...Cu^{II}LH** **2a...CuHL(ts11)** **ts12**

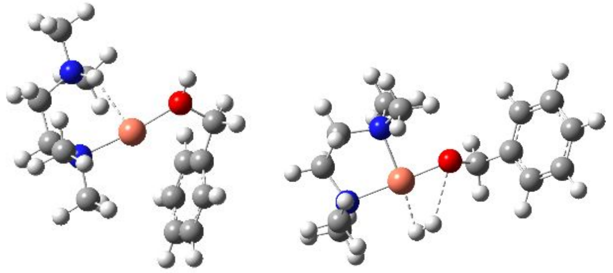

2a-Cu^{II}LH **ts13**

Figure S3. Calculated structures including the CuL catalytic system.

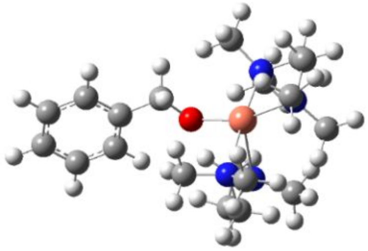

2b-CuL$_2$

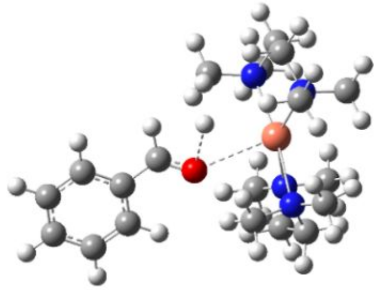

2b-CuL$_2$

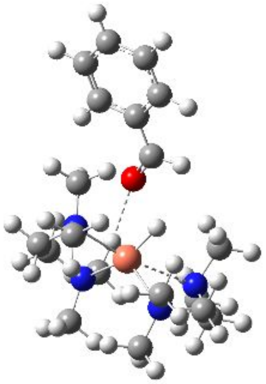

5…HCuL$_2$

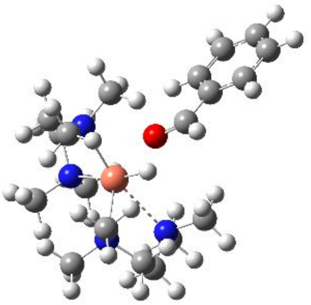

5…HCuL$_2$ (b)

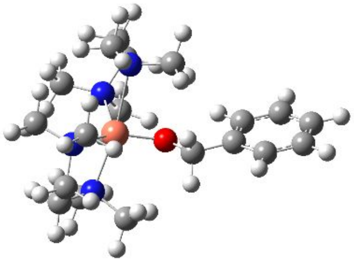

2b-CuL$_2$

Figure S4. Calculated structures including the CuL$_2$ catalytic system.

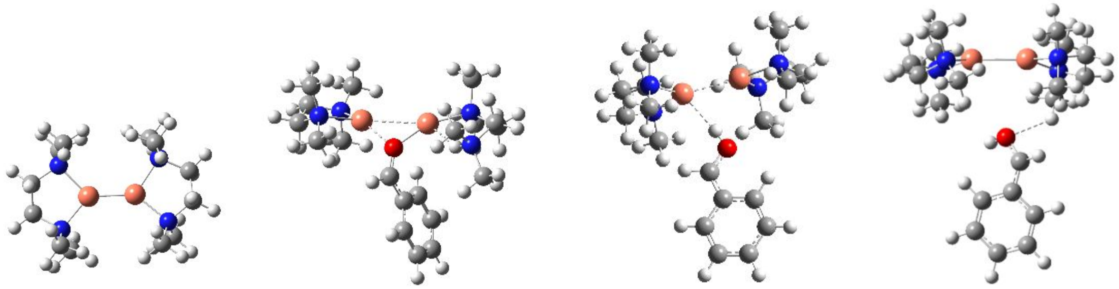

[Cu_2L_2]$^{4+}$ 2a´-Cu_2L_2H 2a´-HCu_2L_2 2a´H…Cu_2L_2

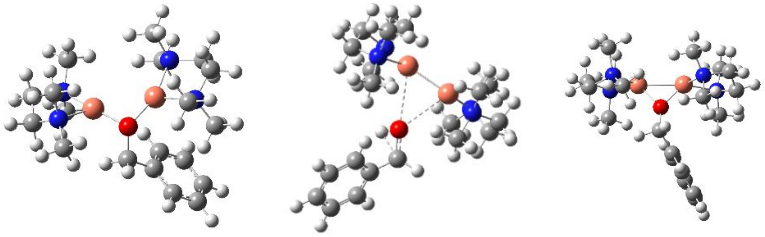

2b-Cu_2L_2 ts8 ts7

Figure S5. Calculated structures including the Cu_2L_2 catalytic system.

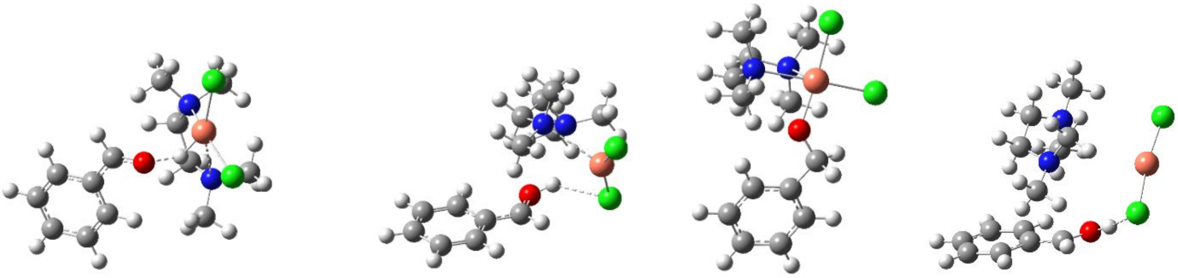

2a´…HCuLCl_2 2a´H…CuLCl_2 2b-CuLCl_2 2a´H…CuCl_2…L

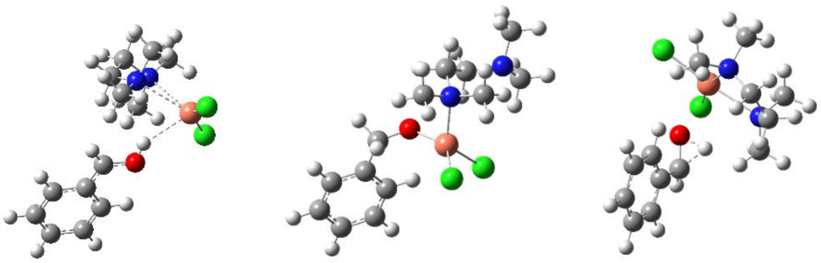

2a´H…CuCl_2L 2b-CuLCl_2 2bH…CuLCl_2

Figure S6. Calculated structures including the CuLCl_2 catalytic system.

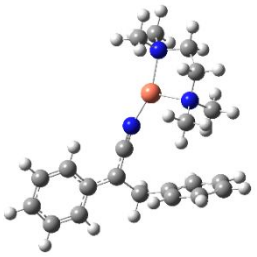

3a´H-CuL

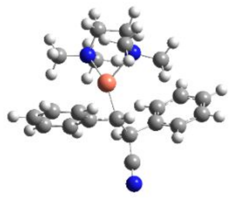

3a´-CuLH

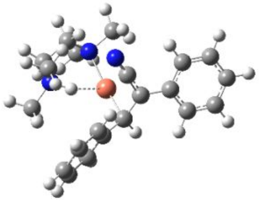

3a´…CuLH (trans)

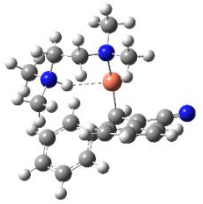

3a´…CuLH (cis)

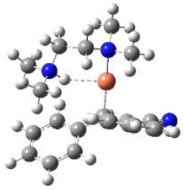

3a´…CuLH

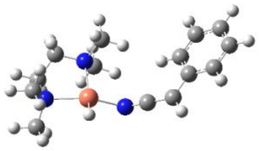

[PhCHCN-CuHL]^{0}

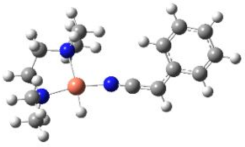

PhCHCN-CuHL

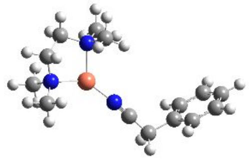

1a-CuL

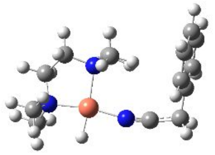

1a-CuHL

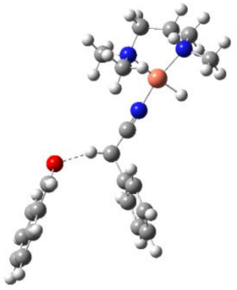

5…1a-CuLH

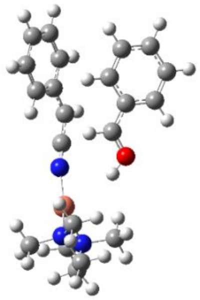

ts15

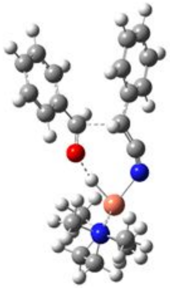

ts14

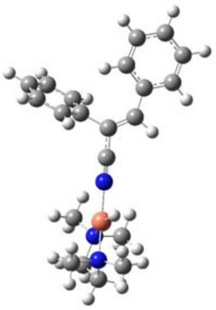

3a'-CuLH

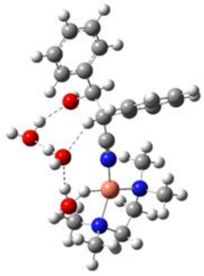

3a'-CuL-3W

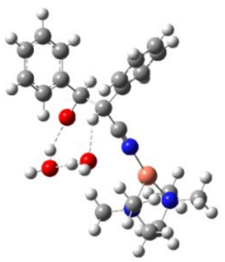

3a'-CuL-2W

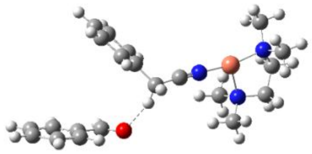

5…1a-CuL

Figure S7. Calculated structures including the CuL catalytic system.

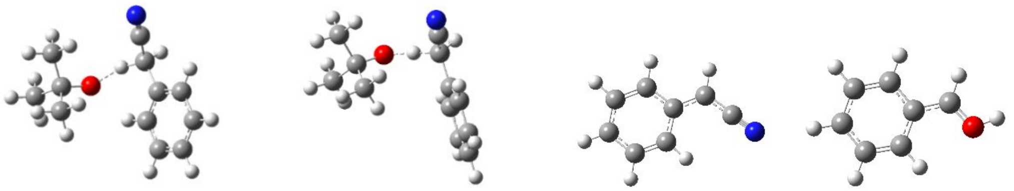

1a…tBuO- (min-dimer) **1a-tBuO- (ts16)** **$PhCHCN^-$** (**10**) **$PhCHOH^+$ (11)**

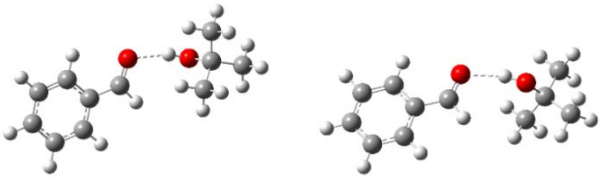

5…tBuOH (min-dimer) **5…tBuOH (ts17)**

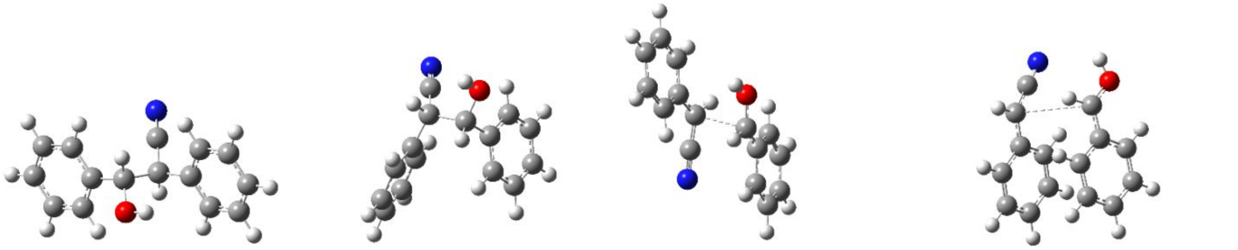

12a **12a** **$PhCHCN^-$…$PhCHOH^+$(ts18a)** **$PhCHCN^-$…$PhCHOH^+$(ts18b)**

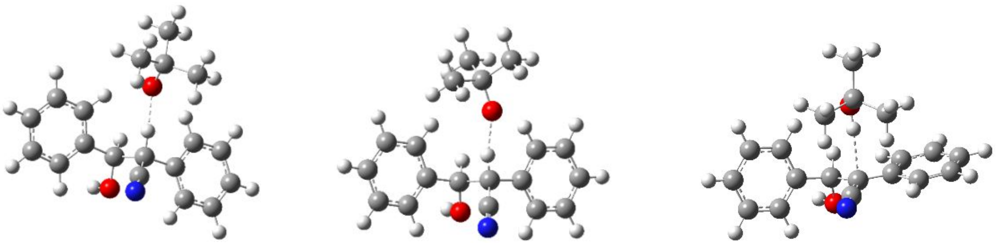

$tBuO^-$…12a (dimer) **ts19** **tBuOH….13**

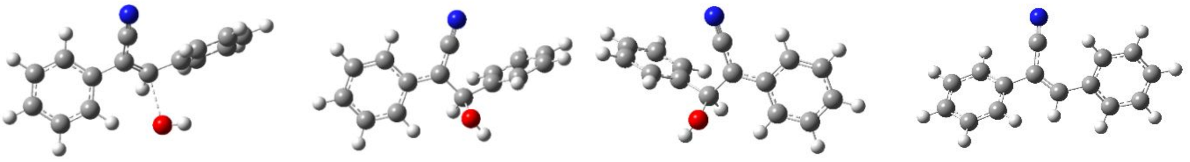

3a'…OH **13** (two points of view) **3a'**

Figure S8. Calculated structures involved in catalytic cycle **B**.

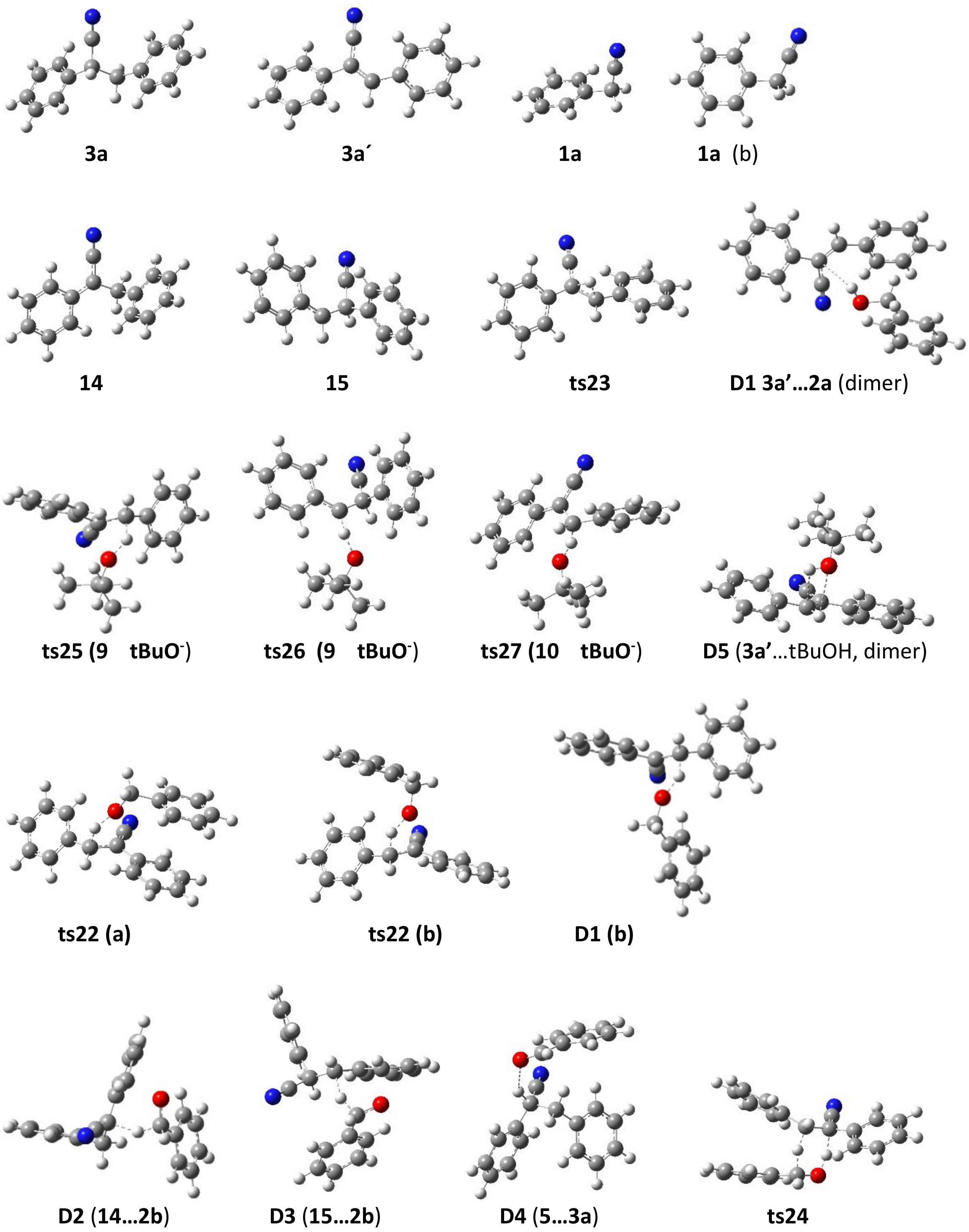

Figure S9. Calculated structures involved in catalytic cycle **C**.

2. References

(1) Rong, W.; Liu, D.; Zuo, H.; Pan, Y.; Jian, Z.; Li, S.; Cui, D. Rare-Earth-Metal Complexes Bearing Phosphazene Ancillary Ligands: Structures and Catalysis toward Highly Trans-1,4-Selective (Co)Polymerizations of Conjugated Dienes. *Organometallics* **2013**, *32*, 1166–1175.

(2) Petersson, M. J.; Loughlin, W. A.; Jenkins, I. D. Selective Mono Reduction of Bis-Phosphine Oxides under Mild Conditions. *Chem. Commun.* **2008**, 4493–4494.

(3) Putta, R. R.; Chun, S.; Lee, S. B.; Hong, J.; Choi, S. H.; Oh, D.-C.; Hong, S. Chemoselective α-Alkylation and α-Olefination of Arylacetonitriles with Alcohols via Iron-Catalyzed Borrowing Hydrogen and Dehydrogenative Coupling. *J. Org. Chem.* **2022**, *87* (24), 16378–16389.

(4) Li, C.; Bai, L.; Ge, M.-T.; Xia, A.-B.; Wang, Y.; Qiu, Y.-R.; Xu, D.-Q. Base-Controlled Chemoselectivity: Direct Coupling of Alcohols and Acetonitriles to Synthesise α-Alkylated Arylacetonitriles or Acetamides. *New J. Chem.* **2021**, *45*, 15200–15204.

(5) Zhu, Z.-H.; Li, Y.; Wang, Y.-B.; Lan, Z.-G.; Zhu, X.; Hao, X.-Q.; Song, M.-P. α-Alkylation of Nitriles with Alcohols Catalyzed by NNN′ Pincer Ru(II) Complexes Bearing Bipyridyl Imidazoline Ligands. *Organometallics* **2019**, *38* (9), 2156–2166.

(6) Bera, S.; Bera, A.; Banerjee, D. Nickel-Catalyzed Hydrogen-Borrowing Strategy: Chemo-Selective Alkylation of Nitriles with Alcohols. *Chem. Commun.* **2020**, *56*, 6850–6853.

(7) Gaussian 16, Revision C.01, M. J. Frisch, G. W. Trucks, H. B. Schlegel, G. E. Scuseria, M. A. Robb, J. R. Cheeseman, G. Scalmani, V. Barone, G. A. Petersson, H. Nakatsuji, X. Li, M. Caricato, A. V. Marenich, J. Bloino, B. G. Janesko, R. Gomperts, B. Mennucci, H. P. Hratchian, J. V. Ortiz, A. F. Izmaylov, J. L. Sonnenberg, D. Williams-Young, F. Ding, F. Lipparini, F. Egidi, J. Goings, B. Peng, A. Petrone, T. Henderson, D. Ranasinghe, V. G. Zakrzewski, J. Gao, N. Rega, G. Zheng, W. Liang, M. Hada, M. Ehara, K. Toyota, R. Fukuda, J. Hasegawa, M. Ishida, T. Nakajima, Y. Honda, O. Kitao, H. Nakai, T. Vreven, K. Throssell, J. A. Montgomery, Jr., J. E. Peralta, F. Ogliaro, M. J. Bearpark, J. J. Heyd, E. N. Brothers, K. N. Kudin, V. N. Staroverov, T. A. Keith, R. Kobayashi, J. Normand, K. Raghavachari, A. P. Rendell, J. C. Burant, S. S. Iyengar, J. Tomasi, M. Cossi, J. M. Millam, M. Klene, C. Adamo, R. Cammi, J. W. Ochterski, R. L. Martin, K. Morokuma, O. Farkas, J. B. Foresman, and D. J. Fox, Gaussian, Inc., Wallingford CT, **2016.**
